# *In silico* analysis of the profilaggrin sequence indicates alterations in the stability, degradation route, and intracellular protein fate in filaggrin null mutation carriers

**DOI:** 10.3389/fmolb.2023.1105678

**Published:** 2023-05-02

**Authors:** Argho Aninda Paul, Natalia A. Szulc, Adrian Kobiela, Sara J. Brown, Wojciech Pokrzywa, Danuta Gutowska-Owsiak

**Affiliations:** ^1^ Experimental and Translational Immunology Group, Intercollegiate Faculty of Biotechnology of University of Gdansk and Medical University of Gdansk, University of Gdansk, Gdansk, Poland; ^2^ Laboratory of Protein Metabolism, International Institute of Molecular and Cell Biology in Warsaw, Warsaw, Poland; ^3^ Centre for Genomic and Experimental Medicine, Institute of Genetics and Cancer, University of Edinburgh, Edinburgh, United Kingdom

**Keywords:** atopic dermatitis, filaggrin, proteasome, degron, ubiquitination

## Abstract

**Background:** Loss of function mutation in *FLG* is the major genetic risk factor for atopic dermatitis (AD) and other allergic manifestations. Presently, little is known about the cellular turnover and stability of profilaggrin, the protein encoded by *FLG*. Since ubiquitination directly regulates the cellular fate of numerous proteins, their degradation and trafficking, this process could influence the concentration of filaggrin in the skin.

**Objective:** To determine the elements mediating the interaction of profilaggrin with the ubiquitin-proteasome system (i.e., degron motifs and ubiquitination sites), the features responsible for its stability, and the effect of nonsense and frameshift mutations on profilaggrin turnover.

**Methods:** The effect of inhibition of proteasome and deubiquitinases on the level and modifications of profilaggrin and processed products was assessed by immunoblotting. Wild-type profilaggrin sequence and its mutated variants were analysed *in silico* using the DEGRONOPEDIA and Clustal Omega tool.

**Results:** Inhibition of proteasome and deubiquitinases stabilizes profilaggrin and its high molecular weight of presumably ubiquitinated derivatives. *In silico* analysis of the sequence determined that profilaggrin contains 18 known degron motifs as well as multiple canonical and non-canonical ubiquitination-prone residues. *FLG* mutations generate products with increased stability scores, altered usage of the ubiquitination marks, and the frequent appearance of novel degrons, including those promoting C-terminus-mediated degradation routes.

**Conclusion:** The proteasome is involved in the turnover of profilaggrin, which contains multiple degrons and ubiquitination-prone residues. *FLG* mutations alter those key elements, affecting the degradation routes and the mutated products’ stability.

## Introduction

Atopic dermatitis (AD) is a disease characterized by chronically relapsing-remitting skin inflammation. The etiology of AD is multifactorial, involving gene-environment interaction with strong hereditability (80%) (Larsen, 1993). Recent studies show that epidermal barrier dysfunction is a central feature in the pathogenesis of AD (Cork et al., 2009; Luger et al., 2021). Genetic studies point towards a region that harbours several genes involved in epidermal barrier maintenance (Cookson et al., 2001; Paternoster et al., 2011), known as the “epidermal differentiation complex” (EDC) (Mischke et al., 1996), spanning 1.9 Mbp within chromosome 1q21 and known to be prone to chromosomal rearrangement (Forus et al., 1998; Itoyama et al., 2002; Chen et al., 2003; Wong et al., 2003). The genes of the EDC can be grouped into S100 calcium binding proteins (Eckert et al., 2004; Marenholz et al., 2004), S100 fused-type protein (SFTP) family (Gan et al., 1990; Lee et al., 1993; Krieg et al., 1997; Contzler et al., 2005; Takaishi et al., 2005; Wu et al., 2011; Kypriotou et al., 2012), cornified envelope precursor family (Backendorf and Hohl, 1992), and small proline-rich proteins (Zhao and Elder, 1997; Marshall et al., 2001; Jackson et al., 2005). Among the 63 genes (59 protein coding genes and four pseudogenes) located within the EDC, null mutations in the gene encoding profilaggrin (*FLG*), an SFTP gene, was shown to be the major risk factor for AD (Morar et al., 2007).

The first suggestion of the involvement of filaggrin in barrier maintenance was reported by Sybert et al. (Sybert et al., 1985) where reduction of its expression was correlated with ichthyosis vulgaris (IV); follow-up studies confirmed reduction or loss of filaggrin expression with epidermal barrier dysfunction (Fleckman et al., 1987; Peña Penabad et al., 1998). A breakthrough came with the discovery that loss of function *FLG* mutations (R501X and 2282del4) are highly prevalent in the IV (Smith et al., 2006) and AD patients (Palmer et al., 2006); this was replicated on different genetic backgrounds and ethnicities (Marenholz et al., 2006; Ruether et al., 2006; Sandilands et al., 2006; Weidinger et al., 2006; Barker et al., 2007; Morar et al., 2007; Nomura et al., 2007; Enomoto et al., 2008; Rodríguez et al., 2008; Nemoto-Hasebe et al., 2009; Osawa et al., 2010; Cheng et al., 2012; Pigors et al., 2018; Handa et al., 2019; Koseki et al., 2019; Jurakic Toncic et al., 2020; Smieszek et al., 2020), with hundreds of mutations now identified (Karczewski et al., 2020). Importantly, while filaggrin expression is almost entirely restricted to the epidermis, *FLG* mutations have been also shown to be linked to additional manifestations of atopic march and allergy, including food (Brown et al., 2011) and contact allergies (Novak et al., 2008), asthma (van den Oord and Sheikh, 2009; Rodríguez et al., 2009), allergic rhinitis (van den Oord and Sheikh, 2009) and eosinophilic esophagitis (Sherrill and Blanchard, 2014).

Composed of approximately 4,061 amino acids (aa), profilaggrin is the largest protein of the SFTP family; the protein is structurally complex and composed of 10–12 filaggrin monomer repeats flanked with truncated filaggrin repeats (Gan et al., 1990) and a S100 domain at the N-terminus (Kypriotou et al., 2012). Unlike any other SFTP, profilaggrin contains a “bipartite” nuclear localization signal (Lu et al., 2021) next to the S100 calcium-binding domain, an indicator of its nuclear function (Presland et al., 1992; Aho et al., 2012). Upon expression in keratinocytes, profilaggrin is phosphorylated and stored within keratohyalin granules (KHGs), mainly present in the *stratum granulosum* layer (Resing et al., 2002), from where it is released by an AKT1-dependent, actin scaffold-driven mechanism that we have recently described (Gutowska-Owsiak et al., 2018). It is speculated that dephosphorylation makes the protein accessible to the pro-protein convertase-mediated cleavage (Resing et al., 1993). The cleaved N-terminal domain translocates into the nucleus, where it is involved in denucleation (Ishida-Yamamoto et al., 1998; Pearton et al., 2002; Yamamoto-Tanaka et al., 2014) and control of epidermal homeostasis (Aho et al., 2012; Naeem et al., 2015); the remaining part is cleaved by SASPase (Matsui et al., 2011) and KLK5 (Sakabe et al., 2013) proteolytic enzymes. Monomeric filaggrin promotes aggregation and collapse of keratin intermediate filaments (IFs), resulting in the formation of more squamous flattened cells (Steinert et al., 1981; Candi et al., 2005). In parallel, further events occur, such as the conversion of arginine (Arg) residues to citrulline (Nachat et al., 2005) and covalent cross-linking of monomers by transglutaminase, which leads to stabilization of the cornified cell envelope (Takahashi et al., 1996). Finally, the crosslinked filaggrin undergoes extensive proteolytic cleavage by caspase-14 (Denecker et al., 2007), calpain-1 (Yamazaki et al., 1997), bleomycin hydrolase (Kamata et al., 2009), elastase-2 (Bonnart et al., 2010), matripase (List et al., 2003), prostatin (Leyvraz et al., 2005) and other proteases, resulting in a pool of hygroscopic aa and derivatives, i.e., urocanic acid (UCA), a derivative of histidine, highly abundant in the protein, and pyrrolidone carboxylic acid (PCA), a glutamine derivative. These constitute the majority of the so-called “natural moisturizing factor” (NMF) (Rawlings and Harding, 2004), contributing to *stratum corneum* (SC) hydration, as well as acidic pH, with antimicrobial action (Miajlovic et al., 2010). In addition, trans-UCA may protect cells in deeper layers from UVB-mediated mutagenesis by absorbing UVB (Tabachnick, 1957). *FLG* mutations significantly reduce the amount of NMF in SC compared to the healthy control (Kezic et al., 2011).

The ubiquitin-proteasome system (UPS) is a major proteolytic pathway that removes damaged and unwanted proteins. The selective turnover is initiated by a covalent attachment of a small ubiquitin (Ub) protein, mainly to the internal lysine (Lys) residues, which is mediated by an enzymatic cascade orchestrated by E1, E2, and E3 enzymes (Hershko and Ciechanover, 1992; Komander and Rape, 2012). However, a single ubiquitination event is usually insufficient to target a protein for degradation; several kinds of polyubiquitin chains are formed between the Lys residues of the Ub subunits and these have specific functions (Hershko and Ciechanover, 2003; Husnjak and Dikic, 2012; Oh et al., 2018). The Lys11 and Lys48 linkages, and their combination drive proteasomal degradation (Chau et al., 1989; Jin et al., 2008; Meyer and Rape, 2014), whereas Lys6 and Lys63 linkages mediate the processes of autophagy (Ordureau et al., 2014), endocytosis-exocytosis (Lauwers et al., 2009)^,^ or lysosomal degradation (Duncan et al., 2006). Interestingly, ubiquitination can also occur on the free amino group of the N-terminus of a protein, as well as on serine (Ser), cysteine (Cys), and threonine (Thr) residues, and can lead to proteasomal turnover of the modified proteins (Kravtsova-Ivantsiv and Ciechanover, 2012; McClellan et al., 2019).

E3 ligases, which transfer Ub onto a substrate protein, mainly recognize substrates through their short linear motif called the primary degron, which may be located at the unstructured ends of the proteins, inducing protein elimination via the N- or C-degron pathways or internally. In addition, proteolytic cleavage may lead to the emergence of a degron motif within the novel N- or C-terminus, subsequently initiating degradation of the cleavage products (Guharoy et al., 2016; Varshavsky, 2019; Guharoy et al., 2022). Moreover, post-translational modifications (PTMs) can also modulate the recognition of the primary degrons by E3 ligases (van Roey et al., 2013; Guharoy et al., 2016; Lucas and Ciulli, 2017; Millar et al., 2019; Varshavsky, 2019; Chen and Kashina, 2021; Guharoy et al., 2022). Following interaction with a degron, E3 mediates ubiquitination in the proximal position(s) (secondary degron), often found close to the intrinsically disordered region (IDR) (tertiary degron), which triggers substrate breakdown by successful activation of the proteasome. Mutations affecting degron components can enhance protein stability and disrupt cellular proteostasis, leading to disease (Tokheim et al., 2021; Guharoy et al., 2022; Kampmeyer et al., 2022). Accordingly, adequately controlled profilaggrin turnover is likely crucial for SC functionality since its reduced expression or instability confers vulnerability and impacts several epidermal functions, leading to pathological conditions (Scott et al., 1982; Resing et al., 1984; Moosbrugger-Martinz et al., 2022). However, despite the central role of profilaggrin and filaggrin in skin barrier function, little is known about the regulation of their degradation, the proteolytic mechanisms involved, and the influence of *FLG* mutations on their turnover and stability.

In this study, we investigated proteasome inhibition’s effect on the intracellular profilaggrin level and its processing products. We also studied the entire profilaggrin sequence for the presence of degrons and examined possible ubiquitination sites throughout. Furthermore, we analysed the effect of recurrent pathogenic and rare family-specific frameshift and nonsense *FLG* mutations on the introduction of possible ubiquitination sites, the appearance of novel degrons, and protein stability.

## Materials and methods

### Keratinocyte culture

Human keratinocyte cell line N/TERT1 (Dickson et al., 2000; Smits et al., 2017), a kind gift from J. Rheinwald laboratory (Harvard Medical School, Boston, United States), was cultured in T75 flask or T150 flask in Keratinocyte serum free medium (K-SFM), with L-glutamine, without CaCl_2_ (Gibco™, Thermo Fisher Scientific, Cat#10725018) and supplemented with 0.2 ng epidermal growth factor (EGF) per ml, 25 μg bovine pituitary extract (BPE) per ml (Gibco™, Thermo Fisher Scientific, Cat#13028014), 100 unit penicillin per ml (Sigma Aldrich, Cat#P4333), 100 μg streptomycin per ml (Sigma Aldrich, Cat#P4333) and 0.4 mM CaCl_2_ (VVR, Cat#97062822) up to 40% confluency. Cells were plated in 6-well plates at 300,000 cells per well in K-SFM complete medium and incubated at 37°C, 5% CO_2_ for 48 h.

### Calcium switch, proteasome inhibition and deubiquitinase inhibition

After 48 h the medium was replaced with DFK medium composed of 1:1 of calcium-free Dulbecco’s Modified Eagle Medium (DMEM) containing 4.5 mg D-glucose per ml, and Ham’s F12 nutrient mix (Gibco™, Thermo Fisher Scientific, Cat#11765054), supplemented with 0.2 ng EGF per ml, 25 μg BPE per ml, 2 mM L-glutamate (Sigma Aldrich, Cat#G7513-100 ML), 100 unit penicillin, 100 μg streptomycin per ml and 1.5 mM CaCl_2_ (to trigger calcium induced differentiation; i.e., “calcium switch”). Upon differentiation (>24 h at 1.5 mM CaCl_2_) mediated by calcium switch, keratinocytes were treated with 10 μM MG132 (Sigma Aldrich, Cat#474790); a potent, reversible proteasome inhibitor or 10 µM PR-619 (Sigma Aldrich, Cat#SML0430-1 MG), a broad range deubiquitinase inhibitor; dissolved in DMSO for 2 h, 4 h, 8 h and 16 h. Protein extraction was performed after 48 h of calcium switch.

### Western blot

The cells were lysed with RIPA buffer (Cell Signalling Technology, Cat#9806) supplemented with cOmplete™ protease inhibitor cocktail (Roche, Cat#11836170001). Cell lysates were harvested by centrifugation at 14,000 g for 15 min at 4°C and denatured with 4X Bolt™ LDS sample Buffer (Novex^®^, Life technology™, Cat#B0007) at 70°C for 10 min. Samples were run on 4%–12% polyacrylamide gradient gel (Invitrogen™, Thermo Fisher Scientific, Cat#NP0321BOX). Protein transfer was carried out onto a nitrocellulose membrane {[iBlot™ 2 Transfer Stacks (Invitrogen™, Thermo Fisher Scientific, Cat#IB23001)]} on the iBlot™ 2 dry blotting system (Invitrogen™, Thermo Fisher Scientific, Cat#IB21001). Membranes were blocked with 5% fat-free milk in phosphate buffer saline (PBS), followed by overnight incubation with 1:200 anti-filaggrin monoclonal antibody (FLG01; raised to recombinant filaggrin) (Invitrogen™, Thermo Fisher Scientific, Cat#MA513440; FLG01 monoclonal antibody was used in this study and was validated for its specificity (Supplementary Figure E1 or 1:5,000 dilution of anti-GAPDH antibody (6C5) (Santa Cruz Biotechnology, Cat#SC32233) at 4°C. Secondary antibody incubation was carried out with 1:25,000 PBS diluted IRDye^®^800CW donkey anti-mouse (LI-COR^®^, Cat#92632312) and imaged with Odyssey^®^ CLx Imaging System (LI-COR^®^ Biosciences). Membranes were stripped with the Restore™ fluorescence western blot stripping buffer (Thermo Scientific™, Cat#62300) according to the manufacturers’ instruction and stained with 1:1,000 dilution of anti-ubiquitin antibody (Santa Cruz Biotechnology, Cat#sc-8017) and detected as of the procedure described above. Acquired images were analysed with ImageJ (Schneider et al., 2012) (v.1.53f51) for protein quantification as a means of protein band intensity. The intensity of filaggrin protein bands was expressed as a band intensity ratio compared to the GAPDH band. One-way or two-way ANOVA (Tukey’s multiple comparison test) was performed with GraphPad Prism (v.9.4.1) to compare the variance between different treatment groups.

### *FLG* sequence and AD-relevant mutations

The amino acid sequence of profilaggrin (UniProt ID P20930; NP_002007.1; herein referred as wild-type) was examined for the presence of Lys, Ser, Thr, and Cys residues as possible ubiquitination sites on individual domains. To examine if frameshift mutations alter the number of Lys, Ser, Thr, and Cys residues, all the mutations of *FLG* identified to date were retrieved from the Genome Aggregation Database (gnomAD, v2.1.1.) (accessed on 9.02.2022) (Karczewski et al., 2020). In addition, we cross-checked for additional frameshift mutations in the Gene4Denove (accessed 26.08.2022) (Zhao et al., 2020) and denovo-db v.1.6.1 (accessed 28.08.2022) (Seattle, 2022) databases. The mutations were introduced to the wild-type nucleotide sequence (NM_002016.2) and translated using the EMBL-EBI nucleotide sequence translation tool EMBOSS transeq (Madeira et al., 2022). Recurrent pathogenic mutations located within the coding sequence NM_002016.2: c.1 to c12183 and rare family-specific mutations located within the coding sequence NM_002016.2: c. 1 to c.5700 were used for the subsequent analyses.

### Screening for degron motifs and stability of protein N- and C-termini

In all cases, we used our recently released tool DEGRONOPEDIA (Szulc et al., 2022) (all presented data in this work comply with the DEGRONOPEDIA’s version from 19.09.2022) to screen for the known degron motifs and post-translational modifications (PTMs), simulate proteolysis, calculate the Gravy hydrophobicity index (GHI) of terminal 15 residues (Kyte and Doolittle, 1982) and report experimental or predicted Protein Stability Index (PSI) values (Koren et al., 2018; Timms et al., 2019) for 23 residues at each of the N- and C-termini. Additionally, the DEGRONOPEDIA server provided experimentally validated E3s interacting with profilaggrin based on the BioGRID (Biological General Repository for Interaction Datasets) (Oughtred et al., 2021) and IntAct (Orchard et al., 2014) databases. Since sequences shorter than 50 aa are unsuitable input for the DEGRONOPEDIA, we excluded them from our *FLG* mutant variants analysis.

### Analysis of amino acid sequence conservation

To investigate the conservation of profilaggrin ubiquitin-conjugating amino acids and the degron motifs within SFTPs and S100 proteins, we utilized EMBL-EMBI multiple sequence alignment tool Clustal Omega (Madeira et al., 2022).

## Results

### Inhibition of the proteasome and deubiquitinases results in the accumulation of profilaggrin and its processed products of high molecular weight

**FIGURE 1:**
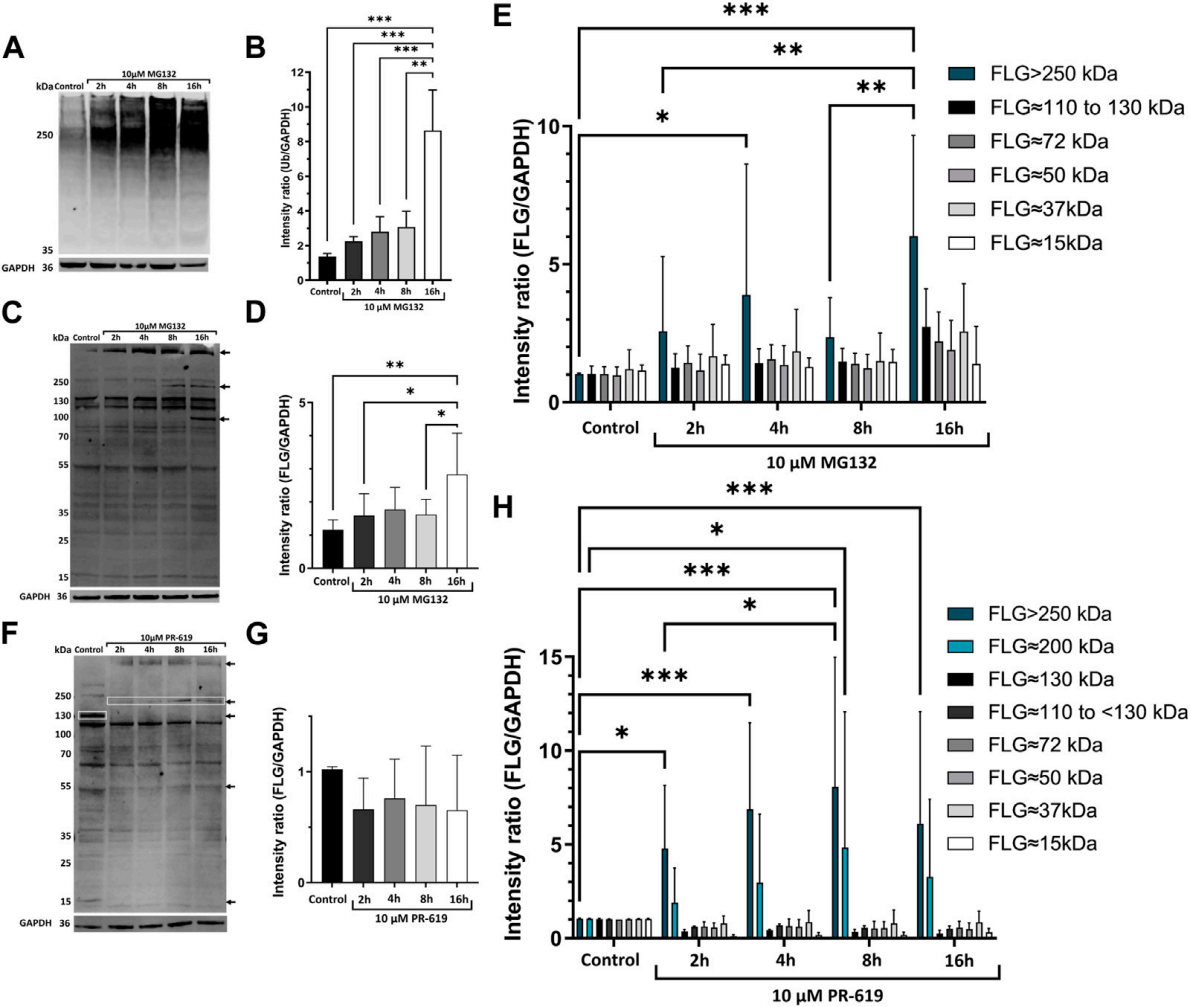
UPS is involved in degradation of profilaggrin. **(A)** Western blot with anti-ubiquitin in the keratinocytes upon treatment with proteasome inhibitor MG132; **(B)** Intensity ratio of ubiquitinated protein bands after proteasome inhibition for 2 h, 4 h, 8 h and 16 h; one-way ANOVA followed with Tukey’s multiple comparison test. **(C)** Western blot with anti-filaggrin in keratinocytes upon 2 h, 4 h, 8 h and 16 h treatment of proteasome inhibitor MG132, arrows indicate accumulation of undegraded profilaggrin in proteasome inhibition sample. **(D)** Intensity ratio of all filaggrin bands detected in different timepoint treatment of proteasome inhibitor MG132; one-way ANOVA followed by Tukey’s multiple comparison test; **(E)** Intensity ratio of different FLG bands upon MG132 treatment; two-way ANOVA followed by Šidák’s multiple comparison test; **(F)** Western blot with anti-filaggrin in keratinocytes upon 2 h, 4 h, 8 h and 16 h treatment of deubiquitinase inhibitor PR-619, Top two arrows and top box indicates accumulation of higher molecular weight filaggrin bands in the deubiquitinase inhibited samples whereas, deubiquitinase inhibition leads to depletion or disappearance of filaggrin bands of approximately 130, 50 and 15 kDa pointed with bottom box and arrows. **(G)** Intensity ratio of all filaggrin bands detected in different timepoint treatment of deubiquitinase inhibitor PR-619; one-way ANOVA followed by Tukey’s multiple comparison test; **(H)** Intensity ratio of different FLG bands upon PR-619 treatment; two-way ANOVA followed by Šidák’s multiple comparison test; error bar stands for + SD and *p*-value < 0.001(***); *p*-value ≤0.002(**); *p*-value ≤ 0.033(*); (*n* = 4).

To determine if the intracellular levels of profilaggrin could be, at least partially, controlled by the proteasome-mediated turnover of the nascent protein, we used 2D-grown N/TERT-1 (Dickson et al., 2000; Smits et al., 2017) keratinocytes as our model because the expression of FLG mRNA and protein in these immortalized cells shown to be similar to that of primary human keratinocytes consisting WT FLG (Smits et al., 2017). Treatment with the proteasome inhibitor MG132 had no effect on cell morphology compared to the control (solvent control DMSO), with shorter treatment times and only a slight reduction in culture confluence (Supplementary Figure E1). In contrast, with the late time point (16 h), we noticed a reduction in cell viability and cells losing contact with the substratum. Proteasome inhibition increased the accumulation of ubiquitinated protein as expected (Figure 1A) and this increase was gradual in our time-point experiment, with the highest intensity measured after 16 h of 10 µM MG132 incubation (Figure 1B). In line with our expectations, proteasome inhibition altered the content of profilaggrin and profilaggrin-processed products; specifically, we observed an increase in the overall intensity of profilaggrin and filaggrin-relevant bands (adjusted to GAPDH) in the cells treated with MG132 (Figures 1C, D). This change was observed as an increase in high molecular weight products (Figure 1E) and was apparent after the overnight treatment. Specifically, we noticed an increase in the adjusted intensity of the bands corresponding to the products of 110–130 kDa (likely containing 3-4x filaggrin monomer repeats) and those of over 250 kDa in weight (likely including profilaggrin and a very high molecular weight processed product) (arrows in Figures 1C, E). This increase was pronounced despite harvesting fewer cells due to the reduction in cell viability at the 16 h time point. These results indicate that the proteasome is involved in profilaggrin turnover, and its inhibition results in the stabilization of profilaggrin and high molecular weight derivatives.

To investigate the possibility of profilagrin undergoing ubiquitination, we utilized a pan-DUB inhibitor, PR-619, as a proof of concept. Our results indicate that the inhibitor had an impact on the ubiquitination of profilagrin. Specifically, we observed accumulation of higher molecular weight products, presumably ubiquitinated (Figure 1F), although the total content of all the profilaggrin/filaggrin-relevant products remained the same in comparison to the solvent control (Figure 1G). Intensity of the bands >250 kDa in size was significantly increased at all time points of the deubiquitinase inhibition, while intensity of the ∼200 kDa band was significantly increased at 8 h time point. At the same time, we could see disappearance of the lower molecular weight bands upon PR-619 treatment (Figures 1F, H). These data, together with the effect of proteasome inhibition, highly suggest that profilaggrin is subjected to significant ubiquitination, which is also likely to be responsible for its turnover.

### The profilaggrin sequence contains multiple potential degrons

**FIGURE 2:**
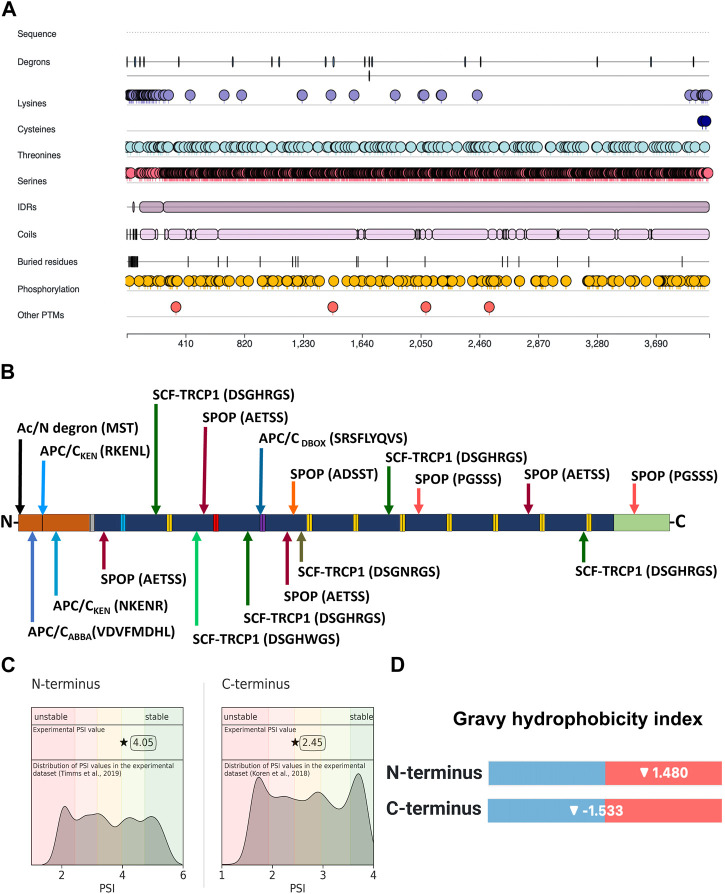
Location of degron motifs, ubiquitination-prone residues, and terminal stability of profilaggrin. **(A)** Illustration showing location of degron, ubiquitin-conjugating amino acid residues, intrinsic disorder region, phosphorylation sites and other post-translational modification sites in the profilaggrin protein. **(B)** Location of degron motif sequence and degron type in the profilaggrin wild-type sequence (legend as of Figure 3); **(C)** protein stability index of the N- and C- terminus of the profilaggrin; **(D)** Gravy hydrophobicity index of profilaggrin N- and C terminus.

Having confirmed the proteasome involvement in the turnover of profilaggrin, we next set out to determine degron components in the protein sequence using our DEGRONOPEDIA web server, which enables comprehensive annotation of degron motifs and potentially related PTMs, with a particular focus on ubiquitination and phosphorylation. We noted that native profilaggrin has 18 primary degrons, relatively evenly distributed within the sequence (Figures 2A, B; all found primary degron motifs are summarized in Table 1). Specifically, the N-terminal sequence of profilaggrin contains potential acetylation sites that could direct it to the Ac/N-end rule pathway under specific conditions such as proteotoxic stress or immune response; we also recorded other degron motifs within the profilaggrin sequence, i.e., the destruction box (DBOX), KEN box, ABBA (DiFiore et al., 2015; Davey and Morgan, 2016) and SCF^β−TRCP^ (Skp1-cullin 1-F-box with β-transducin repeat-containing protein acting as its substrate receptor) motifs. In addition, profilaggrin has eight sequences corresponding to the consensus of motifs recognized by SPOP (Speckle-type POZ—pox virus and zinc finger protein), an adaptor protein for cullin 3 (CRL3)-based E3 ligases (Zhuang et al., 2009). SPOPs typically operate in the nucleus, playing a critical role in regulating apoptosis and cell proliferation. One of these, the ADSST motif located in the 4th filaggrin repeat unit of the FLG (1,689–1,693 aa), is an experimentally confirmed degron through which SPOP controls the level of hybrid protein BCR-ABL1 that governs the expression of several differentiation-related genes (Quintás-Cardama and Cortes, 2009; Liu et al., 2021). In addition, profilaggrin undergoes phosphorylation at this motif, at Ser 1,691, which could modulate SPOP binding to this degron. Molecular interaction databases BioGRID and IntAct also report on the probable binding of profilaggrin by other receptors of the cullin E3 ligases: FBXW7 (Xu et al., 2021), DTL (Huttlin et al., 2021), and VHL (Ewing et al., 2007).

**TABLE 1:** List of degron motif, type and location in the profilaggrin protein sequence.

Degron motif	Sequence	Indices	Degron type	Localization	Additional information	Secondary structure	Mean relative solvent accesibility	Mean pLLDT
M{0,1}[AST]x	MST	1–3	Ac/N degron	N-terminus	This motif is recognized by *S. cerevisiae* Doa10 and its mammalian counterpart Teb4, and also Not4, the E3 subunit of Ccr4-Not (Varshavsky, 2019)	--H	0.69	91.4
[FIVL]x[ILMVP][FHY]x[DE]x{0,3}[DEST]	VDVFMDHL	54–62	APC/C (ABBA)	Internal		HHHHHHTT-	0.28	80.82
xKENx	RKENL	90–94	APC/C (KEN)	Internal		HHHTS	0.68	61.62
xKENx	NKENR	118–122	APC/C (KEN)	Internal		-----	0.74	33.55
[AVP]x[ST][ST][ST]	AETSS	361–365	SPOP	Internal		-----	0.86	34.65
D(S)Gx{2,3}([ST])	DSGHRGS	736–742	SCF-TRCP1	Internal		-------	0.87	30.77
D(S)Gx{2,3}([ST])	DSGHWGS	1,060–1,066	SCF-TRCP1	Internal		-------	0.83	33.22
[AVP]x[ST][ST][ST]	AETSS	1,010–1,014	SPOP	Internal		-----	0.86	34.84
D(S)Gx{2,3}([ST])	DSGHRGS	1,384–1,390	SCF-TRCP1	Internal		-------	0.88	41.18
xRxxLxx[LIVM]x	SRSFLYQVS	1,438–1,446	APC/C (DBOX)	Internal		---------	0.85	33.96
[AVP]x[ST][ST][ST]	AETSS	1,658–1,662	SPOP	Internal		-----	0.84	39.03
ADSST	ADSST	1,689–1,693	SPOP	Internal	Is an experimentally validated degron for human death domain-associated protein 6 (DAXX) recognized by BCR E3 ligase (Guharoy et al., 2016)	-----	0.89	40.22
[AVP]x[ST][ST][ST]								
D(S)Gx{2,3}([ST])	DSGNRGS	1708–1714	SCF-TRCP1	Internal		-------	0.9	39.66
D(S)Gx{2,3}([ST])	DSGHRGS	2,357–2,363	SCF-TRCP1	Internal		-------	0.83	33.6
[AVP]x[ST][ST][ST]	PGSSS	2,466–2,470	SPOP	Internal		-----	0.95	32.85
[AVP]x[ST][ST][ST]	AETSS	3,279–3,283	SPOP	Internal		-----	0.86	38.49
D(S)Gx{2,3}([ST])	DSGHRGS	3,653–3,659	SCF-TRCP1	Internal		----S--	0.82	37.03
[AVP]x[ST][ST][ST]	PHSSS	3,950–3,954	SPOP	Internal		-----	0.76	42.85

### Profilaggrin shows differential stability at the C- and N-termini

Based on the experimentally measured Protein Stability Index (PSI), our tool showed that the C-terminus of profilaggrin exhibits a PSI value of 2.45 (the median C-terminal PSI in human proteome is 2.72), whereas the N-terminus is more stable with a PSI value of 4.05 (the reported experimental PSI value is for profilaggrin N-terminal sequence with initiator methionine (Met) cleaved, there is no data on the corresponding variant with initiator Met present; the median N-terminal PSI in human proteome for termini where initiator methionine (Met) undergoes cleavage is 3.49) (Figure 2C). In contrast, the N-terminus has a positive Gravy hydrophobicity index (GHI) value (Kyte and Doolittle, 1982) (Figure 2D), and hydrophobic sequences often determine the specificity for recognition by chaperones and protein quality control E3s (Kats et al., 2018; Stefanovic-Barrett et al., 2018; Hickey et al., 2021; Culver et al., 2022).

### The profilaggrin sequence contains multiple potential ubiquitination sites

**FIGURE 3:**
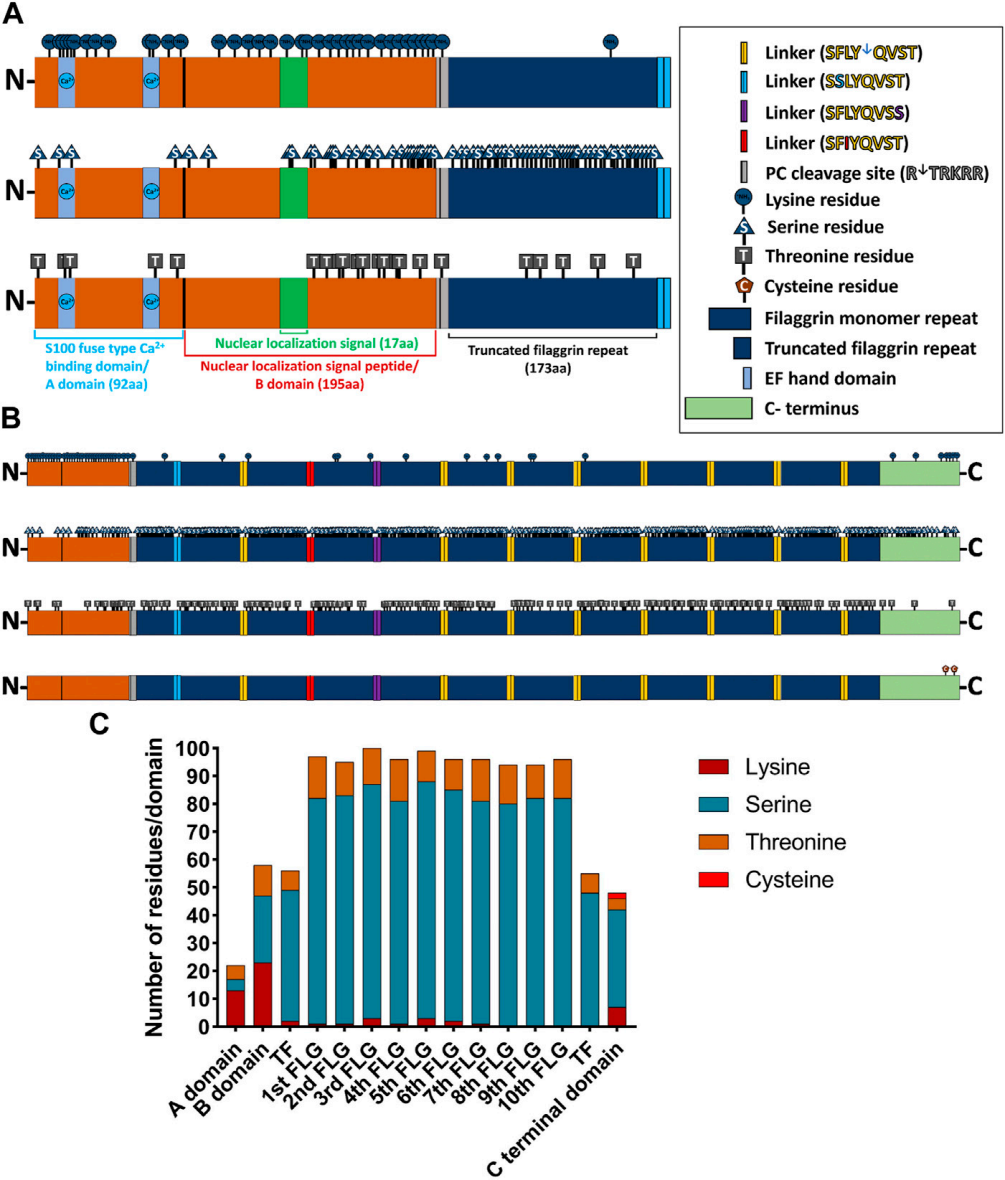
Distribution of ubiquitin (Ub)-conjugating residues in the profilaggrin wildtype sequence. **(A)** Illustration of N terminus of FLG. The N terminal of profilaggrin consists of a S100 fused type Ca^++^ Binding domain (92 aa) also known as A domain followed by nuclear localization signal peptide (195 aa) also known as B domain and a truncated filaggrin repeat (173 aa). Truncated filaggrin repeat is flanked with a PC cleavage site which links it with B domain and SASPase cleavage site which links it with rest of the filaggrin. Like all the other S100 domain filaggrin S100 domain consists of the Ca^++^ binding domain followed by a unique nuclear localization signal. **(B)** Illustration of the wild-type profilaggrin protein. **(C)** Number of Ub-conjugating amino acids in different domains of wild type profilaggrin.

We identified numerous Lys residues after examining the profilaggrin sequences for the secondary degrons—potential ubiquitination sites. Intriguingly, these appear to be dispersed unevenly throughout the profilaggrin sequence, with the majority accumulating either in the N-terminal domain (Figure 3A) or at the C-terminus (Figure 3B). In contrast, Lys residues are comparatively rare within the filaggrin monomeric repeats: 1st, 2nd 4th and 7th repeat each contain only one Lys residue; 6th repeat contains two and 3rd and 5th repeat harbor three Lys residues (Figures 3B, C). Apart from Lys, ubiquitin conjugation can also occur at the Ser, Thr, and Cys residues (the non-canonical ubiquitination events). In contrast to the Lys residues, which are localized mainly on the terminal ends, the majority of Ser and Thr residues are located within the filaggrin repeat units (Figures 3A–C); i.a., each filaggrin repeat unit harbors 80 to 85 Ser and 10 to 15 Thr residues. The N-terminal truncated filaggrin contains 47 Ser and 7 Thr, and C-terminal truncated filaggrin contains 48 Ser and 7 Thr residues (Figures 3A–C). Interestingly, profilaggrin contains only two Cys residues, both located at the C-terminus (Figure 3B). Altogether, despite the enrichment in the canonical signals (Lys) at the N- and C-terminus of the sequence, which could be potentially ubiquitinated, filaggrin monomer repeats contain almost five times more potential non-canonical ubiquitination sites. The distribution of Lys residues mainly in the vicinity of the profilaggrin ends and the presence of a vast inner region devoid of those (2,460–3,800 aa) may suggest that profilaggrin stability is regulated by N- and C-terminus-dependent pathways.

### Protease action generates filaggrin monomers with reduced degron potential at their N-termini

**FIGURE 4:**
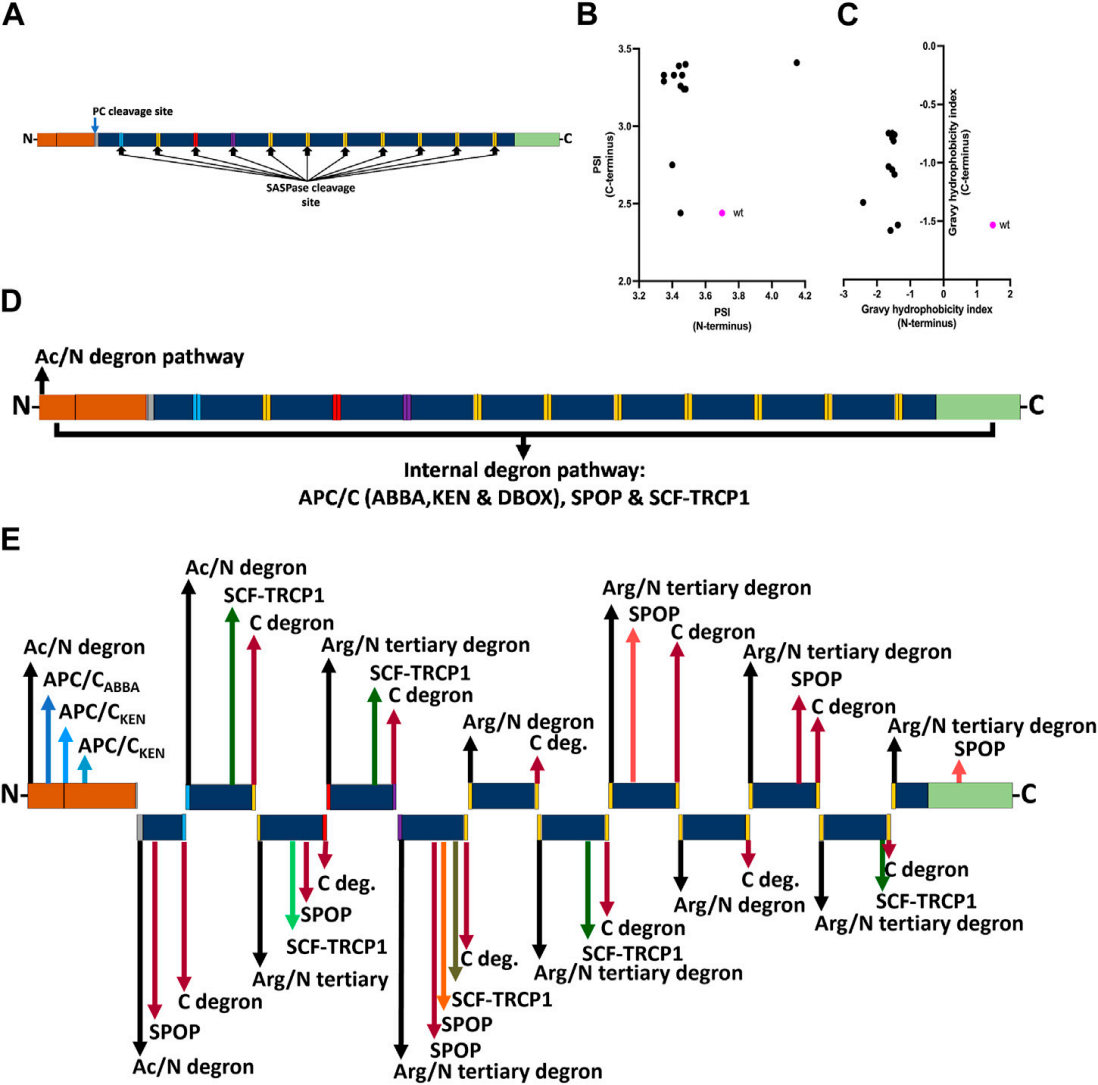
Effect of profilaggrin processing on the stability and degron content. **(A)** PC and SASPase clevage sites in the profilaggrin sequence. **(B)** terminal protein stability index of newly generated SASPase cleaved FLG products in comparison to the wild-type FLG terminus. **(C)** terminal Gravy hydrophobicity index of newly generated SASPase cleaved FLG products in comparison to the wild-type FLG terminus. **(D)** Proposed degradation routes of wild-type profilaggrin. **(E)** Proposed degradation routes of PC- and SASPase-cleaved profilaggrin products (legends as of Figure 3).

Profilaggrin is cleaved into 10–12 filaggrin monomers by several endoproteases, including the skin-specific retroviral aspartic protease (SASPase) and enzymes of the precursor converting enzyme (PC) family of serine proteases. Using DEGRONOPEDIA, we simulated cleavage of the profilaggrin sequence by PC and SASPase (Figure 4A) to analyse the stability and hydrophobicity of the resulting products as well as to uncover degron motifs in the newly formed termini that may have physiological significance. PC processing results in an N-terminus with residues that can be acetylated and targeted via the Ac/N-degron pathway (Supplementary Table E1). On the other hand, upon processing by SASPase, most of the resulting filaggrin monomer repeats feature an N-terminus containing positively charged (His, Lys, and Arg) and large hydrophobic residues [tryptophane (Trp), isoleucine (Ile), phenylalanine (Phe), leucine (Leu), and tyrosine (Tyr)], which can be recognized by UBR1, UBR2, UBR4, and UER5 E3 ligases or non-E3 autophagy receptor p62/SQSTM, and targeted for degradation via the Arg/N-degron pathway (Supplementary Table E2) (Dissmeyer et al., 2018; Yoo et al., 2018; Varshavsky, 2019). Despite the lower PSI value (indicating lower stability) of the N-terminus of these monomer repeats (Figure 4B), their hydrophobicity index (one of the components used to predict PSI) is also lower overall than that of the entire profilaggrin (Figure 4C). This may imply that their potential turnover based on various protein quality control E3 ligases, such as the C-terminus of HSC70-interacting protein (CHIP) or March6, which rely on hydrophobic degrons to mediate the destruction of proteins, will be impeded (Stefanovic-Barrett et al., 2018; Wang et al., 2020). Therefore, while the predicted degradation routes for the intact wild-type profilaggrin are shown in Figure 4D, we speculate that the monomer repeats generated via PC/SASP cleavage will be regulated via internal degrons or C-degron pathways (Figure 4E). Accordingly, the relatively low hydrophobicity of cleavage products at their C-termini can facilitate their interaction with cullin-RING ligases, which preferentially act upon non-hydrophobic C-terminal degrons (Hickey et al., 2021).

Degrons and ubiquitination sites are conserved within the S100 fused-type protein (SFTP) family, including filaggrin. We examined whether the SFTP have similar degron motifs and residue pattern of Lys, Ser, Thr, and Cys. Enrichment of Lys residues at the N-termini is conserved within the S100 domain (aa 1 to 100; Supplementary Figure E3). Additionally, we analysed the conservation of Lys residues among all S100 calcium-binding proteins and the S100 domain of SFTP (Supplementary Figure E4). Interestingly, the filaggrin S100 domain contains the highest number of Lys (14 residues) compared to the S100 proteins and the S100 domains of the SFTP (Supplementary Figure E5; Supplementary Table E5). In contrast, Ser residues are well conserved between filaggrin, hornerin, and filaggrin 2 in the filaggrin repeat units. Degrons also exhibit a degree of conservation within the family (Supplementary Table E6); e.g., cornulin, trichohyalin, and trichohyalin-like protein 1 contain DBOX motifs that can be recognized by the APC/C E3 complex. Trichohyalin, in addition, contains the KEN motif, which can also interact with APC/C. All the SFTPs have N-termini that can function through the Ac/N-degron pathway. Moreover, hornerin contains a sequence that can bind to E3 SPOP, and filaggrin-2 contains a degron recognized by the SCF-TRCP1. These properties imply that multiple proteins in the SFTP family may be subject to degradation by similar UPS pathways.

### Clinically relevant *FLG* null mutations affect the number of predicted ubiquitination sites

Given the likely importance of ubiquitination affecting (pro) filaggrin turnover in the skin, we also investigated if known genetic predisposition for AD, i.e., *FLG* null mutations, may impact this process. To this end, we searched for any additions or removals of Lys, Ser, Thr, and Cys residues from the protein sequence resulting from *FLG* frameshift mutations and compared the number of those residues in the frameshift product with the same length span in the wild-type sequence. We retrieved all the mutations of *FLG* registered to date in the Genome Aggregation Database (gnomAD, v2.1.1.). We found that out of 23 recurrent and pathogenic frameshifts analysed, 13 (57%) generated products with a greater number of Lys residues compared to the corresponding wild-type amino acid span (Supplementary Figure E5). The highest Lys residue enrichment of eight residues was encountered in p.Ser1235HisfsTer211. Overall, these mutations introduce an additional one to eight Lys residues in the frameshift product. Lys residues remain consistent for the remaining 10 pathogenic frameshift mutations. Six of those (p.His3951ProfsTer4, p.Gln2423ValfsTer2, p.Ser2317Ter, p.Ser417ValfsTer2 or c.1248dupG, p.Gly221GlufsTer3 and p.Asn186LysfsTer4) terminate immediately, yielding products shorter than three amino acids in length, without the introduction of any Lys residues. Similarly, frameshift mutations p.Ser1171GlnfsTer15 or c.3510delG, p.Gly1109GlufsTer13 or c.3321delA, p.Gln1084ValfsTer21 or c.3250_3251delCA and p.Asp433HisfsTer43 or c.1297_1298delGA do not introduce any Lys residues despite generating a moderate length of frameshift products (Supplementary Figure E6; Supplementary Table E4).

**FIGURE 5:**
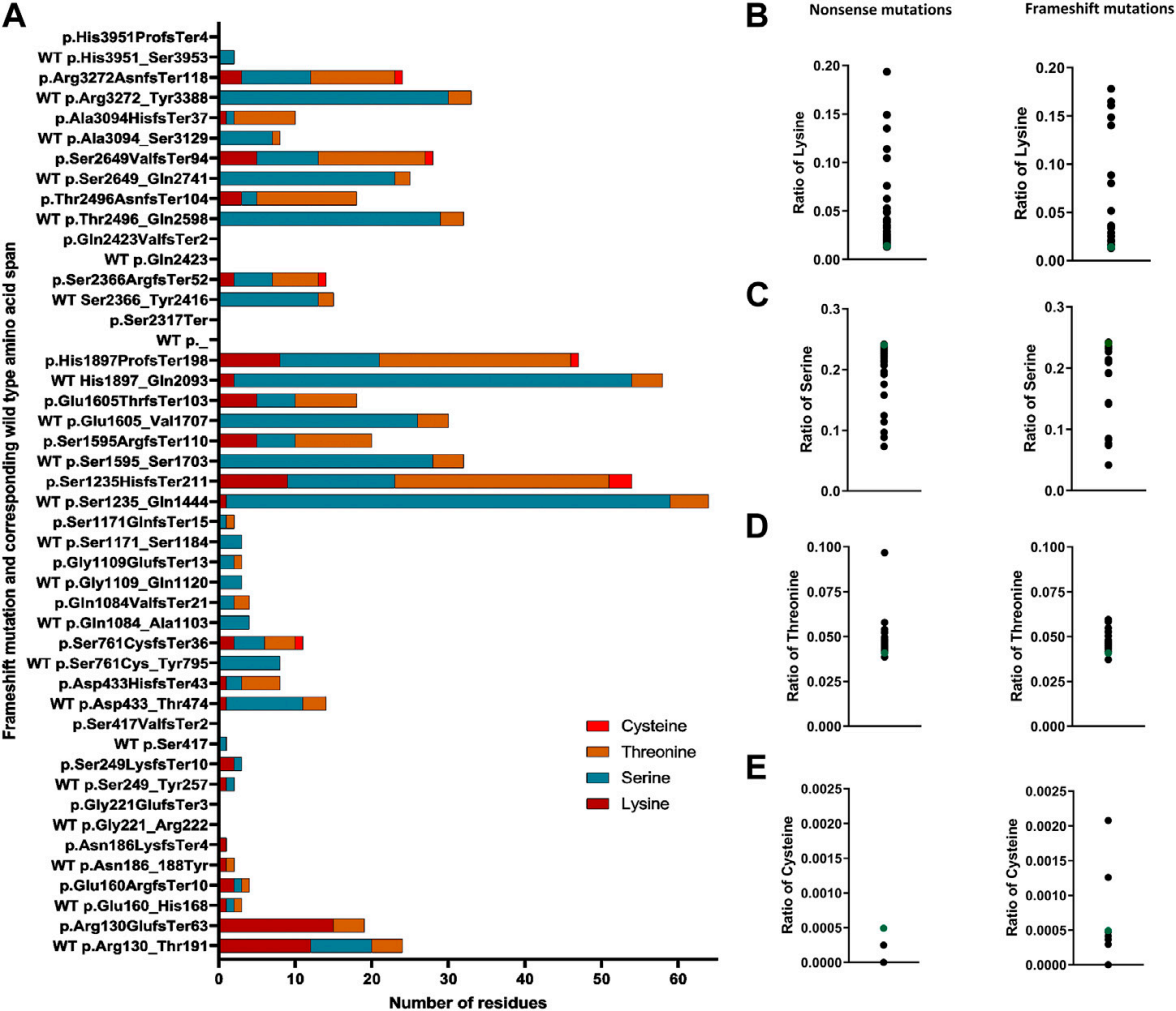
Altered content of ubiquitin-conjugating residues in FLG mutation products. **(A)** Number of ubiquitin-conjugating residues in the frameshift products in comparison to the corresponding wild-type same length span. **(B)** Ratio of lysine in the nonsense and frameshift mutant protein. **(C)** Ratio of serine (Ser) in the nonsense and frameshift mutant protein. **(D)** Ratio of threonine (Thr) in the nonsense and frameshift mutant protein. **(E)** Ratio of cysteine in the nonsense and frameshift mutant protein.

A similar trend was observed for Thr and Cys residues—out of the 23 mutations, 14 (61%) generate a frameshift product with an increased number of Thr residues (1–23 additional Thr residues). Only one mutation (p.Asn186LysfsTer4 or c.557dupA) reduces the number of Thr (reduction of one residue) in the frameshift product (Supplementary Figure E7), whereas six of the pathogenic frameshift mutations introduce additional Cys (1–3 residues) in the frameshift products (Supplementary Figure E7). In contrast, out of the 23 pathogenic frameshift mutations, 17 (74%) reduce the number of Ser residues in the frameshift products compared to the wild-type of the same length span; the range of reduction found is by 1–30 residues (Supplementary Figure E9). We have summarized these findings in Figure 5A, showing that 17 out of 23 mutations increase the content of the potential ubiquitination sites (compared to the wild-type of the same length span) and highlights substantial differences between different mutations.

Because none of the recurrent frameshift mutations’ products reduced the number of Lys residues, and the vast majority led to the introduction of Thr and/or Cys and a reduction of Ser residues, we wanted to check if this is also the case for the non-recurrent rare family-specific frameshifts. To this end, we selected all the frameshifts located within the coding DNA reference sequence NM_002016.2: c. 1 to c.5700. Out of 101 rare frameshift mutations analysed, 70 (70%) introduced additional Lys residues in the frameshift products, introducing 1 to 8 additional Lys residues compared to the same length span in the wild-type sequence; the number of Lys remained the same in 28 frameshifts. In contrast, we only encountered a reduction in the number of Lys residues on three occasions (3%), namely, in the frameshift mutation p.Ser798ArgfsTer19 or c.2394delC, p.Lys801SerfsTer15 or c.2402_2405delAACA and p.Lys255IlefsTer2 or c.762_766delCAAAA; in all those cases, one Lys residue was lost compared to the same length wild-type sequence span (Supplementary Table E3). Out of the 101 rare frameshift mutations analysed, 66 introduced additional Thr residues (ranging from 1 to 25) in the frameshift product, whereas seven reduced the number of Thr residues in the frameshift product (reduction range 1–3 residues). Similarly, 45 of those introduced Cys (one to three residues) in the frameshift product. In contrast, 82 generated a frameshift product with a reduction of Ser (ranging from 1 to 57 residues). Only two frameshifts introduced additional Ser in the frameshift product (one to two additional residues) (Supplementary Table E3). Regardless of their position, all frameshifts translate into products shorter than the wild-type, with their product length varying between 1 and 260 aa.

A higher number of ubiquitination-prone residues in a protein sequence may promote its ubiquitination (Mattiroli and Sixma, 2014). Thus, we also determined the ratio of those to the full product length, nonsense, and frameshift *FLG* mutations. We found that irrespective of the kind of mutation, all have an increased ratio of Lys residues vs. the product length compared to the respective ratio calculated for the wild-type profilaggrin (Figure 5B), probably due to a high density of Lys residues in the N-terminal domain. As for the residues that may undergo non-canonical ubiquitination, a decreased ratio of Ser residues (Figure 5C) and an increased ratio of Thr residues (Figure 5D) was found as a general rule, while the relative content of the Cys residues was different for the two types of *FLG* mutations; with a reduction of the ratio for the nonsense mutations and a spectrum of the ratio values for the frameshift mutations, spanning the value obtained for the wild-type (Figure 5E).

### Filaggrin mutations change C-terminal stability and introduce degron motifs

**FIGURE 6:**
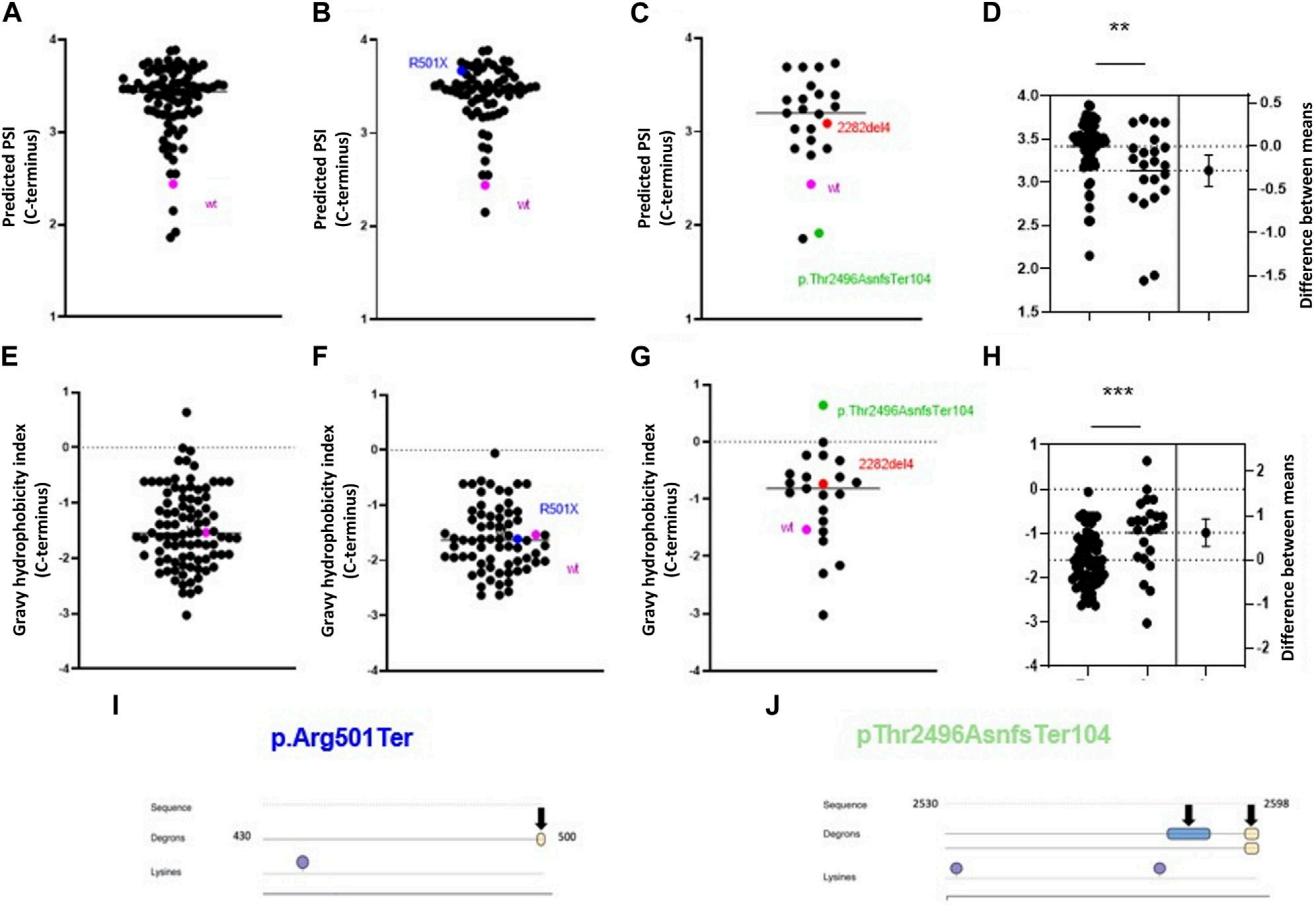
Alterations in profilaggrin stability in FLG null mutation carriers. **(A–D)** C-terminal PSI of recurrent pathogenic frameshift **(C)**, nonsense mutation **(B)** and combined **(A)** here wild type is shown as pink and two most common European mutations 2282del4 and R500X are marked in red and blue; **(D)** estimation plot and comparison between PSI scores; Student’s t-test; *p* < 0.01 (**); **(E–H)** C terminal Gravy hydrophobicity index of recurrent pathogenic frameshift **(G)**, nonsense mutation **(F)** and combined **(E)** here wild type is shown as pink and two most renowned mutation 2282del4 and R500X are marked in red and blue; **(H)** estimation plot and comparison between PSI scores; Student’s t-test; *p* < 0.001 (***); **(I)** Location of C-terminal degron in p.Arg501Ter and Lysine residue. **(J)** Location of C terminal degrons and lysine residues in the p.Thr2496AsnfsTer104.

Some pathogenic mutations in *FLG* lead to changes in the aa sequence and the appearance of an altered C-terminus. Interestingly, we found that almost all these mutations are predicted to lead to increased C-terminus stability compared to the wild-type (Figure 6A), and this was similar for nonsense and frameshift mutations (Figures 6B, C); albeit the PSI scores were significantly lower for the latter (Figure 6D). Here, we noted that the common nonsense mutation R501X (p.Arg501Ter or c.1501C>T) had higher PSI in comparison to the common frameshift mutation 2282del4 (p.Ser761CysfsTer36), despite generating mutant proteins of relatively similar lengths. Interestingly, we found the median GHI for all the mutations analysed to be in a similar range to the wild-type protein (−1.547 vs. −1.533, respectively) (Figure 6E), and their negative value points out that their nature is hydrophilic and potentially less prone to regulation by the C-degron pathway. However, we observed a difference in the median GHI between the nonsense and frameshift mutations; again, we observed a discrepancy between the most common European variants (Figures 6F–H). Several pathogenic frameshift mutations additionally introduce degron motifs absent in the profilaggrin (Supplementary Table E4), which may have a reducing impact on the stability of the mutant product, including R501X (Figure 6I). E.g., p.Thr2496AsnfsTer104, which is the only variant with a hydrophobic C-terminus and predicted as destabilizing (Figure 6J), yields two novel primary degron sites. The APC/C (DBOX) motif occurs internally at 2,580–2,588, while the C-terminus carries a sequence corresponding to two known C-degron motifs, both carrying Arg at the antepenultimate position. Importantly, over 25% of the proteins carrying these C-degron motifs are substrates of the cullin-RING E3 ligases (Koren et al., 2018). These results point to the potentially destabilizing character of this frameshift variant, whereas other frameshift mutations moderately improve the stability of their C-termini. Data summarizing predicted C-terminal PSI, GHI, and found degron motifs in the analysed pathogenic frameshift and nonsense variants are available in Supplementary Table E4, and the predicted degradation pathways are shown in Tables 2, 3.

**TABLE 2:** List of recurrent pathogenic nonsense mutation and their degrons in comparison to the wild-type profilaggrin.

E3 ligase-binding motifs			Mutation
N-terminus	Internal	C-terminus	
Ac/N degron; motif recognized by Teb4 and Not4 E3 ligases	SPOP, APC/C (ABBA, KEN, DBOX), SCF-TRCP1 recognition motifs	—	Full-length WT
		Motif -G end, likely CRL substrate	p.Lys4022Ter
		—	p.Arg3879Ter
		Motif -R end, likely CRL substrate	p.Gln3859Ter
		Motif -EE end, likely CRL substrate	p.Gln3818Ter
		—	p.Ser3749Ter
		Motif -A end, likely CRL substrate	p.Arg3743Ter
		Motif -G end, likely CRL substrate	p.Gln3684Ter
		—	p.Arg3657Ter
		Motif R at -3, likely CRL substrate	p.Gln3520Ter
		—	p.Arg3442Ter
		Motif -A end, likely CRL substrate	p.Arg3419Ter
		Motif -G end, likely CRL substrate	p.Arg3409Ter
			p.Ser3316Ter
		Motif A at -2, likely CRL substrate	p.Ser3296Ter
		—	p.Ser3247Ter
		—	p.Gln3029Ter
		—	p.Arg3009Ter
		Motif -A end, likely CRL substrate	p.Arg2971Ter
		—	p.Trp2907Ter
		—	p.Ser2706Ter
		—	p.Arg2685Ter
		—	p.Arg2613Ter
		—	p.Ser2554Ter
		—	p.Ser2544Ter
		—	p.Ser2453Ter
		Motif -A end, likely CRL substrate	p.Arg2447Ter
		—	p.Glu2422Ter
		—	p.Gln2417Ter
		—	p.Gln2397Ter
		—	p.Arg2361Ter
		—	p.Ser2344Ter
		Motif -A end, likely CRL substrate	p.Ser2317Ter
		—	p.Gly2228Ter
		—	p.Tyr2092Ter
		Motif R at -3, likely CRL substrate	p.Ser2080Ter
		—	p.Gln2070Ter
		—	p.Arg2037Ter
		—	p.Ser1977Ter
		—	p.Trp1947Ter
		—	p.Ser1906Ter
		—	p.Gly1826Ter
		—	p.Arg1798Ter
		—	p.Glu1795Ter
		—	p.Gln1790Ter
		—	p.Ser1733Ter
		Motif -EE end, likely CRL substrate	p.Ser1729Ter
		—	p.Gly1724Ter
		—	p.Arg1712Ter
		—	p.Gln1701Ter
		—	p.Ser1695Ter
		—	p.Ser1515Ter
		Motif -A end, likely CRL substrate	p.Arg1474Ter
	SPOP, APC/C (ABBA, KEN), SCF-TRCP1 recognition motifs	Motif R at -3, likely CRL substrate	p.Ser1302Ter
		—	p.Gln1256Ter
		—	p.Gly1253Ter
		Motif -G end, likely CRL substrate	p.Arg1140Ter
		—	p.Gly1139Ter
		—	p.Ser1040Ter
		Motif -A end, likely CRL substrate	p.Ser1020Ter
		—	p.Gln977Ter
		Motif -A end, likely CRL substrate	p.Arg826Ter
		—	p.Arg788Ter
	SPOP, APC/C (ABBA, KEN) recognition motifs	—	p.Arg740Ter
		—	p.Ser609Ter
		Motif -A end, likely CRL substrate	p.Arg501Ter
	APC/C (ABBA, KEN) recognition motifs	—	p.Gln355Ter
		—	p.Trp326Ter
		—	p.Ser260Ter
		—	p.Lys182Ter
Sequence too short for analysis			p.Glu32Ter

**TABLE 3:** List of recurrent pathogenic frameshift mutation and their degron motif.

E3 ligase-binding motifs			Mutation
N-terminus	Internal	C-terminus	
Ac/N degron; motif recognized by Teb4 and Not4 E3 ligases	SPOP, APC/C (ABBA, KEN, DBOX), SCF-TRCP1 recognition motifs	—	p.His3951ProfsTer4
		—	p.Arg3272AsnfsTer118
		APC/C motif	p.Ala3094HisfsTer37
		—	p.Ser2649ValfsTer94
		Motif R at -3, likely CRL substrate	p.Thr2496AsnfsTer104
		—	p.Gln2423ValfsTer2
		—	p.Ser2366ArgfsTer52
		—	p.His1897ProfsTer198
		—	p.Glu1605ThrfsTer103
		—	p.Ser1595ArgfsTer110
		—	p.Ser1235HisfsTer211
	SPOP, APC/C (ABBA, KEN), SCF-TRCP1 recognition motifs	—	p.Ser1171GlnfsTer15
		—	p.Gly1109GlufsTer13
		Destabilizing motif VxT	p.Gln1084ValfsTer21
		—	p.Ser761CysfsTer36
	SPOP, APC/C (ABBA, KEN) recognition motifs	—	p.Asp433HisfsTer43
		Motif R at −3, likely CRL substrate	p.Ser417ValfsTer2
	APC/C (ABBA, KEN) recognition motifs	—	p.Ser249LysfsTer10
		Motif -EE end, likely CRL substrate	p.Gly221GlufsTer3
		—	p.Asn186LysfsTer4
		—	p.Glu160ArgfsTer10
		—	p.Arg130GlufsTer6

## Discussion

Expression of profilaggrin and its processing into filaggrin monomer units, as well as their further breakdown into the components of the NMF, is essential for the functionality of the epidermal barrier; disturbances at any point of this intricate process are detrimental and lead to pathology. Given their role in the collapse of the keratin-based cytoskeleton and the striking effect on nuclear integrity, it is apparent why the accumulation of free filaggrin monomers into the cytosol of a keratinocyte is toxic to the cell and initiates its programmed death (Dale et al., 1997; Kuechle et al., 2000; Presland et al., 2001). Hence, both the released filaggrin monomers and any free profilaggrin molecules that could be processed must be under rigorous control in the cytosol to prevent premature cell death and allow for keratinocyte differentiation and stratification, critical for the formation of the functional skin barrier. Such control may be executed by different means, including protein sequestration, removal of the excess from the cytosol, and re-direction for turnover by the UPS.

We have previously described two separate mechanisms that control intracellular filaggrin levels during keratinocyte differentiation; the actin-based Akt-1/HspB1-dependent mechanism governing profilaggrin sequestration in KHGs (Gutowska-Owsiak et al., 2018) as well as the small extracellular vesicle (sEV)-mediated removal of excess free-floating profilaggrin/filaggrin from the cytosol (Gutowska-Owsiak, 2022). As for the latter, we also determined that *Staphylococcus aureus*, a skin pathogen with high prevalence and significant contribution to the pathology in AD patients, enhances filaggrin loading into the sEV cargo, facilitating its removal from the skin. Modeling protein networks indicated that the link between TLR2 signaling, profilaggrin processing, and cargo loading into the sEVS might include proteins involved in ubiquitination, pointing to protein degradation and intracellular trafficking. Indeed, the importance of protein turnover has been shown previously to be critical for controlling cellular protein abundance (Yen et al., 2008; Cambridge et al., 2011), and we envisage that both subcellular localization and propensity for degradation are key for homeostasis within the profilaggrin/filaggrin system. Ubiquitination is important for protein trafficking to diverse cellular localizations, including the compartments of the endocytic system, as well as nuclear localization aiding gene regulation. These pathways are also exploited by pathogenic bacteria and viruses for their successful transmission and immune evasion (Millar et al., 2019; Tokheim et al., 2021; Kampmeyer et al., 2022; Moosbrugger-Martinz et al., 2022).

The importance of the proteasome in profilaggrin turnover and control of its intracellular fate can be speculated already based on the appearance of greatly enlarged KHGs (hypergranulosis) in the skin of patients with an autosomal recessive epidermal abnormality known as keratosis linearis with ichthyosis congenita and sclerosing keratoderma (KLICK) syndrome (Vahlquist et al., 1997)^,^ (Dahlqvist et al., 2010)^,^ (Takeichi and Akiyama, 2020). Those patients have a deletion of a single nucleotide at the 5’ untranslated region of the proteasomal maturation protein (POMP), a chaperon that mediates stabilization of the proteasome complex; thus, loss of function leads to insufficiency of the proteasome in differentiating keratinocytes (Dahlqvist et al., 2010; Morice-Picard et al., 2017; Onnis et al., 2018). KLICK patients also demonstrate thickened SC and aberrant filaggrin staining with an antibody directed against filaggrin monomer (Dahlqvist et al., 2010).

In this study we were able to confirm the involvement of the UPS in profilaggrin degradation using a proteasome inhibitor MG132 and deubiquitinases inhibitor PR-619. Treatment with these compounds allowed us to observe the accumulation of profilaggrin and high molecular weight processed products in the lysates, suggesting reduced profilaggrin turnover likely underlying hypergranulosis in the KLICK syndrome. At the same time, the reduction in keratinocyte viability after the overnight treatment could result from the accumulation of the free unsequestered/uncontrolled monomer filaggrin units (Presland et al., 2001). Unfortunately, dead cells where this could be potentially detectable were not evaluated in this study.

Our findings are in a sharp contrast to just published short communication by Briot et al. who failed to observe a difference upon proteasome inhibition and speculated that filaggrin monomer is not degraded via proteasome (Briot et al., 2023). We believe that this discrepancy results from the use of different models. Specifically, we used cells growing in monolayers, where many cells present with low, but detectable profilaggrin expression in relatively undifferentiated cells (Gutowska-Owsiak, 2022). This is where the greatest control over free profilaggrin must be exerted and where it could undergo the UPS-mediated turnover. With increased profilaggrin expression in the 3D epidermal equivalent used by Briot et al., the vast majority of the protein is already sequestered within KGHs, therefore the risk of keratin aggregation by free cytosolic filaggrin is reduced. Importantly, such containment of the protein within KHGs prevents its access to the proteasome, which is not able to sample from those insoluble organelles. The importance of UPS-mediated control over profilaggrin is also supported by the biological pattern of the proteasome expression, which functions primarily in the undifferentiated keratinocytes, corresponding to basal and suprabasal epidermal layers (Zieba et al., 2017); proteasome gets disassembled in the cells at the late stages of differentiation, which results from regulation of POMP expression.

To get a global picture of features important from the perspective of regulation by the UPS, it is important to consider multiple factors, i.e., internal degron motifs, different properties of the N-/C-termini, such as their stability and hydrophobicity, structural features of the protein with regards to the solvent accessibility and IDRs, and residues that may undergo ubiquitination. Here, we determined sequence characteristics by identifying the degron sequences with the DEGRONOPEDIA web server, complemented with the detailed analysis of PTMs, i.e., ubiquitination and phosphorylation with the potential to govern profilaggrin/filaggrin degradation and cellular trafficking, allowing us to propose likely degradation pathways. Protein degradation via the UPS often requires the formation of the Lys-linked polyubiquitin chains, while the nuclear re-directing depends on the Lys monoubiquitination (Trotman et al., 2007). Apart from the Lys residues, the profilaggrin sequence contains multiple Cys, Ser, and Thr residues; such residues have been shown to undergo ubiquitination at these marks. Furthermore, it has been shown that such non-canonical ubiquitination is less thermodynamically stable (McDowell and Philpott, 2013; McClellan et al., 2019; Squair and Virdee, 2022) than the canonical Lys ubiquitination and predominantly drives proteasomal degradation over protein sorting (McClellan et al., 2019). The high enrichment of the canonical ubiquitination sites in the N-terminal domains of profilaggrin and the accumulation of the non-canonical ubiquitination sites within the monomer repeats are interesting observations and suggest potential differences in the degradation pathways between the domains. The N-terminal domain is involved in nuclear signaling events that initiate a positive feedback loop for profilaggrin expression and keratinocyte differentiation. Proteasome activity is considered high in the nucleus, thus, increased predicted stability values could potentially indicate relative resistance of the profilaggrin N-terminal domain.

Furthermore, we found that the entire profilaggrin sequence contains 18 putative primary degron sites. Our screening suggests that the unprocessed profilaggrin protein is likely degraded upon recognition of the Ac/N-degron motif as the second N-terminal residue is Ser and Nα-terminal acetylation (Nt-acetylation) of nascent proteins, whose N-terminus contains Met or a small uncharged residue alanine, Cys, Val, Ser or Thr as these become N-terminal after co-translational removal of Met by Met-aminopeptidase), is one of the most significant and common protein modifications occurring on ∼80% of all human proteins (van Damme et al., 2012; Aksnes et al., 2016). Nt-acetylation can serve as a degron motif for the two E3 ligases: March6/TEB4, a Really Interesting New Gene (RING)-type E3 ligase located in the endoplasmic reticulum membrane, and NOT4, a component of the CCR4-NOT multi-subunit complex, which induces substrate protein degradation via the Ac/N-degron pathway (Varshavsky, 2011; Shemorry et al., 2013; Park et al., 2015; Lee et al., 2016).

The presence of 17 internal degrons in wild-type profilaggrin indicates the possibility of its N-/C-terminus-independent degradation. Among these degrons, the destruction box (DBOX), KEN box, and ABBA motifs are found and substrates containing one or more of these sequences typically undergo polyubiquitylation by the anaphase-promoting complex (APC/C) (Brown et al., 2014). APC/C is a large, multi-subunit E3 ligase that regulates cell cycle progression in eukaryotes. Importantly, it is also involved in proliferation and differentiation regulation in human primary keratinocytes (Quek et al., 2018). Hence, it is probable that APC/C could influence the keratinocyte life cycle and contribute to the disruption in epidermal homeostasis, such as that seen in AD, at least partly through its effect on profilaggrin degradation.

SCFβ-TRCP E3 ligase complex regulates proteasome-dependent degradation of various substrates, including early mitotic inhibitor 1 (EMI1) (Margottin-Goguet et al., 2003), cell cycle homolog 25 (CDC25A) (Busino et al., 2003; Jin et al., 2006) and vascular endothelial growth factor receptor 2 (VEGFR2) (Shaik et al., 2012). Profilaggrin also contains the consensus degron sequence (D(S)Gx{2,3}([ST]) of SCF^β−TRCP^ where Ser/Thr residues should be phosphorylated for proper motif recognition (x—any aa) (Winston et al., 1999; Guharoy et al., 2016). Interestingly, this motif was shown to be phosphorylated in rat profilaggrin (Resing et al., 1995) but neither the iPTMNet (Huang et al., 2018) nor PhoshoSitePlus (Hornbeck et al., 2015) databases, incorporated into the DEGRONOPEDIA, record any phosphorylation within them.

Profilaggrin has also been proposed as a binding partner of receptors of the cullin E3 ligases: FBXW7 (Xu et al., 2021), DTL (Huttlin et al., 2021), and VHL (Ewing et al., 2007). Present in proliferating cells, FBXW7 (F-Box and WD Repeat Domain Containing 7) is a member of the F-box family of proteins and functions as a substrate recognition element of the SCF E3 ligase. FBXW7 isoforms recognize their substrates through the CDC4 phosphodegron (CPD) motif so that they can be ubiquitinated and targeted for degradation by the proteasome along with the substrate. Substrates of FBXW7 containing CPD variants include MYC, Cyclin E/cyclin-dependent kinase CDK2, JUN, Myeloid cell leukemia-1 (MCL-1), mammalian target of rapamycin (mTOR), and NOTCH1 (Koepp et al., 2001; Wei et al., 2005; Mo et al., 2007; Mao et al., 2008; Inuzuka et al., 2011; King et al., 2013; Yeh et al., 2018). Analysis of the profilaggrin sequence did not reveal the occurrence of CPD motifs, which may suggest a profilaggrin-specific degron/phosphodegron that requires further identification. DTL is another receptor likely to bind to profilaggrin; it associates with CRL4A (cullin 4A-RING ubiquitin ligase) and regulates DNA replication and the cell cycle by regulating proteins such as CDT1, PR-Set7/Set8/KMT5A and p21 (Jin et al., 2006; Abbas and Dutta, 2009; Centore et al., 2010; Shibata et al., 2011; Cui et al., 2019). Since DTL prevents DNA damage after UV irradiation (Ishii et al., 2010), we hypothesize its involvement in skin function, possibly through modulation of profilaggrin or filaggrin monomer levels. In addition, profilaggrin was detected as an interactor of cullin-2 (CUL2)-based E3 ligases (Bennett et al., 2010) and VHL (von Hippel-Lindau). CUL2 is a platform for the Elongin B and Elongin C adaptor protein complex, which interact with various substrate receptors, such as the VHL tumor suppressor protein (Gossage et al., 2014; Cai and Yang, 2016). Since VHL plays a role in skin inflammation, CUL2 and VHL may regulate profilaggrin in psoriasis (Martínez-Torres et al., 2022).

Our analysis indicated that the monomers generated by SASPase cleavage are relatively stable, given their minor decrease in the N-terminal PSI, but a significant increase in the C-terminal PSI and a drop in GHI value; this likely permits their long-lasting functionality required for cross-linking of the proteins within the cornified envelope, and aggregation and collapse of the IF-based cytoskeleton. Intriguingly, the UPS system has been previously implicated in the degradation and turnover of keratins (Yamazaki et al., 2012); increased keratin turnover is observed in a subtype of epidermolysis bullosa simplex (EBS) patients, resulting in shortened lifespan, replicative senescence, and decreased cellular resistance in keratinocytes (Logli et al., 2022).

It is important to note that the UPS-mediated degradation is a pathway separate from the pathway of profilaggrin processing to monomers and subsequently into the compounds of the NMF, which occurs predominantly in the SC containing no living cells. UPS components primarily function in the intracellular environment; however, both the ubiquitinating enzymes and proteasome are also present and active in the extracellular spaces and body fluids (Takada et al., 1997; Sixt and Dahlmann, 2008; Majetschak, 2011; Ben-Nissan et al., 2022), potentially also within the SC. However, the difference in pH could affect the efficiency of the degradation process, given the impact of the acidic environment. All three hydrolytic activities (chymotrypsin-like, trypsin-like, and caspase-like) of the 26S proteasome decreased when the pH was lowered from 7.5 to 7.0 (Ugai et al., 1993; Klinkradt et al., 1997). At the same time, the 20S proteasome from human platelets displayed chymotrypsin-like activity with an optimum pH of 5.0–5.5 (Ostrowska et al., 2009). It can be speculated that similar 20S activity may be maintained within the SC, enabling partial degradation of targeted proteins. Changes in pH may also affect the ubiquitination process, e.g., pH lower than 7.5 destabilized the APC/C complex (Passmore et al., 2005), and we identified potential APC/C degrons in profilaggrin. These observations may suggest that profilaggrin and its derivatives do not undergo intense turnover in the SC.

For this study, we assumed that profilaggrin processing may coincide with the UPS-mediated degradation, at least to some extent. On the other hand, while the majority of profilaggrin processing to monomers can be localized to the SC, i.e., after keratinocyte death, it is unclear how much processing may take place in the live epidermal layers. However, small but detectable amounts of the filaggrin monomer have been observed by Western blot in keratinocytes grown in 2D cultures which were not stratified (Dang et al., 2016), so we could envisage that the process likely begins much earlier, albeit probably at a reduced rate, since the highest expression of the processing enzymes was demonstrated for the top epidermal layers (Miyachi et al., 1986; Pearton et al., 2001; Bernard et al., 2005).

Here we also determined that the common *FLG* mutations predisposing to AD result in the generation of new products with a different propensity for degradation, supported by changes in stability and ubiquitination-prone residues as well as the emergence of novel degrons. This may suggest that both the filaggrin turnover and organellar distribution in the skin of the patients are affected, and the generated products may undergo altered cellular fates. Indeed, in the case of several mutations, we noted the accumulation of all those pro-degradation motifs. At the same time, while introducing several new ubiquitination-prone residues compared to the wild-type of the same length, one of the most common pathological *FLG* mutations, 2282del4, does not introduce any novel known degrons. However, it is important to note that it is not clear to what extent profilaggrin could undergo modification at all these potentially ubiquitinated residues; since the protein is known to undergo many different modifications at Lys residues.

*FLG* mutations resulting in the loss of the C-terminal domain led to practically nonfunctional monomers generated from this allele (Presland et al., 2000; Sandilands et al., 2007); hence, it has been long assumed that the profilaggrin C-terminal domain is critical for processing of the protein. However, it is not clear whether this is of crucial importance, as no experimental data on the C-terminal domain’s involvement in the processing has yet been published. Here, we determined that the C-terminal sequence of the wild-type filaggrin has a stability score in the range that is typical for most human proteins, therefore not allowing us to consider this to be a highly stabilizing part for the entire profilaggrin molecule.

Interestingly, we found the C-terminal PSI scores to be increased for nearly all the mutated products, complicating the picture further. It could be speculated that the increased stability of the mutated product would result in the accumulation of such protein intracellularly, albeit likely not in the form of granules (Sybert et al., 1985). However, there is no evidence in the samples from the patients, and certainly, no evidence indicating increased processing of the protein as this would result in high amounts of NMF being generated and barrier function maintenance. It is possible that in this scenario, the remaining routes (proteasomal degradation, vesicular export) would be enhanced to control the profilaggrin/filaggrin levels in the cytosol. Indeed, we have previously made a very unexpected observation of the increased amount of the profilaggrin/filaggrin protein cargo in the sEVs fractions isolated from the blood of AD patients compared to the healthy controls (Gutowska-Owsiak, 2022), and this would perfectly align with such a scenario. Thus, it is conceivable that keratinocytes could expel the excess profilaggrin/filaggrin-related products to prevent the toxic consequences in the event of a diminished capacity to generate KHGs (Sybert et al., 1985). It would require additional experimental work beyond the scope of this study to determine if the mutated products can indeed be detected in the sEVs found in the blood of the patients.

In summary, we determined that proteasome-mediated profilaggrin degradation is one of the means of controlling intracellular levels of the protein in keratinocytes, and we identified critical components regulating UPS-mediated processing (ubiquitination-prone residues and degrons) within its sequence. Furthermore, we also described how *FLG* mutations might affect the stability of mutant proteins and potential degradation routes in the cell.

## Figures and Tables

**Table:** 

**AA**	amino acid
**AD**	atopic dermatitis
**APC/C**	anaphase-promoting complex
**CE**	cornified envelope
**CPD**	CDC4 phosphodegron
**CUL**	cullin
**CHIP**	C-terminus of HSC70-interacting protein
**DBOX**	destruction box motif
**E1**	ubiquitin-activating enzyme
**E2**	ubiquitin-conjugating enzyme
**E3**	ubiquitin-ligase enzyme
**EBS**	epidermolysis bullosa simplex
**EDC**	epidermal differentiation complex
**EMI1**	early mitotic inhibitor
**GHI**	gravy hydrophobicity index
**IDR**	intrinsically disordered region
**IF**	intermediate filament
**IV**	ichthyosis vulgaris
**KHG**	keratohyalin granule
**KMT**	Lysine methyltransferase
**LOF**	loss of function
**MCL1**	myeloid cell leukemia 1
**mTOR**	mammalian target of rapamycin
**NMF**	natural moisturizing factor
**PC**	precursor converting enzyme
**FLG**	profilaggrin gene
**PSI**	protein stability index
**PCA**	pyrrolidone carboxylic acid
**PTM**	post-translational modification
**RING**	really Interesting New Gene
**SASPase**	skin specific retroviral aspartic protease
**SFTP**	S100 fused-type protein
**SC**	stratum corneum
**SCFβ-TRCP**	Skp1-cullin 1-F-box with β-transducin repeat-containing protein acting as its substrate receptor
**sEV**	small extracellular vesicle
**SPOP**	speckle-type POZ protein
**TLR**	toll-like receptor
**Ub**	ubiquitin
**UCA**	urocanic acid
**UPS**	ubiquitin-proteasome system
**VEGFR2**	vascular endothelial growth factor receptor 2

## Data Availability

The original contributions presented in the study are included in the article/Supplementary Material, further inquiries can be directed to the corresponding authors.
