# Supplementary material for: In silico analysis of the profilaggrin sequence indicates alterations in the stability, degradation route, and intracellular protein fate in filaggrin null mutation carriers
Source: Front Mol Biosci. 2023 May 2;10:1105678. doi: 10.3389/fmolb.2023.1105678 (PMC10185843; doi:10.3389/fmolb.2023.1105678)
Supplement: Supplementary file 1 [file Table1.DOCX]

**Supplementary materials**

**Table E1. Degron motifs and stability of C-termini upon PC cleavage of FLG.**

| **Indices** | **Degrons at new N-/C-terminus** | **Gravy index of new N-/C-terminus** | **Machine Learning: predicted stability of new N-/C-terminus** |
| --- | --- | --- | --- |
| 1-288 | None | C-terminus: -1.76 | C-terminus: 3.48 |
| 289-4061 | M{0,1}[AST]x (Ac/N degron); recognized by S. cerevisiae Doa10 and its mammalian counterpart Teb4, and also Not4, the E3 subunit of Ccr4-Not | N-terminus: -1.533 | N-terminus: 4.15 |

**Table E2. Degron motifs and stability of C-termini upon SASPase cleavage of FLG.**

| **Indices** | **Degrons at new N-/C-terminus** | **Gravy index of new N-/C-terminus** | **Machine Learning: predicted stability of new N-/C-terminus** |
| --- | --- | --- | --- |
| 289-470 | M{0,1}[AST]x (Ac/N degron); recognized by S. cerevisiae Doa10 and its mammalian counterpart Teb4, and also Not4, the E3 subunit of Ccr4-Not | N-terminus: -2.413  C-terminus: -1.34 | N-terminus: 4.15  C-terminus: 3.41 |
| 471-795 | M{0,1}([NQ])x (Arg/N tertiary degron); recognized by S. cerevisiae Ubr1 E3, the mammalian Ubr1, Ubr2, Ubr4, and Ubr5 E3s, the Prt1 and Prt6 E3s of plants, and the mammalian non-E3 autophagy regulator p62/Sqstm1 | N-terminus: -1.593  C-terminus: -1.58 | N-terminus: 3.41  C-terminus: 3.33 |
| 796-1119 | M{0,1}([NQ])x (Arg/N tertiary degron); recognized by S. cerevisiae Ubr1 E3, the mammalian Ubr1, Ubr2, Ubr4, and Ubr5 E3s, the Prt1 and Prt6 E3s of plants, and the mammalian non-E3 autophagy regulator p62/Sqstm1 | N-terminus: -1.5  C-terminus: -0.813 | N-terminus: 3.40  C-terminus: 2.75 |
| 1120-1443 | M{0,1}([NQ])x (Arg/N tertiary degron); recognized by S. cerevisiae Ubr1 E3, the mammalian Ubr1, Ubr2, Ubr4, and Ubr5 E3s, the Prt1 and Prt6 E3s of plants, and the mammalian non-E3 autophagy regulator p62/Sqstm1 | N-terminus: -1.54  C-terminus: -1.06 | N-terminus: 3.48  C-terminus: 3.40 |
| 1444-1767 | M{0,1}([NQ])x (Arg/N tertiary degron); recognized by S. cerevisiae Ubr1 E3, the mammalian Ubr1, Ubr2, Ubr4, and Ubr5 E3s, the Prt1 and Prt6 E3s of plants, and the mammalian non-E3 autophagy regulator p62/Sqstm1 | N-terminus: -1.647  C-terminus: -1.033 | N-terminus: 3.45  C-terminus: 3.26 |
| 1768-2092 | M{0,1}([NQ])x (Arg/N tertiary degron); recognized by S. cerevisiae Ubr1 E3, the mammalian Ubr1, Ubr2, Ubr4, and Ubr5 E3s, the Prt1 and Prt6 E3s of plants, and the mammalian non-E3 autophagy regulator p62/Sqstm1 | N-terminus: -1.54  C-terminus: -0.787 | N-terminus: 3.44  C-terminus: 3.39 |
| 2093-2416 | M{0,1}([NQ])x (Arg/N tertiary degron); recognized by S. cerevisiae Ubr1 E3, the mammalian Ubr1, Ubr2, Ubr4, and Ubr5 E3s, the Prt1 and Prt6 E3s of plants, and the mammalian non-E3 autophagy regulator p62/Sqstm1 | N-terminus: -1.647  C-terminus: -0.747 | N-terminus: 3.47  C-terminus: 3.24 |
| 2417-2740 | M{0,1}([NQ])x (Arg/N tertiary degron); recognized by S. cerevisiae Ubr1 E3, the mammalian Ubr1, Ubr2, Ubr4, and Ubr5 E3s, the Prt1 and Prt6 E3s of plants, and the mammalian non-E3 autophagy regulator p62/Sqstm1 | N-terminus: -1.54  C-terminus: -0.76 | N-terminus: 3.46  C-terminus: 3.33 |
| 2741-3064 | M{0,1}([NQ])x (Arg/N tertiary degron); recognized by S. cerevisiae Ubr1 E3, the mammalian Ubr1, Ubr2, Ubr4, and Ubr5 E3s, the Prt1 and Prt6 E3s of plants, and the mammalian non-E3 autophagy regulator p62/Sqstm1 | N-terminus: -1.473  C-terminus: -0.76 | N-terminus: 3.35  C-terminus: 3.33 |
| 3065-3388 | M{0,1}([NQ])x (Arg/N tertiary degron); recognized by S. cerevisiae Ubr1 E3, the mammalian Ubr1, Ubr2, Ubr4, and Ubr5 E3s, the Prt1 and Prt6 E3s of plants, and the mammalian non-E3 autophagy regulator p62/Sqstm1 | N-terminus: -1.473  C-terminus: -1.1 | N-terminus: 3.35  C-terminus: 3.29 |
| 3389-3712 | M{0,1}([NQ])x (Arg/N tertiary degron); recognized by S. cerevisiae Ubr1 E3, the mammalian Ubr1, Ubr2, Ubr4, and Ubr5 E3s, the Prt1 and Prt6 E3s of plants, and the mammalian non-E3 autophagy regulator p62/Sqstm1 | N-terminus: -1.54  C-terminus: -0.747 | N-terminus: 3.48  C-terminus: 3.24 |
| 3713-4061 | M{0,1}([NQ])x (Arg/N tertiary degron); recognized by S. cerevisiae Ubr1 E3, the mammalian Ubr1, Ubr2, Ubr4, and Ubr5 E3s, the Prt1 and Prt6 E3s of plants, and the mammalian non-E3 autophagy regulator p62/Sqstm1 | N-terminus: -1.373  C-terminus: -1.533 | N-terminus: 3.45  C-terminus: 2.44 |

**Table E3: List of frameshift mutations examined for this study.**

| **Occurrence**  **& Pathogenicity** | **ID** | **Protein consequence of FS** | **Transcript consequence of FS** | **Number of Ub conjugating residues in FS product** | | | | **Number of Ub conjugating residues in the corresponding WT seq** | | | | **Ethnic specificity** |
| --- | --- | --- | --- | --- | --- | --- | --- | --- | --- | --- | --- | --- |
|  |  |  |  | **K** | **S** | **T** | **C** | **K** | **S** | **T** | **C** |  |
| Recurrent: Pathogenic frameshifts | rs781161516 | p.His3951ProfsTer4 | c.11851dupC | 0 | 0 | 0 | 0 | 0 | 2 | 0 | 0 | African American |
|  | rs777960054 | p.Arg3272AsnfsTer118 | c.9815_9818delGACA | 3 | 9 | 11 | 1 | 0 | 30 | 3 | 0 | Asian |
|  | rs777848510 | p.Ala3094HisfsTer37 | c.9280delG | 1 | 1 | 8 | 0 | 0 | 7 | 1 | 0 | European |
|  | rs757903351 | p.Ser2649ValfsTer94 | c.7945delA | 5 | 8 | 14 | 1 | 0 | 23 | 2 | 0 | Asian |
|  | rs774362740 | p.Thr2496AsnfsTer104 | c.7487delC | 3 | 2 | 13 | 0 | 0 | 29 | 3 | 0 | Asian |
|  | rs760831749 | p.Gln2423ValfsTer2 | c.7267_7268delCA | 0 | 0 | 0 | 0 | 0 | 0 | 0 | 0 | European |
|  | rs377017648 | p.Ser2366ArgfsTer52 | c.7098_7101delTCAG | 2 | 5 | 6 | 1 | 0 | 13 | 2 | 0 | European |
|  | rs1404928962 | p.Ser2317Ter | c.6950_6957delCATCCCAT | 0 | 0 | 0 | 0 | 0 | 0 | 0 | 0 | Asian |
|  | rs1246063885 | p.His1897ProfsTer198 | c.5690delA | 8 | 13 | 25 | 1 | 2 | 52 | 4 | 0 | European |
|  | rs773221702 | p.Glu1605ThrfsTer103 | c.4808_4812dupACTCA | 5 | 5 | 8 | 0 | 0 | 26 | 4 | 0 | Asian |
|  | rs533784172 | p.Ser1595ArgfsTer110 | c.4785_4788delTCAG | 5 | 5 | 10 | 0 | 0 | 28 | 4 | 0 | African American, Latino, European, Asian |
|  | rs397507563 | p.Ser1235HisfsTer211 | c.3702delG | 9 | 14 | 28 | 3 | 1 | 58 | 5 | 0 | European, Asian, Ashkenazi Jewish, African American, |
|  | rs1370544496 | p.Ser1171GlnfsTer15 | c.3510delG | 0 | 1 | 1 | 0 | 0 | 3 | 0 | 0 | European |
|  | rs1356207947 | p.Gly1109GlufsTer13 | c.3321delA | 0 | 2 | 1 | 0 | 0 | 3 | 0 | 0 | Asian |
|  | rs770412732 | p.Gln1084ValfsTer21 | c.3250_3251delCA | 0 | 2 | 2 | 0 | 0 | 4 | 0 | 0 | European |
|  | rs138381300 | p.Ser761CysfSTer36 | c.2282_2285delCAGT | 2 | 4 | 4 | 1 | 0 | 8 | 0 | 0 | European, Asian, African American, Latino, |
|  | rs759739224 | p.Asp433HisfsTer43 | c.1297_1298delGA | 1 | 2 | 5 | 0 | 1 | 10 | 3 | 0 | European |
|  | rs763225328 | p.Ser417ValfsTer2 | c.1248dupG | 0 | 0 | 0 | 0 | 0 | 1 | 0 | 0 | Asian |
|  | rs781280495 | p.Ser249LysfsTer10 | c.745dupA | 2 | 1 | 0 | 0 | 1 | 1 | 0 | 0 | African American, European |
|  | rs370891418 | p.Gly221GlufsTer3 | c.660delA | 0 | 0 | 0 | 0 | 0 | 0 | 0 | 0 | Asian |
|  | rs769231084 | p.Asn186LysfsTer4 | c.557dupA | 1 | 0 | 0 | 0 | 1 | 0 | 1 | 0 | Ashkenazi Jewish, European, Latino |
|  | rs779619110 | p.Glu160ArgfsTer10 | c.477dupA | 2 | 1 | 1 | 0 | 1 | 1 | 1 | 0 | European, Asian |
|  | rs769677999 | p.Arg130GlufsTer63 | c.388_391delAGAA | 15 | 0 | 4 | 0 | 12 | 8 | 4 | 0 | African American |
| Rare | rs1395978363 | p.Ile8PhefsTer7 | c.21_22insTTTT | 0 | 0 | 0 | 1 | 0 | 0 | 0 | 0 | European |
| Rare | rs1168885071 | p.Ile8ThrfsTer6 | c.22_23insC | 0 | 0 | 1 | 1 | 0 | 0 | 0 | 0 | European |
| Rare | rs756605558 | p.Asp22ArgfsTer2 | c.63dupA | 0 | 0 | 0 | 0 | 0 | 0 | 0 | 0 | African American, European |
| Rare | rs774514736 | p.Asn66LysfsTer11 | c.198delC | 2 | 2 | 1 | 0 | 2 | 0 | 1 | 0 | African American |
| Rare | rs1195478941 | p.Ala84LeufsTer2 | c.250_254delGCATA | 0 | 0 | 0 | 0 | 0 | 0 | 0 | 0 | European |
| Rare | rs754930940 | p.Gln115ArgfsTer79 | c.344delA | 21 | 0 | 6 | 0 | 14 | 10 | 4 | 0 | Latino |
| Rare | rs1207524703 | p.Arg151GlyfsTer43 | c.441delA | 11 | 0 | 3 | 0 | 8 | 6 | 3 | 0 | Asian |
| Rare | rs945727472 | p.Gly149GlufsTer4 | c.441_442delAG | 1 | 0 | 0 | 0 | 1 | 0 | 0 | 0 | African American, Asian |
| Rare | rs914183074 | p.Arg151GlyfsTer43 | c.451delA | 12 | 0 | 3 | 0 | 8 | 6 | 3 | 0 | Ashkenazi Jewish |
| Rare | rs779619110 | p.Glu160LysfsTer34 | c.477delA | 9 | 0 | 3 | 0 | 6 | 3 | 3 | 0 | African American, Asian, European |
| Rare | rs778735196 | p.Glu172ValfsTer7 | c.515_516delAA | 2 | 1 | 0 | 0 | 1 | 0 | 0 | 0 | African American |
| Rare | rs755044971 | p.Asn176LysfsTer4 | c.527dupA | 1 | 1 | 0 | 0 | 0 | 0 | 0 | 0 | African American |
| Rare | rs749678415 | p.Glu208ThrfsTer15 | c.621_624delAGAA | 3 | 0 | 1 | 0 | 0 | 0 | 1 | 0 | European |
| Rare | rs1222586539 | p.Lys226AsnfsTer26 | c.678delA | 1 | 1 | 2 | 0 | 1 | 2 | 4 | 0 | European |
| Rare | rs753134537 | p.Ala243ProfsTer9 | c.727delG | 0 | 0 | 1 | 0 | 0 | 1 | 2 | 0 | Asian |
| Rare | rs140880252 | p.Lys255IlefsTer2 | c.762_766delCAAAA | 0 | 0 | 0 | 0 | 1 | 0 | 0 | 0 | Asian |
| Rare | rs778824842 | p.Tyr257LeufsTer15 | c.770delA | 2 | 0 | 0 | 0 | 1 | 6 | 0 | 0 | Asian |
| Rare | rs751629874 | p.Asn278LysfsTer168 | c.834delT | 6 | 7 | 19 | 2 | 2 | 42 | 7 | 0 | European |
| Rare | rs1400304361 | p.Glu286SerfsTer160 | c.856delG | 5 | 7 | 19 | 1 | 2 | 41 | 6 | 0 | Asian |
| Rare | rs1177012271 | p.His339MetfsTer107 | c.1015delC | 4 | 3 | 12 | 1 | 1 | 28 | 5 | 0 |  |
| Rare |  | p.Asp351Ter | c.1051_1118delGACAGCTCCAGACAATCAGGCACTCGTCACGCAGAGACTTCCTCTCGTGGACAGACTGCATCATCCCA | 0 | 0 | 0 | 0 | 0 | 0 | 0 | 0 | European |
| Rare | rs1432787549 | p.Asp393ThrfsTer53 | c.1177delG | 2 | 1 | 7 | 1 | 1 | 15 | 2 | 0 | Latino |
| Rare | rs779477624 | p.Ala405LeufsTer41 | c.1212delA | 1 | 1 | 5 | 1 | 1 | 12 | 1 | 0 | African American |
| Rare | rs200738705 | p.Ser423ArgfsTer23 | c.1269delT | 1 | 0 | 4 | 1 | 1 | 6 | 1 | 0 | Asian |
| Rare | rs939757086 | p.Thr487ProfsTer50 | c.1459delA | 2 | 2 | 7 | 0 | 0 | 10 | 3 | 0 | European |
| Rare | rs1325601352 | p.Asn537IlefsTer261 | c.1610delA | 9 | 12 | 32 | 1 | 1 | 69 | 10 | 0 | European |
| Rare | rs773623399 | p.Ser586ValfsTer5 | c.1754_1755insT | 0 | 2 | 1 | 0 | 0 | 1 | 0 | 0 | European |
| Rare | rs773623399 | p.His583ValfsTer5 | c.1747_1754delCACTCAGG | 0 | 2 | 1 | 0 | 0 | 2 | 0 | 0 | Asian |
| Rare | rs201705800 | p.Ser609TrpfsTer189 | c.1826delC | 7 | 11 | 25 | 1 | 1 | 49 | 6 | 0 | Asian |
| Rare | rs1184036708 | p.Thr682IlefsTer116 | c.2045delC | 5 | 9 | 14 | 1 | 0 | 29 | 5 | 0 | European |
| Rare | rs754037449 | p.Gly705GlufsTer92 | c.2114_2117delGAGA | 4 | 9 | 13 | 1 | 0 | 22 | 3 | 0 | Latino |
| Rare | rs543019854 | p.Gln713ProfsTer35 | c.2131_2137dupCGCCACC | 0 | 3 | 3 | 1 | 0 | 10 | 1 | 0 | Asian |
| Rare | rs1295742461 | p.Arg721AsnfsTer77 | c.2162delG | 4 | 6 | 11 | 1 | 0 | 18 | 3 | 0 | European |
| Rare | rs751648887 | p.Ser745ArgfsTer52 | c.2235_2238delTCAG | 3 | 5 | 7 | 1 | 0 | 12 | 2 | 0 | European, Asian |
| Rare | rs753053770 | p.Gly769ValfsTer29 | c.2306delG | 2 | 4 | 4 | 0 | 0 | 6 | 0 | 0 | European |
| Rare | rs750623625 | p.Leu794ProfsTer8 | c.2378dupT | 0 | 1 | 0 | 0 | 0 | 0 | 1 | 0 | European |
| Rare | rs761752767 | p.Ser798ArgfsTer19 | c.2394delC | 0 | 2 | 0 | 0 | 1 | 5 | 3 | 0 | European |
| Rare | rs1372655143 | p.Lys801SerfsTer15 | c.2402_2405delAACA | 0 | 2 | 0 | 0 | 1 | 4 | 2 | 0 | European |
| Rare | rs753652391 | p.Val816TrpfsTer9 | c.2442_2445dupTGGA | 1 | 1 | 1 | 0 | 0 | 1 | 0 | 0 | European |
| Rare | rs139207952 | p.Ser829PhefsTer35 | c.2485dupT | 3 | 3 | 1 | 0 | 0 | 7 | 1 | 0 | Latino |
| Rare | rs764191084 | p.Ser832GlufsTer36 | c.2477_2493dupGAGACAACTCCAGGCAC | 1 | 1 | 7 | 0 | 0 | 7 | 1 |  | Asian, Latino |
| Rare | rs536394626 | p.Ser847Ter | c.2540delC | 0 | 0 | 0 | 0 | 0 | 0 | 0 | 0 | African |
| Rare | rs1228964062 | p.Val861Ter | c.2581delG | 0 | 0 | 0 | 0 | 0 | 0 | 0 | 0 | European |
| Rare | rs748581094 | p.Ser923PhefsTer2 | c.2767dupT | 0 | 0 | 0 | 0 | 0 | 1 | 0 | 0 | Asian |
| Rare | rs1017307664 | p.Asp958ThrfsTer164 | c.2871delA | 4 | 11 | 19 | 1 | 0 | 41 | 3 | 0 | Asian |
| Rare | rs769977379 | p.Arg986GlyfsTer136 | c.2955delC | 4 | 9 | 16 | 1 | 0 | 34 | 3 | 0 | Asian |
| Rare | rs1347248170 | p.Arg992SerfsTer31 | c.2976_2977delAG | 0 | 4 | 2 | 2 | 0 | 8 | 2 | 0 | Asian |
| Rare | rs1336799597 | p.His997ThrfsTer125 | c.2988delG | 3 | 9 | 13 | 1 | 0 | 33 | 3 | 0 | European |
| Rare | rs779158407 | p.Arg1003AspfsTer119 | c.3006delC | 3 | 9 | 11 | 1 | 0 | 30 | 3 | 0 | European |
| Rare | rs753462313 | p.Arg1003AsnfsTer118 | c.3008_3011delGACA | 3 | 9 | 11 | 1 | 0 | 30 | 3 | 0 | Asian |
| Rare | rs1294946620 | p.Glu1011AspfsTer12 | c.3033_3034delGA | 0 | 0 | 1 | 1 | 0 | 4 | 1 | 0 | European |
| Rare | rs1355273525 | p.Ser1013ProfsTer109 | c.3037delT | 3 | 9 | 10 | 1 | 0 | 29 | 1 | 0 | Asian |
| Rare | rs1369947146 | p.Ser1069ArgfsTer52 | c.3207_3210delTCAG | 1 | 6 | 4 | 1 | 0 | 13 | 1 | 0 | European |
| Rare | rs760457700 | p.Glu1128CysfsTer53 | c.3381_3385delTGAGT | 3 | 3 | 4 | 2 | 0 | 9 | 5 | 0 | African American |
| Rare | rs1201611634 | p.Ala1130ProfsTer56 | c.3387delT | 2 | 2 | 7 | 0 | 0 | 9 | 4 | 0 | Asian, African American |
| Rare | rs542661380 | p.Gln1142LysfsTer44 | c.3424delC | 2 | 2 | 7 | 0 | 0 | 8 | 1 | 0 | Asian |
| Rare | rs766958359 | p.Ile1165PhefsTer21 | c.3492delC | 0 | 1 | 2 | 0 | 0 | 3 | 0 | 0 | Asian |
| Rare | rs759133376 | p.Gln1225AsnfsTer221 | c.3673delC | 9 | 14 | 30 | 3 | 1 | 61 | 5 | 0 | European |
| Rare | rs1224414518 | p.Lys1224AsnfsTer221 | c.3672_3675delACAA | 9 | 14 | 30 | 3 | 2 | 61 | 5 | 0 | European |
| Rare | rs1316013989 | p.Asp1245GlufsTer4 | c.3734dupA | 0 | 0 | 0 | 0 | 0 | 2 | 0 | 0 | African |
| Rare | rs1012054020 | p.Ser1260ProfsTer186 | c.3777delG | 8 | 14 | 25 | 3 | 1 | 51 | 5 | 0 | European |
| Rare | rs769028887 | p.Gln1266ArgfsTer180 | c.3796delC | 8 | 14 | 24 | 3 | 1 | 49 | 4 | 0 | Asian |
| Rare | rs781172022 | p.Phe1311SerfsTer135 | c.3930delG | 8 | 12 | 17 | 2 | 1 | 35 | 4 | 0 | European |
| Rare | rs764874345 | p.Thr1334ArgfsTer13 | c.4001_4002delCA | 0 | 1 | 1 | 1 | 0 | 5 | 1 | 0 | Latino, Asian |
| Rare | rs770008928 | p.Gln1341ArgfsTer105 | c.4020delA | 6 | 10 | 12 | 2 | 1 | 27 | 2 | 0 | European |
| Rare | rs1341142152 | p.Glu1355LysfsTer91 | c.4062delA | 6 | 10 | 12 | 1 | 1 | 23 | 2 | 0 | Asian |
| Rare | rs757562290 | p.Ser1398ArgfsTer47 | c.4194_4197delTGAG | 4 | 6 | 5 | 1 | 1 | 12 | 1 | 0 | European |
| Rare | rs757949828 | p.Gln1408ValfsTer41 | c.4222_4223delCA | 2 | 5 | 3 | 0 | 1 | 11 | 0 | 0 | African American |
| Rare | rs1274759123 | p.Lys1424ArgfsTer25 | c.4271_4272delAA | 2 | 1 | 2 | 0 | 1 | 8 | 0 | 0 | Asian |
| Rare | rs1237217711 | p.Arg1465AspfsTer45 | c.4393delA | 2 | 2 | 7 | 0 | 0 | 9 | 1 | 0 | African American |
| Rare | rs145675213 | p.Arg1469AlafsTer41 | c.4405delC | 1 | 2 | 7 | 0 | 0 | 8 | 1 | 0 | Asian |
| Rare | rs761352191 | p.Pro1493ArgfsTer17 | c.4478delC | 0 | 1 | 2 | 0 | 0 | 4 | 0 | 0 | European |
| Rare | rs375638840 | p.Gln1507HisfsTer3 | c.4521delA | 0 | 0 | 0 | 0 | 0 | 1 | 0 | 0 | European |
| Rare | rs762625752 | p.Gly1513AspfsTer193 | c.4538delG | 6 | 6 | 18 | 0 | 1 | 49 | 9 | 0 | European, African American |
| Rare | rs766298491 | p.Gln1525ProfsTer5 | c.4573dupC | 1 | 0 | 0 | 0 | 0 | 1 | 0 | 0 | European |
| Rare | rs746367318 | p.His1532MetfsTer174 | c.4594delC | 6 | 6 | 14 | 0 | 1 | 45 | 7 | 0 |  |
| Rare | rs1210469645 | p.Ser1570ThrfsTer136 | c.4709delG | 5 | 5 | 12 | 0 | 1 | 37 | 5 | 0 | European |
| Rare | rs764779863 | p.Ser1574ThrfsTer21 | c.4720_4721delTC | 1 | 1 | 2 | 1 | 1 | 6 | 1 | 0 | European |
| Rare | rs1369640633 | p.Asp1606ThrfsTer100 | c.4816delG | 4 | 5 | 7 | 0 | 0 | 26 | 4 | 0 | European |
| Rare | rs772690360 | p.Arg1609AlafsTer3 | c.4821_4822delTG | 0 | 0 | 0 | 0 | 0 | 0 | 0 | 0 | Asian |
| Rare | rs936961132 | p.Asn1632ThrfsTer74 | c.4895delA | 4 | 2 | 5 | 0 | 0 | 19 | 4 | 0 | Asian |
| Rare | rs771090956 | p.Gln1672ArgfsTer34 | c.5014delC | 2 | 2 | 3 | 0 | 0 | 9 | 2 | 0 | Asian |
| Rare | rs749542190 | p.Ser1675Ter | c.5024delC | 0 | 0 | 0 | 0 | 0 | 0 | 0 | 0 | Asian |
| Rare | rs1349082175 | p.Gly1678GlufsTer28 | c.5033delG | 2 | 2 | 3 | 0 | 0 | 7 | 2 | 0 | Asian |
| Rare | rs754076126 | p.Arg1684ProfsTer22 | c.5051delG | 1 | 2 | 3 | 0 | 0 | 6 | 2 | 0 | European |
| Rare | rs769874552 | p.Thr1693GlnfsTer13 | c.5076delC | 1 | 0 | 1 | 0 | 0 | 3 | 2 | 0 | Asian |
| Rare | rs768140964 | p.Gly1707LeufsTer12 | c.5118_5119insCTCA | 1 | 0 | 0 | 0 | 0 | 4 | 0 | 0 | Asian |
| Rare | rs1433030921 | p.Gly1707ThrfsTer62 | c.5119_5122delGGAG | 3 | 7 | 8 | 1 | 0 | 16 | 2 | 0 | Asian |
| Rare | rs780794588 | p.Ser1714ProfsTer56 | c.5139delG | 3 | 7 | 6 | 1 | 0 | 15 | 2 | 0 | Asian |
| Rare | rs1189669276 | p.Ser1733CysfsTer36 | c.5198_5201delCAGT | 2 | 5 | 3 | 1 | 0 | 8 | 1 | 0 | Asian, European |
| Rare | rs370736769 | p.His1779MetfsTer55 | c.5335delC | 2 | 2 | 6 | 0 | 0 | 10 | 3 | 0 | African |
| Rare | rs1194399233 | p.Gly1787GlufsTer47 | c.5360delG | 2 | 2 | 6 | 0 | 0 | 9 | 1 | 0 | European |
| Rare | rs1241835707 | p.Arg1791AspfsTer43 | c.5371delA | 1 | 2 | 6 | 0 | 0 | 9 | 1 | 0 | European |
| Rare | rs749905936 | p.Ile1813GlnfsTer21 | c.5436_5437insCAGGGTCCAC | 2 | 1 | 1 | 0 | 0 | 3 | 0 | 0 | European, Latino |
| Rare | rs765009981 | p.Ser1917ValfsTer178 | c.5748delC | 8 | 13 | 24 | 1 | 2 | 47 | 3 | 0 | European |
| Rare | rs755966032 | p.Ser1919ArgfsTer175 | c.5757_5760delCCAG | 8 | 13 | 24 | 1 | 2 | 46 | 3 | 0 | Asian |
| Rare | rs765411939 | p.Ser1924ArgfsTer170 | c.5772_5775delTCAG | 8 | 13 | 22 | 1 | 2 | 44 | 3 | 0 | European |
| Rare | rs548103791 | p.Arg1933SerfsTer162 | c.5799delG | 7 | 14 | 20 | 1 | 2 | 41 | 3 | 0 | African American |
| Rare | rs765230314 | p.Ser1939ProfsTer156 | c.5815delT | 7 | 13 | 20 | 1 | 2 | 39 | 3 | 0 | Asian |
| Rare | rs200569761 | p.Asn1941ThrfsTer154 | c.5822delA | 7 | 13 | 20 | 1 | 2 | 38 | 3 | 0 | Asian |
| Rare | rs113544881 | p.Gly1944AspfsTer151 | c.5827delC | 7 | 13 | 19 | 1 | 2 | 38 | 3 | 0 | European |

**Here, Ub= ubiquitin, FS= frameshift, WT= wildtype, K= lysine, S= serine, T= threonine C= cysteine.**

Table E1 continued

**Table E4. Degron motifs and stability of C-termini in recurrent pathogenic frameshift and nonsense mutants of FLG.**

| **Variant** | **Sequence of the added frameshift product** | **Novel C-termini degrons** | **Novel internal degrons in the added frameshift product** | **Machine Learning: affected C-termini stability** | **Affected C-termini Gravy index** |
| --- | --- | --- | --- | --- | --- |
| Wild type | N/A | N/A | N/A | 2.44 | -1.533 |
| p.His3951ProfsTer4 | PQF | None | None | 2.91 | -0.887 |
| p.Arg3272AsnfsTer118 | NQALVTQRLPLVDRLHHPMNRQDQVQERDTDPATSSQQTAPDTQAFRVDKLHLQSETVDTGGPVVVRPVIVRDIQKSQTHSQCQAMDRLGPISRATKSPHVTGQGEGLDVQGLSSTR | None | [AVP]x[ST][ST][ST] at 3303-3307 degron type SPOP | 3.24 | -0.707 |
| p.Ala3094HisfsTer37 | HKTAPGTQHPNTVRTPFVDTRGQAEEEGRGTTTSIR | SIR at 3127-3129; degron type APCC_TPR_1; recognized by APC/C E3 ligase. SIR degron is an experimentally validated degron for Q9UM13 (H.sapiens) at 183-185 positions. | None | 3.49 | -1.387 |
| p.Ser2649ValfsTer94 | VQEKDMDPTTSSQQTAPDTQALGTDKLHLQSETVDTEGTVVVRPVTMRDIQKTQTHSQCQPTDRLGPISRATKSPHVAGQGKRLDIQDLSSTR | None | [AVP]x[ST][ST][ST] at 2656-2660 degron type SPOP | 3.19 | -1.193 |
| p.Thr2496AsnfsTer104 | NPREGLMPPMGTQDPEVQADKLVTMNNQETAPGTQGRVTMKLPLGPTALDTRRWARDNQRGPGQAGTGDPVLARTVTVRDTQKTLRGGLGLLPETIMDLLRSS | RSx at 2596-2598; degron type R at -3. Peptides with this degron motif at their C-terminus have mean PSI of 2.238. Among them 27 (28.72%) are CRL substrates  RxS at 2596-2598; degron type R at -3. Peptides with this degron motif at their C-terminus have mean PSI of 2.384. Among them 25 (27.47%) are CRL substrates. | xRxxLxx[LIVM]x at 2580-2588; degron type APC/C (DBOX). | 1.92 | 0.64 |
| p.Gln2423ValfsTer2 | V | None | None | 2.82 | -0.327 |
| p.Ser2366ArgfsTer52 | RPVTVRDIQKTQTHSQCQPTDRLGPISRATKSPHVAGQQEGLDVQGLSSTR | None | None | 3.20 | -0.913 |
| p.His1897ProfsTer198 | PRRWARDNHQGPGQAGTRDPVLARTVTVRDTQKTLRGGLGLLPETILDLLGSSQEMAPDTLGPITKTEPVTGTLQTAPDNQALVTQSLPLVDRLRHPMNRQDQVQEKDMDPTTSSSQQTAPDTQALGMDKLHLQSETVDTEGTVVVRPVTVRDIQKTQTHSQCQHREKLGPISRATKSPHVASQGKALDVQGLSSTR | None | xRxxLxx[LIVM]x at 1931-1939; degron type APC/C (DBOX).  [AVP]x[ST][ST][ST] at 2007-2011 degron type SPOP  xRxxLxx[LIVM]x at 2061-2069; degron type APC/C (DBOX). | 3.03 | -0.613 |
| p.Glu1605ThrfsTer103 | TQKTLRGGLSRLPETIMDLLGSSQDMAPGTPGPIKKIEPVMGTLQRAPDNQALVMQRLPLVDRLHHPRNRQGQVQEKDMDPATSSQQTAPQTQALGADKIHL | None | xRxxLxx[LIVM]x at 1609-1617; degron type APC/C (DBOX).  [AVP]x[ST][ST][ST] at 1685-1689 degron type SPOP | 3.69 | -0.233 |
| p.Ser1595ArgfsTer110 | RTGTVRDTQKTLRGGLSRLPETIMDLLGSSQDMAPGTPGPIKKIEPVMGTLQRAPDNQALVMQRLPLVDRLHHPRNRQGQVQEKDMDPATSSQQTAPQTQALGADKIHL | None | xRxxLxx[LIVM]x at 1606-1614; degron type APC/C (DBOX).  [AVP]x[ST][ST][ST] at 1682-1686 degron type SPOP | 3.69 | -0.233 |
| p.Ser1235HisfsTer211 | HVTMKLPLGLTALDTHRWDRNNHRGPGQAGTRDPVLARTVTVRDTQTTPRGCLGLLPETIMDLLGSSQEMAPDTLGSIKKTEPVTGTLQTAPDNQALITQSLPLMDRLCHPMNRQDQVQEKDMDPATSSQQTAPDTQALGTDKLHLQSETVDTEGPVVVRSLTVRDIQKTQTHSQCQPTDKLGPISRATKSPHVASQGKALDVQGLSSTR | None | xRxxLxx[LIVM]x at 1606-1614; degron type APC/C (DBOX).  [AVP]x[ST][ST][ST] at 1682-1686 degron type SPOP | 3.03 | -0.613 |
| p.Ser1171GlnfsTer15 | QGEEEGRDPTMSNR | None | None | 3.69 | -2.16 |
| p.Gly1109GlufsTer13 | EGLDVQGLSSTR | None | None | 3.27 | -0.56 |
| p.Gln1084ValfsTer21 | VSVRPWTGWAPSAEPPRVRT | VxT at 1101-1103; degron type other. Peptides with this degron motif at their C-terminus have mean PSI of 2.294. Among them 0 (0%) are CRL substrates. | None | 2.75 | -0.927 |
| p.Ser761CysfsTer36 | CQAMDRLVTISRATKSPHVTGQGKGLDVQGLSSTR | None | None | 3.09 | -0.733 |
| p.Asp433HisfsTer43 | HTISVRPRKGWAETAEPPRVHTWPVRGTVWTFRVFPLPGEHS | None | None | 1.86 | -0.007 |
| p.Ser417ValfsTer2 | V | RGx at 415-417; degron type R at -3. Peptides with this degron motif at their C-terminus have mean PSI of 2.396. Among them 18 (29.03%) are CRL substrates. | None | 2.82 | -0.72 |
| p.Ser249LysfsTer10 | KSIRRKQNI | None | None | 3.34 | -1.567 |
| p.Gly221GlufsTer3 | EE | EE at 221-222; degron type -EE end. Peptides with this degron motif at their C-terminus have mean PSI of 2.623. Among them 39 (33.91%) are CRL substrates. | None | 3.39 | -2.3 |
| p.Asn186LysfsTer4 | KQD | None | None | 3.73 | -3.027 |
| p.Glu160ArgfsTer10 | RKKRIFTYS | None | None | 3.35 | -1.733 |
| p.Arg130GlufsTer63 | ETIEKGIREDPRAQEKQGGKGMNLVLKKKKEKDIHLLIEKKNMEKTIITQVKKRKTRLKILD | None | None | 3.40 | -0.813 |
| p.Lys4022Ter | - | AxG at 4019-4021; degron type -G end. Peptides with this degron motif at their C-terminus have mean PSI of 2.477. Among them 7 (10.61%) are CRL substrates.  AxxxG at 4017-4021; degron type -G end. Peptides with this degron motif at their C-terminus have mean PSI of 2.473. Among them 7 (10.14%) are CRL substrates.  AxxxG at 4016-4021; degron type -G end. Peptides with this degron motif at their C-terminus have mean PSI of 2.589. Among them 10 (16.39%) are CRL substrates.  SxxG at 4018-4021; degron type -G end. Peptides with this degron motif at their C-terminus have mean PSI of 2.491. Among them 9 (12.68%) are CRL substrates. | N/A | 2.15 | -0.06 |
| p.Arg3879Ter | - | None | N/A | 3.73 | -2.22 |
| p.Gln3859Ter | - | RxxR at 3855-3858; degron type -R end. Peptides with this degron motif at their C-terminus have mean PSI of 2.792. Among them 9 (7.96%) are CRL substrates. | N/A | 3.44 | -2.073 |
| p.Gln3818Ter | - | EE at 3816-3817; degron type -EE end. Peptides with this degron motif at their C-terminus have mean PSI of 2.623. Among them 39 (33.91%) are CRL substrates. | N/A | 3.19 | -2.233 |
| p.Ser3749Ter | - | None | N/A | 3.48 | -2.627 |
| p.Arg3743Ter | - | QA at 3741-3742; degron type -A end. Peptides with this degron motif at their C-terminus have mean PSI of 2.442. Among them 2 (2.47%) are CRL substrates. | N/A | 3.44 | -1.76 |
| p.Gln3684Ter | - | AxG at 3681-3683; degron type -G end. Peptides with this degron motif at their C-terminus have mean PSI of 2.477. Among them 7 (10.61%) are CRL substrates.  SxxG at 3680-3683; degron type -G end. Peptides with this degron motif at their C-terminus have mean PSI of 2.491. Among them 9 (12.68%) are CRL substrates.  SxxxxG at 3678-3683; degron type -G end. Peptides with this degron motif at their C-terminus have mean PSI of 2.474. Among them 17 (20.0%) are CRL substrates.  VxxxG at 3679-3683; degron type -G end. Peptides with this degron motif at their C-terminus have mean PSI of 2.279. Among them 3 (5.0%) are CRL substrates. | N/A | 3.60 | -1.273 |
| p.Arg3657Ter | - | None | N/A | 3.47 | -0.613 |
| p.Gln3520Ter | - | RxS at 3517-3519; degron type R at -3. Peptides with this degron motif at their C-terminus have mean PSI of 2.384. Among them 25 (27.47%) are CRL substrates. | N/A | 2.99 | -1.873 |
| p.Arg3442Ter | - | None | N/A | 3.73 | -1.54 |
| p.Arg3419Ter | - | QA at 3417-3418; degron type -A end. Peptides with this degron motif at their C-terminus have mean PSI of 2.442. Among them 2 (2.47%) are CRL substrates. | N/A | 3.43 | -2.16 |
| p.Arg3409Ter | - | RxxxG at 3404-3408; degron type -G end. Peptides with this degron motif at their C-terminus have mean PSI of 2.34. Among them 28 (34.57%) are CRL substrates.  SxG at 3406-3408; degron type -G end. Peptides with this degron motif at their C-terminus have mean PSI of 2.515. Among them 8 (8.7%) are CRL substrates. | N/A | 2.55 | -1.747 |
| p.Ser3316Ter | - | None | N/A | 3.51 | -2.36 |
| p.Ser3296Ter | - | AR at 3294-3295; degron type A at -2. Peptides with this degron motif at their C-terminus have mean PSI of 2.376. Among them 5 (8.33%) are CRL substrates. | N/A | 3.23 | -1.167 |
| p.Ser3247Ter | - | None | N/A | 3.50 | -1.613 |
| p.Gln3029Ter | - | None | N/A | 3.66 | -2.013 |
| p.Arg3009Ter | - | None | N/A | 3.47 | -0.613 |
| p.Arg2971Ter | - | QA at 2969-2970; degron type -A end. Peptides with this degron motif at their C-terminus have mean PSI of 2.442. Among them 2 (2.47%) are CRL substrates. | N/A | 3.53 | -1.1 |
| p.Trp2907Ter | - | None | N/A | 3.77 | -2.44 |
| p.Ser2706Ter | - | None | N/A | 3.65 | -2.193 |
| p.Arg2685Ter | - | None | N/A | 3.47 | -0.613 |
| p.Arg2613Ter | - | None | N/A | 3.70 | -2.48 |
| p.Ser2554Ter | - | None | N/A | 3.51 | -1.34 |
| p.Ser2544Ter | - | None | N/A | 3.63 | -1.76 |
| p.Ser2453Ter | - | None | N/A | 3.41 | -2.567 |
| p.Arg2447Ter | - | QA at 2445-2446; degron type -A end. Peptides with this degron motif at their C-terminus have mean PSI of 2.442. Among them 2 (2.47%) are CRL substrates. | N/A | 3.41 | -1.64 |
| p.Glu2422Ter | - | None | N/A | 2.70 | -0.727 |
| p.Gln2417Ter | - | None | N/A | 3.24 | -0.747 |
| p.Gln2397Ter | - | None | N/A | 3.76 | -1.14 |
| p.Arg2361Ter | - | None | N/A | 3.47 | -0.613 |
| p.Ser2344Ter | - | None | N/A | 3.54 | -2.273 |
| p.Ser2317Ter | - | AA at 2315-2316; degron type -A end. Peptides with this degron motif at their C-terminus have mean PSI of 2.193. Among them 7 (9.46%) are CRL substrates.  QxA at 2314-2316; degron type -A end. Peptides with this degron motif at their C-terminus have mean PSI of 2.523. Among them 2 (3.92%) are CRL substrates. | N/A | 3.12 | -1.053 |
| p.Gly2228Ter | - | None | N/A | 2.85 | -0.56 |
| p.Tyr2092Ter | - | None | N/A | 3.72 | -1.0 |
| p.Ser2080Ter | - | ARxx at 2076-2079; degron type R at -3. Peptides with this degron motif at their C-terminus have mean PSI of 2.388. Among them 11 (19.64%) are CRL substrates.  RGx at 2077-2079; degron type R at -3. Peptides with this degron motif at their C-terminus have mean PSI of 2.396. Among them 18 (29.03%) are CRL substrates. | N/A | 2.97 | -1.947 |
| p.Gln2070Ter | - | None | N/A | 3.88 | -1.2 |
| p.Arg2037Ter | - | None | N/A | 3.47 | -0.613 |
| p.Ser1977Ter | - | None | N/A | 3.52 | -1.933 |
| p.Trp1947Ter | - | None | N/A | 3.24 | -1.113 |
| p.Ser1906Ter | - | None | N/A | 3.46 | -1.613 |
| p.Gly1826Ter | - | None | N/A | 3.21 | -1.647 |
| p.Arg1798Ter | - | QA at 1796-1797; degron type -A end. Peptides with this degron motif at their C-terminus have mean PSI of 2.442. Among them 2 (2.47%) are CRL substrates. | N/A | 3.49 | -2.033 |
| p.Glu1795Ter | - | None | N/A | 3.32 | -2.06 |
| p.Gln1790Ter | - | None | N/A | 3.18 | -1.393 |
| p.Ser1733Ter | - | None | N/A | 3.76 | -2.013 |
| p.Ser1729Ter | - | EE at 1727-1728; degron type -EE end. Peptides with this degron motif at their C-terminus have mean PSI of 2.623. Among them 39 (33.91%) are CRL substrates. | N/A | 3.42 | -1.633 |
| p.Gly1724Ter | - | None | N/A | 3.78 | -1.513 |
| p.Arg1712Ter | - | None | N/A | 3.53 | -1.26 |
| p.Gln1701Ter | - | None | N/A | 3.53 | -1.773 |
| p.Ser1695Ter | - | None | N/A | 3.76 | -2.127 |
| p.Ser1515Ter | - | None | N/A | 3.40 | -1.98 |
| p.Arg1474Ter | - | QA at 1472-1473; degron type -A end. Peptides with this degron motif at their C-terminus have mean PSI of 2.442. Among them 2 (2.47%) are CRL substrates. | N/A | 3.58 | -1.613 |
| p.Ser1302Ter | - | REx at 1299-1301; degron type R at -3. Peptides with this degron motif at their C-terminus have mean PSI of 2.53. Among them 20 (39.22%) are CRL substrates. | N/A | 3.47 | -1.927 |
| p.Gln1256Ter | - | None | N/A | 3.38 | -1.227 |
| p.Gly1253Ter | - | None | N/A | 3.17 | -1.06 |
| p.Arg1140Ter | - | RxxxG at 1135-1139; degron type -G end. Peptides with this degron motif at their C-terminus have mean PSI of 2.34. Among them 28 (34.57%) are CRL substrates.  SxG at 1137-1139; degron type -G end. Peptides with this degron motif at their C-terminus have mean PSI of 2.515. Among them 8 (8.7%) are CRL substrates. | N/A | 2.55 | -1.747 |
| p.Gly1139Ter | - | None | N/A | 2.83 | -1.933 |
| p.Ser1040Ter | - | None | N/A | 3.69 | -2.227 |
| p.Ser1020Ter | - | AA at 1018-1019; degron type -A end. Peptides with this degron motif at their C-terminus have mean PSI of 2.193. Among them 7 (9.46%) are CRL substrates.  QxA at 1017-1019; degron type -A end. Peptides with this degron motif at their C-terminus have mean PSI of 2.523. Among them 2 (3.92%) are CRL substrates. | N/A | 3.19 | -0.76 |
| p.Gln977Ter | - | None | N/A | 3.58 | -1.6 |
| p.Arg826Ter | - | QA at 824-825; degron type -A end. Peptides with this degron motif at their C-terminus have mean PSI of 2.442. Among them 2 (2.47%) are CRL substrates. | N/A | 3.37 | -1.367 |
| p.Arg788Ter | - | None | N/A | 3.31 | -2.4 |
| p.Arg740Ter | - | None | N/A | 3.52 | -0.96 |
| p.Ser609Ter | - | None | N/A | 3.49 | -1.547 |
| p.Arg501Ter | - | QA at 499-500; degron type -A end. Peptides with this degron motif at their C-terminus have mean PSI of 2.442. Among them 2 (2.47%) are CRL substrates. | N/A | 3.67 | -1.613 |
| p.Gln355Ter | - | None | N/A | 3.68 | -1.96 |
| p.Trp326Ter | - | None | N/A | 3.51 | -1.733 |
| p.Ser260Ter | - | None | N/A | 3.33 | -1.387 |
| p.Lys182Ter | - | None | N/A | 3.89 | -2.633 |

**Table E5: List of S100 proteins and S100 fused-type proteins & their ubiquitin conjugating amino acid content.**

| **Protein** | **Ubquitin-conjugating amino acid** | | | | **Total number of amino acids (aa)** |
| --- | --- | --- | --- | --- | --- |
|  | **Lysine** | **Serine** | **Threonine** | **Cysteine** |  |
| S100A1 | 9 | 5 | 4 | 1 | 94 |
| S100A2 isoform 1 | 9 | 7 | 3 | 4 | 98 |
| S100A2 isoform 2 | 9 | 8 | 3 | 4 | 96 |
| S100A2 isoform 3 | 5 | 3 | 1 | 2 | 64 |
| S100A3 | 7 | 4 | 5 | 10 | 101 |
| S100A4 | 12 | 7 | 3 | 4 | 101 |
| S100A5 | 12 | 8 | 7 | 2 | 92 |
| S100A6 | 9 | 3 | 3 | 1 | 90 |
| S100A7 | 12 | 9 | 5 | 2 | 101 |
| S100A7A | 12 | 9 | 4 | 2 | 101 |
| S100A8 | 14 | 8 | 3 | 2 | 117 |
| S100A9 | 11 | 4 | 7 | 1 | 114 |
| S100A10 | 12 | 3 | 4 | 2 | 97 |
| S100A11 | 10 | 9 | 8 | 2 | 105 |
| S100A12 | 10 | 3 | 5 | 0 | 92 |
| S100A13 | 13 | 6 | 6 | 0 | 98 |
| S100A14 | 6 | 9 | 4 | 3 | 104 |
| S100A16 | 10 | 11 | 4 | 1 | 103 |
| S100B | 8 | 4 | 3 | 2 | 92 |
| S100G | 11 | 6 | 2 | 0 | 79 |
| S100P | 10 | 7 | 6 | 1 | 95 |
| S100Z | 12 | 3 | 5 | 2 | 99 |
| Trichohyalin like protein 1* | 4 | 8 | 4 | 3 | 100 |
| Trichohyalin* | 6 | 4 | 4 | 3 | 100 |
| Cornulin* | 5 | 3 | 7 | 3 | 100 |
| Repetin* | 6 | 4 | 3 | 3 | 100 |
| Filaggrin 2* | 11 | 5 | 7 | 3 | 100 |
| Hornerin* | 10 | 3 | 5 | 1 | 100 |
| **Filaggrin*** | **14** | **5** | **5** | **0** | **100** |

*N-terminal domain (1 to 100 aa) of S100 fused-type proteins.

**Table E6: List of degron motifs present in S100 fused-type proteins.**

| **Protein (Uniprot ID)** | **Degron motif** | **Degron type** | **Indices** |
| --- | --- | --- | --- |
| Trichohyalin like protein 1 (Q5QJ38) | MPx or M{0,1}[GP]x | Pro/N degron or Ac/N degron | 1-3 or 1-3 |
|  | xRxxLxx[LIVM]x | APC/C (DBOX) | 5-13 |
|  | xx[DE]x(T)PxK | SCF_SKP2-CKS1_1 | 339-346 |
|  | [DNS]x[DES][TNS]GE | Kelch_KEAP1_1 | 561-566 |
|  | xPxAxVxP[^P] | SIAH | 642-650 |
| Trichohyalin (Q07283) | MSPx or M{0,1}[AST]x | Pro/N degron or Ac/N degron | 1-4 or 1-3 |
|  | xRxxLxx[LIVM]x | APC/C (DBOX) | 211-219 |
|  | xKENx | APC/C (KEN) | 1230-1234 |
| Cornulin (Q9UBG3) | MPx or M{0,1}[GP]x | Pro/N degron or Ac/N degron | 1-3 or 1-3 |
|  | xRxxLxx[LIVM]x | APC/C (DBOX) | 29-37 |
| Repetin (Q6XPR3) | M{0,1}[AST]x | Ac/N degron | 1-3 |
| Filaggrin 2 (Q5D862) | M{0,1}[AST]x | Ac/N degron | 1-3 |
|  | [DNS]x[DES][TNS]GE | Kelch_KEAP1_1 | 216-221 |
|  | D(S)Gx{2,3}([ST]) | SCF-TRCP1 | 272-277 |
|  | D(S)Gx{2,3}([ST]) | SCF-TRCP1 | 1198-1203 |
| Hornerin (Q86YZ3) | MPx or M{0,1}[GP]x | Pro/N degron or Ac/N degron | 1-3 or 1-3 |
|  | [AVP]x[ST][ST][ST] | SPOP | 511-515 |
|  | [AVP]x[ST][ST][ST] | SPOP | 2832-2836 |


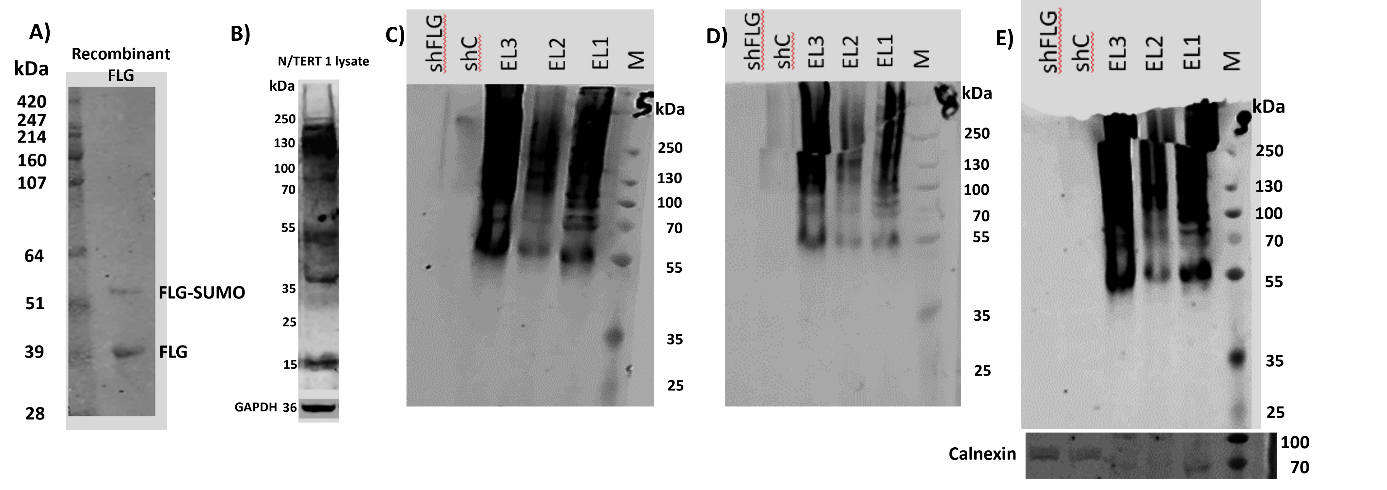


**Figure E1: Validation of the anti-filaggrin FLG01 monoclonal antibody.** A) Western blot with recombinant filaggrin expressed with SUMO tag (gift from Dr. Ewa Podobas and Prf. Wojciech Bal); blotted with FLG01; B) N/TERT1 cell lysate assayed with FLG01; C-D) comparison of FLG01 with other anti-filaggrin antibodies; filaggrin knock down HaCaT keratinocytes (shC, control cells; shFLG knock down cells); EL1-3: epidermal lysates (from 3 separate donors); membranes blotted with FLG01(C), SPM181(D) and AKH1(E).


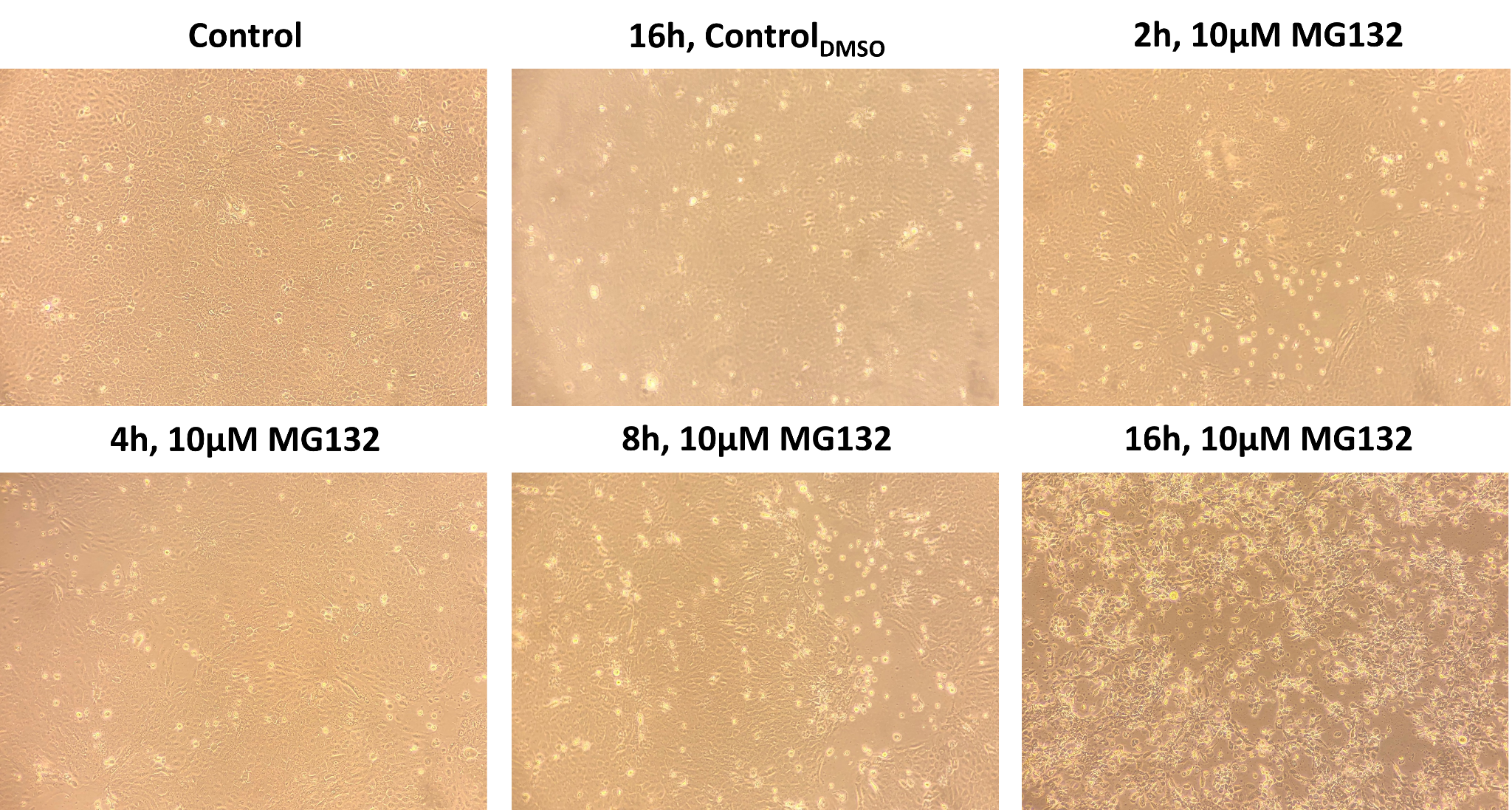
**Figure E2: Appearance of the N/TERT-1 keratinocytes upon treatment with the proteasome inhibitor MG132.** Cells in the control panel were treated with only media where as 16h control DMSO cells were treated with same volume of DMSO as of the solvent of 10µM MG132, Other time point represent the incubation time of 10µM MG132.

1. Trichohyalin like protein 1 mpqLlrnvlcvieTFH**K**YASEDSNGATLTGREL**K**QLIQGEFGDFFQ-PCVLHAVE**K**NSNL 59

2. Trichohyalin msplLRSICDITEIFNQYVSHDCDGAALT**KK**DL**K**NLLEREFGAVLRRPHDP**K**TVDLILEL 60

3. Cornulin mpqLLQNINGIIEAFRRYARTEGNCTALtrgel**k**rllEQEFADVIV**K**PHDPATVDEVLRL 60

4. Repetin maqLLNSILSVIDVFH**K**YA**K**GNGDCALLC**K**EEL**K**QLLLAEFGDILQRPNDPETVETILNL 60

5. Filaggrin 2 mtdLLRSVVTVIDVFY**K**YT**K**QDGECGTLS**K**GEL**K**ELLE**K**ELHPVL**K**NPDDPDTVDVIMHM 60

6. Hornerin mp**k**LLQGVITVIDVFYQYATQHGEYDTLN**K**AEL**K**ELLENEFHQIL**K**NPNDPDTVDIILQS 60

7. Filaggrin mstLLENIFAIINLF**K**QYS**KK**D**K**NTDTLS**KK**EL**K**ELLE**K**EFRQIL**K**NPDDPDMVdvfmdh 60

* ** * * * ** * * * *

1. Trichohyalin like protein 1 LNIDSNGIISFDEFVLAIFNLLNLCYLDI**K**SLLSS--ELRQVT**K**PE**K**E**K**LDDVDVQATTG 117

2. Trichohyalin LDLDSNGRVDFNEFLLFIF**K**VAQACYYALGQATGLDEE**K**RARCDG**K**ESLLQDRRQE-EDQ 119

3. Cornulin LDEDHTGTVEF**K**EFLVLVF**K**VAQACF**K**TLSESAEG------ACGSQESGSLHSGAS-QE- 112

4. Repetin LDQDRDGHIDFHEYLLLVFQLVQACYH**K**LDN**K**SHG------GRTSQQERGQEGAQD---- 110

5. Filaggrin 2 LDRDHDRRLDFTEFLLMIF**K**LTMACN**K**VLS**K**E-----------------------Y---- 93

6. Hornerin LDRDHN**KK**VDFTEYLLMIF**K**LVQARN**K**IIG**K**D-----------------------Y---- 93

7. Filaggrin ldIDHN**KK**IDFTEFLLMVF**K**LAQAYYESTr**k**enlPISGH**K**HR**K**HSHHD**K**HEDN**K**QE-En**k** 119

* * * * *

1. Trichohyalin like protein 1 DGQWTVGTSPTQE**K**RMLPSGMAS-SSQLIP---------EESGAVGNN----------RV 157

2. Trichohyalin -RRFEPRDRQL-EEE---PGQRR-R-Q**K**RQEQERELAE-GEEQSE**K**QERLEQRDRQRRDE 171

3. Cornulin -----LGE----GQR---SGTEVGRAG**K**GQHYEGSSHRQSQQGSRGQNRPGV------QT 154

4. Repetin ------------C**K**F---PGNTG-RQHRQRHEE------ERQNSHHSQ---P------ER 139

5. Filaggrin 2 ------------C**K**A---SGS**KK**-HRRGHRHQE------EESETEEDEEDTP------GH 125

6. Hornerin ------------CQV---SGS**K**L-RDDTHQHQE------EQEETE**K**E--EN**K**------RQ 123

7. Filaggrin enr**K**RP--SSL-ERRNNR**K**GN**K**-GRS**K**SPRETGG**K**R---HESSSE**KK**ER**K**GYSP-THREE 171

*

1. Trichohyalin like protein 1 DPWREA**K**THNFPGEASEHNDP**K**N**K**HLEGDEQSQEVAQDIQTTEDNEGQL**K**TN**K**PMAGS**KK** 217

2. Trichohyalin ELWRQRQ--EWQE--REERRAEEEQLQSC------**K**GHETEEFPDEEQLrrrell----- 216

3. Cornulin QGQATGS--AWVS--SYDRQAESQSQERI-------------SP---------------- 181

4. Repetin QDGDSHH--GQPE--RQDRDSHHGQSE**K**Q------------------------------- 164

5. Filaggrin 2 **K**SGYRHS--SWSE--GEE----HGYSSGH------------------------------- 146

6. Hornerin ESSFSHS--SWSA--GEN----DSYSRNV------------------------------- 144

7. Filaggrin EYG**K**NHH--NSS**K**--**K**E**K**N**K**TENTRLGDN----------------------R**K**RL----- 200

**Figure E3: Amino acid sequence alignment of S100 fused-type protein family.** Lysine (**K**), serine (S), threonine (T) and cysteine (C) residues are considered as conserved if shared by at least two proteins (marked as green), asterisk indicate amino acids which are conserved in all S100 fused type proteins. Degron motifs are in lowercase (red).

1. Trichohyalin like protein 1 TSSPTER**K**GQD**K**EISQEGDEPAR---EQSVS**K**IRDQFGEQEGNLATQSSPP**K**EATQRPCE 274

2. Trichohyalin ---elrR**K**GREE**K**QQQRRERQDRVFQE----EEE**K**EWR-**K**RETVLR**K**EEE**K**L-QEEEPQR 267

3. Cornulin ---QIQLSGQTEQTQ**K**-A---------------------GEG**K**R-N----QT-TEMRPER 210

4. Repetin ---------------------------------------DRDSH-H---------SQPER 175

5. Filaggrin 2 ---------------------------------------SRGTV-**K**----CR-HGSNSRR 161

6. Hornerin ----------------------------------------RGSL-**K**----PG-TESISRR 158

7. Filaggrin ---S-------ERLEE**K**EDNEEGVYDYENTGRMTQ**K**WI-QSGHIATYY--TI-QDEAYDT 246

1. Trichohyalin like protein 1 DQEVRTE**K**E**K**HSNIQEPPLQREDEPSSQHA-DLP--------EQAAARSPSQTQ**K**STDS**K** 325

2. Trichohyalin QRELQEEEEQLR**K**LERQELRRERQEEEQQQQRLRREQQLRR**K**QEEERREQQEERREQQER 327

3. Cornulin Q----------------PQTREQDRAHQTGETVTGSG----------------------- 231

4. Repetin Q--------------------DRDSHHNQSER---------------------------- 187

5. Filaggrin 2 L----------------GRQGNLSSSGNQEG----------------------------- 176

6. Hornerin L----------------SFQRDFSGQHNSYSG---------------------------- 174

7. Filaggrin TDSLLEEN**K**I--------YERSRSSDG**K**SSSQVNRSRH-----ENTSQVPLQESRTR**K**RR 293

1. Trichohyalin like protein 1 --DVCRMFDTQEPG**K**dadqt--pa**k**T----**K**NLGEPEDYGRTSETQE**K**ECE-------T**K** 370

2. Trichohyalin REQQEERREQQLRREQEERREQQLRREQEEERREQQLRREQEEERREQQLRREQQLRREQ 387

3. Cornulin --TQTQAGATQ-TVEQDSSH-Q-TGRTS**K**Q---------TQ-EATND------------- 263

4. Repetin --QD**K**DFSFD-----QSE-------RQSQD------------------------------ 203

5. Filaggrin 2 --SQ**K**RY-----------------HRSSCGHSW--------------------------- 190

6. Hornerin --QSSSYGEQ-----NSDSH-QSSGRGQCGSGSGQSPNYGQHGSGSG------------- 213

7. Filaggrin GSRVSQDRDSEGHSEDSERHSGSASRNHHGSAWEQSRDGSRHPRSHDEDRAS-HGH--SA 350

1. Trichohyalin like protein 1 DLPVQYGSRNGSE---TSDMRDER**K**--------------ERRGPEAHGTAGQ**K**ERDR**K**TR 413

2. Trichohyalin QLRREQQLRREQQLRREQQLRREQQLRREQQLRREQQLRREQEEERHEQ**K**HEQERRE--- 444

3. Cornulin ---------------------------------------QNRGTETHGQGRSQTS----- 279

4. Repetin -----------------------------------------------------SS----- 205

5. Filaggrin 2 ----------------------------------------SGG**K**DRHGSSSVELR----- 205

6. Hornerin ---------------------------------------QSSSNDTHGSGSGQSS----- 229

7. Filaggrin DSSRQSG------------TRHaetssRGQ-TASSHEQARSSPGERHGSGHQQSADS--- 394

**Figure E3 continued.**

1. Trichohyalin like protein 1 PLVLETQTQDG**K**YQELQGLS**K**S**K**DAE**K**GSETQYLSSEGGDQTHPELEGTAVSGEEAE--- 470

2. Trichohyalin ----------------QRL**K**REQEE----RRDWL**K**REEETERHEQERR**K**QQL**K**RDQEEE- 483

3. Cornulin ----------------QAVTG--------------------GH----------------- 286

4. Repetin ----------------SG**KK**V--------------------SH**K**S------------Ts- 216

5. Filaggrin 2 ----------------ERIN**K**--------------------SHISPsr-esgeEYESGS- 227

6. Hornerin -----------------GFSQ---------------------H**K**SSSG-QSSGYSQHGS- 249

7. Filaggrin ----------------SRHSATGR-------GQASSAVSDRGHRGSSGSQASDSEGHSEN 431

1. Trichohyalin like protein 1 --------HT**K**E----------------------------GTAEAFVNS**K**--NAP----- 487

2. Trichohyalin -RRERWL**K**LEEEERREQQERREQQLRREQEERREQRL**K**RQEEEERLQQRLRSEQQLRREQ 542

3. Cornulin -------AQIQAGTHTQTPTQT--VEQD-------SSHQTGSTSTQTQ-ES--------- 320

4. Repetin -GQA**K**WQGHIFALNRCE**K**PIQD--SHYGQSERHTQQSETLGQASHFNQ------------ 261

5. Filaggrin 2 -GSNSWER**K**GHGGLSCGLETS-----------GHESNSTQ---------SR--------- 257

6. Hornerin -GSGHSSGYGQHGSRSGQSSR-----------GERHRSSSGSSSSYGQHGS--------- 288

7. Filaggrin SDTQSVSGHG**K**AGLRQQSHQE---STRGR---SGERSGRSG--SSLYQVSTHEQPD-S-- 480

1. Trichohyalin like protein 1 --AAERTLGARE---------RTQDLA--------PLE**K**QSVGE--NTRVT**K**THDQPVEE 526

2. Trichohyalin EERREQLL-**K**REEE**K**RLEQERREQRL**K**REQEERRDQLL**K**REEER-RQQRL**K**REQEERLEQ 600

3. Cornulin --T------------------NGQNRGTEIH--------GQ-GR-SQ------------- 337

4. Repetin --T------------------NQQ**K**SGSYCG--------QS--E-R-----------LGQ 279

5. Filaggrin 2 --I------------------REQ**K**LGSSCS--------GS-Gd-sgr---rsHACGYSN 284

6. Hornerin --G------------------SRQSLGHGRQ--------GS-GS-RQS---PSH---VRH 312

7. Filaggrin AHGRTGTSTGGRQGSHHEQARDSSRHS-ASQEGQDT-IRGHPGSSRGGRQGSHHEQSV-N 537

1. Trichohyalin like protein 1 EDGY----------------QGEDPESPFTQSDEGSSETPNSLASEEGNSssetgeLPVQ 570

2. Trichohyalin RL**K**REEVERLEQEERREQRL**K**REEPEEERR---QQ------LL**K**SEE----------QEE 641

3. Cornulin ------------------------------------------------------------ 337

4. Repetin ELGC------------------GQTDRQG-----Q------SSHYG-----------QTD 299

5. Filaggrin 2 SSGC------------------GRPQNAS-----S------SCQSH-----------RFG 304

6. Hornerin GSGS------------------------------G------HSSSH-----------GQH 325

7. Filaggrin RSGHS-----------------------------G------SHHSHT----------TSQ 552

**Figure E3 continued.**

1. Trichohyalin like protein 1 GDSQSQGDQHGESVQGGHNNNPDTQRQGTPGE**K**NRALEAVVPAVRGEDVQLTED------ 624

2. Trichohyalin RRQQQLRREQQERREQRL**K**REEEEERLEQRL--**K**REHEEE-----RREQELAEEE----- 689

3. Cornulin ---TSQAVTGGHTQIQAG-SHTET---------------------------VEQ------ 360

4. Repetin RQDQSYHYGQTDRQGQSS-HYSQTDRQGQ------------------SSHYSQP------ 334

5. Filaggrin 2 GQGNQFSYIQSGCQS--------------------------------------------- 319

6. Hornerin GSGSSYSYSRGHYES--------------------------------------------- 340

7. Filaggrin GRSDASHGQSGSRSASRQTRNEEQSGDGTRHSGSRHHEASSQADSSRHSQVGQGQSSGPR 612

1. Trichohyalin like protein 1 --------------------------------------------QEQPARGEH**K**NQGPGT 640

2. Trichohyalin --------QEQARERI**K**SRIP**K**WQWQLESEADAR-----QS**K**-VYSRPR**K**QEGQRRRQEQ 735

3. Cornulin -------------DRSQT-VSHG---GARE---------------------------QGQ 376

4. Repetin -------------DRQGQ-SSHY---GQMDR-**K**G-----QCY-HYDQTN-----RQGQGS 365

5. Filaggrin 2 --------------GI**K**G--------------------------------------GQGH 327

6. Hornerin --------------GSGQ-TSGF---GQHESGSG-----QSS-GYS**K**HGSGSGHSSSQGQ 376

7. Filaggrin TSRNQGSSVSQDSDSQGHSEDSERWSGSASRNHHGSAQEQSRDGSRHPRSHHEDRAGHGH 672

1. Trichohyalin like protein 1 **K**--g----pgaavepnGHPEAQESTAGDENR**K**SLEIEITGALDEDFTDQLSLMQ---LPG 691

2. Trichohyalin EE**K**RRRRESELQWQEEERAH-------------RQQQE-EEQRRDFTWQWQAEE-**K**SERG 780

3. Cornulin T--QTQPGSGQRWMQVSNPEAGE------TVPGGQAQT-GASTESGRQEWSSTH-P--RR 424

4. Repetin H--YSQPNRQGQSSHYGQPDTQD-----QSSHYGQTDR-QDQSSHYGQTERQG---QSSH 414

5. Filaggrin 2 G--C---VSGGQPSGCGQPESNPCSQSYSQRGYGAREN-GQPQN-CGGQWRTGS-SQSSC 379

6. Hornerin H--G---STSGQASSSGQHGS----SSRQSSSYGQHES-ASRHSSGRGQHSSGS-GQSPG 425

7. Filaggrin SADSSR-**K**S-------GTRHTQNSSSGQAASSHEQARSSAGERHGSRHQLQSADSSRHSG 724

1. Trichohyalin like protein 1 **K**G--DSRNEL**K**VQGPSS**K**EE**K**GRATEAQNTLLESLDEDNSASL**K**IQLET**K**EPVTSEEEDE 749

2. Trichohyalin RQRLSARPPLREQRERQLRAE-ERQQREQRFLPEEEE**K**EQR----------RRQRRERE**K** 829

3. Cornulin CV---------------------------------------------------------- 426

4. Repetin YSQ--------------------------------------------------------- 417

5. Filaggrin 2 CG---------------------------------------------------------- 381

6. Hornerin HG---------------------------------------------------------- 427

7. Filaggrin TGHGQASSAVRdsghrgsSGS-QATD-------SEGHS-------------------EDS 757

**Figure E3 continued**

1. Trichohyalin like protein 1 SPQELAGEGG-------------------DQ**K**SPA**KK**EH--NSSVPWSSLE**K**QMQRDQEP 788

2. Trichohyalin ELQFLEEEEQLQRRERAQQLQEEEDGLQEDQERRRSQEQRRDQ**K**WRWQLEEER------- 882

3. Cornulin ---------------------T--EGQG------DRQPTVVGEEWVDDHS--R------- 448

4. Repetin -------------------MDR--QGQG------S---------HYGQTD--R------- 432

5. Filaggrin 2 ---------------------Q--YGSG------GSQSCSNGQHEYGSCG--R------- 403

6. Hornerin ---------------------Q--RGSG------SGQSPSSGQHGTG------------- 445

7. Filaggrin DTQSVSGHGQ------AG---HHQQSHQESARDRSGERSRRSGSFLYQVSTH**K**------- 801

1. Trichohyalin like protein 1 CSVERGAVYSSPLYQYLQE**K**ILQQTN------VTQEE----------------------H 820

2. Trichohyalin -**K**RRRHTLYA**K**PALQ-------EQLR**K**EQQ--LLQEEEEELQREERE**K**RRRQEQERQYRE 932

3. Cornulin -ET-VILRLDQGNLH-------TSV-----------------------------SSAQGQ 470

4. Repetin -QG-QSSHYGQP-DR-------QGQ-----------------------------NSHYGQ 453

5. Filaggrin 2 -FS-NSSSSNEFS----------------------------------------------- 414

6. Hornerin -FG-RSSSSGPYVSG-------SGY-----------------------------SSGFGH 467

7. Filaggrin -QSESSHGWTGPSTGVRQGSHHEQARDNSRHSASQDGQDTIRGHPGSS-RR---GRQGSH 856

1. Trichohyalin like protein 1 Q**K**QVQ-IAQASGPELCSVSLTSEISDCS-----------VFFNYSQ--ASQPYTRGLPLD 866

2. Trichohyalin EEQLQ----------------QEE-------------EQLLREERE**K**RRRQERERQYR**K**D 963

3. Cornulin DAAQS----------------------------------------------EE**K**RGI--- 481

4. Repetin ------------------------------------------------------------ 453

5. Filaggrin 2 ------------------------------------------------------------ 414

6. Hornerin HESSS----------------------------------------------EHSSGYTQH 481

7. Filaggrin HEQSVDRSGHSGSHHSHT-TSQGRSDASRGQSGSRSASRTTRNEE-QSRDGSRHSGSRHH 914

1. Trichohyalin like protein 1 ESP----------AGAQETPA---PQALED**K**QGH----PQRERLVLQREASTT**K**-Q---- 904

2. Trichohyalin **K**-----**K**LQQ**K**EEQLLGEEPE**K**RRRQERE**KK**YREEEELQQEEEQLLREERE**K**RRRQEWER 1018

3. Cornulin ------------------------------------------------------------ 481

4. Repetin ----------------------------TDR----QGQ---SSHYGQTDR-----QGQSS 473

5. Filaggrin 2 ---------------------------**K**CDQYGSGSSQ---STSFEQHGTGLSQSSGFEQ 444

6. Hornerin G-----SGSG-HSSGHGQHGSRSGQSSRGERQGSSags---ssSYGQHGSGSRQSLGHSR 532

7. Filaggrin EASSHADISRHSQAGQGQSEG--SRT--SRRQG--SSVSQDSDSE----GHSEDSERWSG 964

**Figure E3 continued**

1. Trichohyalin like protein 1 ------------------------------------------------------------ 904

2. Trichohyalin QYR**KK**DE---------------LQQEEEQ-L---LREERE**K**RRLQERERQYREEEELQQE 1059

3. Cornulin ----------------------TARELYS-Y---LRST**K**----P---------------- 495

4. Repetin HYSQPD**K**---QGQSSHYG**K**ID-RQDQSYH-Y---GQ-------P--------DG----QG 506

5. Filaggrin 2 HVCGSG----QT----CGQHESTSSQSLG-Y---DQHGSSSG**K**TS-GFGQHGSG----SG 487

6. Hornerin HGSGSG----QSPSPSRGRHESGSRQSSS-Y---GPHGYGSGRSS-SRGPYESG----SG 579

7. Filaggrin SASRNHRGSAQEQSRHGSRHPRSHHEDRAGHGHSADSSRQSGTPHae---tssGGQAASS 1021

1. Trichohyalin like protein 1 ------------------------------------------------------------ 904

2. Trichohyalin EEQ---LLGEERETR--------RRQEL-----------ERQYR**K**EEELQQEEEQL---- 1093

3. Cornulin ------------------------------------------------------------ 495

4. Repetin QSS---HYGQT------------DRQGQ-----------SFHYGQPDRQ-GQSSHY---- 535

5. Filaggrin 2 QSS---GFGQCG-----------SGSGQ-----------SSGFGQHGSVSGQSSGF---- 518

6. Hornerin HSS---GLGHQE-----------SRSGQ-----------SSGYGQHGSSSGHSSTH---- 610

7. Filaggrin HEQARSSPGERHGSRHQQSADSSRHSGIPRRQASSAVRdsghwgsSGSQASDSEGHSEES 1081

1. Trichohyalin like protein 1 ------------------------------------------------------------ 904

2. Trichohyalin ----L----REEPE**K**RRR-----------QERERQCREEEELQQEEEQL-LREERE**K**RRR 1133

3. Cornulin ------------------------------------------------------------ 495

4. Repetin ------------SQ-MDR-----------QGQSSHYGQTDR-Q----------------- 553

5. Filaggrin 2 ------------GQHGSV-----------SGQSSGFGQHESRS----------------- 538

6. Hornerin ------------GQHGST-----------SGQSSSCGQHGATS----------------- 630

7. Filaggrin DTQSVSGHGQDGPHQQSHQESARDWSGGRSGRSGSFIYQVSTHEQSESAHGRTRTSTGRR 1141

1. Trichohyalin like protein 1 ------------------------------------------------------------ 904

2. Trichohyalin QE----LERQYREEEEVQQEEEQLLREEPE**K**RRRQELERQYREEEELQQEEE-QLLREEQ 1188

3. Cornulin ------------------------------------------------------------ 495

4. Repetin -G----QSSHYGQTDR------------------QGQSYHYGQT---------------- 574

5. Filaggrin 2 -R-----QSSYGQHGS-----------------GSSQSSGYGQY---------------- 559

6. Hornerin -G----QSSSHGQHGS-----------------GSSQSSRYGQQ---------------- 652

7. Filaggrin QGSHHEQARDSS-RHSASQEGQDTIRAHPGSRRGGRQGSHHEQSVDRSGHSGSHHSHTTS 1200

**Figure E3 continued**

1. Trichohyalin like protein 1 ------------------------------------------------------------ 904

2. Trichohyalin E**K**RRQERERQYREEEELQRQ**K**R**K**Q-------RYRDEDQRSDL**K**WQWEPe**k**enaVRDN**K**VY 1241

3. Cornulin ------------------------------------------------------------ 495

4. Repetin -DR----------------QGQSS-------HYIQ--S-----------QTGEIQGQN**K**Y 597

5. Filaggrin 2 -GSRE--TSGFG-QHGL-GSGQST-------GFGQ--YGSGS------------------ 587

6. Hornerin -GSGSGQSPSRG-RHGS-DFGHSS-------SYGQ--HGSGSGWSSSNGPHGSVSGQSSG 700

7. Filaggrin QGRSDASHGQSGSR-SASRQTR**K**D**K**QSGDGSRHSGSRHHEAASWADS--SRHSQVGQEQS 1257

1. Trichohyalin like protein 1 ------------------------------------------------------------ 904

2. Trichohyalin C**K**GRENE----QFRQLED-----SQ-----LRDRQSQQD--------LQHLL-----GEQ 1274

3. Cornulin ------------------------------------------------------------ 495

4. Repetin FQGTEGTR**K**--------------ASYVEQSGRSGRLSQQTP------GQEGYQNQGQGFQ 637

5. Filaggrin 2 -------GQSSGFGQHGSGSGQSSGFGQHESRSGQS--------------SYGQHSSGS- 625

6. Hornerin FGH**K**SGSGQSSGYSQHGSGSSHSSGYR**K**HGSRSGQSSRSEQHGSSSGLSSSYGQHGSGS- 759

7. Filaggrin SGSRTSRHQGSSVSQDSDS-------------ERHSD-D--------SERLS-----GSA 1290

1. Trichohyalin like protein 1 ------------------------------------------------------------ 904

2. Trichohyalin QERDREQERRRWQQRD--RHFPEEEQLEREEQ**K**EA**K**RRDR**K**SQEE**K**QLLREEREE**K**RRRQ 1332

3. Cornulin ------------------------------------------------------------ 495

4. Repetin SRDSQQNGHQVWEPEEDSQHHQH------------------------------------- 660

5. Filaggrin 2 ---SQSSGYGQHGS---------------------------------------------- 636

6. Hornerin ---HQSSGHGRQGS--GSGHSPS------------------------------------- 777

7. Filaggrin SR--NHHGSSREQSRDGSRHPGF-HQEDRASHGHSADSSRQSGTH----HT--ESS---- 1337

1. Trichohyalin like protein 1 ------------------------------------------------------------ 904

2. Trichohyalin ETDR**K**FREEEQL-LQEREEQPLRRQERDR**K**FREEELRHQEQGR**K**FLEEEQRLRRQERER**K** 1391

3. Cornulin ------------------------------------------------------------ 495

4. Repetin ----**K**LLAQ----I--QQERPLCH**K**GRDWQSCS--------------------------- 683

5. Filaggrin 2 ----------------RQTSGFG-QHGSGSSQS--------------------------- 652

6. Hornerin ----RVRHG----SSSGHSSSHG-QHGSGTSCS--------------------------- 801

7. Filaggrin SHGQAVSSHEQARSSPGERHGSRHQQSADSSRHSGIGHRQA------------------- 1378

**Figure E3 continued**

1. Trichohyalin like protein 1 ------------------------------------------------------------ 904

2. Trichohyalin FL**K**EEQQLRCQEREQQLRQDRDR**K**FRE--------EEQQLSRQERDR**K**F------REEEQ 1437

3. Cornulin ------------------------------------------------------------ 495

4. Repetin -------SEQGHRQAQTRQSHG--EG---------------------------------- 700

5. Filaggrin 2 -------TGFGQYGSGSGQSSG--FG---------------------------------- 669

6. Hornerin -------SSCGHYESGSGQASG--FG---------------------------------- 818

7. Filaggrin --------SSAVRdsghrgsSGSQVTNSEGHSEDSDTQSVSAHGQAGPHQQSH**K**ESARGQ 1430

1. Trichohyalin like protein 1 ------------------------------------------------------------ 904

2. Trichohyalin QVRRQERER**K**FLEEEQQLRQE-----------------RHR**K**FREEEQLLQEREEQQLHR 1480

3. Cornulin ------------------------------------------------------------ 495

4. Repetin -------LSHWAEEEQGH-Q---------------------------------------- 712

5. Filaggrin 2 -------Q-----HVSGSGQ---------------------------------------- 677

6. Hornerin -------Q-----HESGSGQ---------------------------------------- 826

7. Filaggrin SGESSGRsrsflyqvsSHEQSESTHGQTAPSTGGRQGSRHEQARNSSRHSASQD------ 1484

1. Trichohyalin like protein 1 ------------------------------------------------------------ 904

2. Trichohyalin QERDR**K**FLEEEQQLRRQERDR**K**FREQELRSQEPER**K**FLEEEQQLHRQQRQR**K**FLQEEQQL 1540

3. Cornulin ------------------------------------------------------------ 495

4. Repetin -TWD--RHSHE------SQEGPCGTQDRRT------------------------------ 733

5. Filaggrin 2 -SSG--FGQHE------SRSG--------------------------------------- 689

6. Hornerin -G----YSQHG------SASGHFSSQGRHG------------------------------ 845

7. Filaggrin -GQD--TIRGHPGSSRGGRQGSYHEQSVDRSGHS-GYHH----SHTTPQGR--------- 1527

1. Trichohyalin like protein 1 ------------------------------------------------------------ 904

2. Trichohyalin RRQERGQQRRQDRDR**K**FREEEQLRQE---------------------------------- 1566

3. Cornulin ------------------------------------------------------------ 495

4. Repetin ---H**K**DEQNHQRRDRQTHEHEQSHQR---------------------------------- 756

5. Filaggrin 2 ------------------------------------------------------------ 689

6. Hornerin ---STSGQSS------------S------------------------------------- 853

7. Filaggrin SDASHGQSGPRSASRQTRNEEQSGDGSRHSGSRHHEPSTRAGSSRHSQVGQGESAGS**K**TS 1587

**Figure E3 continued**

1. Trichohyalin like protein 1 ------------------------------------------------------------ 904

2. Trichohyalin REEQQLSRQERDR**K**FRLEEQ**K**VRRQEQER**K**FMEDEQQ-LR----------RQEGQQQLRQ 1615

3. Cornulin ------------------------------------------------------------ 495

4. Repetin ----------RDRQTH--ED**K**QNRQRRDRQTHEDEQN-HQ----------R--------- 784

5. Filaggrin 2 -------------------------HSSYGQHGFGSS-QS----------SGYGQHGSSS 713

6. Hornerin ------------SGQH---DSSSGQSSSYGQHESASH-HA----------SGRGRHGSGS 887

7. Filaggrin RRQGSSVSQDRDSEGHSEDSERRSESASRNHYGSAREQSRHGSRNPRSHQEDRASHGHSA 1647

1. Trichohyalin like protein 1 ------------------------------------------------------------ 904

2. Trichohyalin ER--DR**K**FREDEQLLQEREEQQLHRQERDR**K**FLEEEPQLRRQEREQQLRHD--RDR--**K**F 1669

3. Cornulin ------------------------------------------------------------ 495

4. Repetin ------------------------------------------------------------ 784

5. Filaggrin 2 GQ--TSGFGQHEL------------------SSGQSSSFGQHGSG----SG--QSS--GF 745

6. Hornerin GQ--SPGHGQRGS------------------GSGQSPSYGRHGSG----SG--RSS--SS 919

7. Filaggrin ESSRQSGTRHaetss---GGQAASSQEQAR----SSPGE-RHGSRHQQSadsstDSGTGR 1699

1. Trichohyalin like protein 1 ------------------------------------------------------------ 904

2. Trichohyalin REEEQLLQEGEEQ-QLRRQERD-R**K**FREE------------------------EQQLRRQ 1703

3. Cornulin ------------------------------------------------------------ 495

4. Repetin ------------------------------------------------------------ 784

5. Filaggrin 2 GQH----GSGSG--------QS-SGFGQH------------------------ESR---- 764

6. Hornerin GRH----GSGSG--------QS-SGFGH**K**------------------------SSS---- 938

7. Filaggrin RQDSSVVGdsgnrgsSGSQASDSEGHSEESDTQSVSAHGQAGPHQQSHQESTRGQSGERS 1759

1. Trichohyalin like protein 1 ------------------------------------------------------------ 904

2. Trichohyalin ERER**K**FLQEEQQLRRQEL-------------------ER**K**F-------REEEQLRQETEQ 1737

3. Cornulin ------------------------------------------------------------ 495

4. Repetin ------------------------------------------------------------ 784

5. Filaggrin 2 SGQSSYGQHSSG------------------------------------------------ 776

6. Hornerin GQSSGYTQHGSG------------------------------------------------ 950

7. Filaggrin GRSGSFLYQVSTHEQSESAHGRTGPSTGGRQRSRHEQARDSSRHSASQEGQDTIRGHPGS 1819

**Figure E3 continued**

1. Trichohyalin like protein 1 ------------------------------------------------------------ 904

2. Trichohyalin -EQLRRQERYR**K**ILEEEQLRPEREEQQLRRQERD-R**K**FREEE--Q-LRQEREEQQLRSQE 1792

3. Cornulin ------------------------------------------------------------ 495

4. Repetin ------------------------------------------------------------ 784

5. Filaggrin 2 ---SSQSSGYGQHGSR-QT----------------SGFGQHG--SGSSQSTGFGQYG--- 811

6. Hornerin ---SGHSSSYEQHGSR-SGQSSRSEQHGSSSGSS-SSYGQHG--SGSRQSLGHGQHGSGS 1003

7. Filaggrin SRGGRQGSHYEQSVDSSGH-SGSHHSHTTSQERSDVSRGQSGSRSVSRQTRNE**K**QSGDGS 1878

1. Trichohyalin like protein 1 ------------------------------------------------------------ 904

2. Trichohyalin SDR**K**FREEEQ--LRQEREEQQLRPQQ-------------------RDG------------ 1819

3. Cornulin ------------------------------------------------------------ 495

4. Repetin ------------------------------------------------------------ 784

5. Filaggrin 2 ------------------------------------------------------------ 811

6. Hornerin GQ-----SPS--PSRGRHGSGSGQSS-------------------SYG------------ 1025

7. Filaggrin RHSGSRHHEASSRADSSRHSQVGQGQSSGPRTSRNQGSSVSQDSDSQGHSEDSERWSGSA 1938

1. Trichohyalin like protein 1 ------------------------------------------------------------ 904

2. Trichohyalin -**K**Y----RWEEEQLQLE-------------EQEQRLRQERDRQYRAEEQFATQE**K**SRREE 1861

3. Cornulin ------------------------------------------------------------ 495

4. Repetin ------------------------------------------------------------ 784

5. Filaggrin 2 ----SGSGQSAGFGQHG-SGSGQS--SGFGQHESRSHQ---SSYGQHGSGSSQ------- 854

6. Hornerin -PYRSGSGWSSSRGPYE-SGSGHS--SGLGHRESRSGQ--SSGYGQHGSSSGH------- 1072

7. Filaggrin SRNHLGSAWEQSRDGSRHPGSHHEDRAGHGHSADSSRQ---------------SGTRHTE 1983

1. Trichohyalin like protein 1 ------------------------------------------------------------ 904

2. Trichohyalin QELWQEEEQ**K**RRQERER**K**LREEHI---RRQQ**K**EEQRHRQVGEI------**K**SQEG**K**GHGRL 1912

3. Cornulin ------------------------------------------------------------ 495

4. Repetin ------------------------------------------------------------ 784

5. Filaggrin 2 SSGYGQHGSSSGQT--SGFG-QHR----SSSGQYSGFGQHGSGSGQSSGFGQHGTGSGQ- 906

6. Hornerin SSTHGQHGSTSGQS--SSCG-QHG----ASSGQSSSHGQHGSGSSQSSGYGRQGSGSGQ- 1124

7. Filaggrin SSSRGQAASSHEQAR-SSAGERHGSHHQLQSADSSRH---------------SGIGHGQ- 2026

**Figure E3 continued**

1. Trichohyalin like protein 1 ------------------------------------------------------------ 904

2. Trichohyalin LEPGTHQFASVPVRSSPLYEYIQ------------------------------------- 1935

3. Cornulin ------------------------------------------------------------ 495

4. Repetin ------------------------------------------------------------ 784

5. Filaggrin 2 -YSGFGQHES-RS-HQSSYGQHGSGSSQS---------------------------SGYG 936

6. Hornerin -SPGHGQRGS-GSRQSPSYGRHGSGSGRS---------------------------SSSG 1155

7. Filaggrin --------ASSAVRDSGHRGYSGSQASDSEGHSEDSDTQSVSAQG**K**AGPHQQSH**K**ESARG 2078

1. Trichohyalin like protein 1 ------------------------------------------------------------ 904

2. Trichohyalin ------------------------------------------------------------ 1935

3. Cornulin ------------------------------------------------------------ 495

4. Repetin ------------------------------------------------------------ 784

5. Filaggrin 2 QHGSSSGQTFGFGQHRSGSGQSSGFGQHGSGSGQSSGFGQHESGSG**K**S--SGFGQH-ESR 993

6. Hornerin QHGSGLGESSGFGHHESSSGQSSSYSQHGSGSGHSSGYGQHGSRSGQS--SRGERHGSSS 1213

7. Filaggrin QSGESSGRSGSFLYQVSTHEQ-------------------SESTHGQSAPSTGGRQ---- 2115

1. Trichohyalin like protein 1 ------------------------------------------------------------ 904

2. Trichohyalin ------------------------------------------------------------ 1935

3. Cornulin ------------------------------------------------------------ 495

4. Repetin ------------------------------------------------------------ 784

5. Filaggrin 2 SSQSNYGQHGSGSSQSSGYGQHGSSSGQ-------------------------------- 1021

6. Hornerin GSSSHYGQHGSGSRQSSGHGRQGSGSGHSPSRGRHGSGLGHSSSHGQHGSGSGRSSSRGP 1273

7. Filaggrin --GSHYDQAQDSSRHSAS------QEGQDTIRGHPGPSRGG-----RQGS------HQEQ 2156

1. Trichohyalin like protein 1 ------------------------------------------------------------ 904

2. Trichohyalin ---------------------------------------------------------EQR 1938

3. Cornulin ------------------------------------------------------------ 495

4. Repetin ------------------------------------------------------------ 784

5. Filaggrin 2 -------TTGFGQHRSSSGQYSGFGQHGSGSDQSSGFGQHGTGSGQSSGFGQYES-RSRQ 1073

6. Hornerin YESRSGHSSVFGQHESGSGHSSAYSQHGSGSGHFCSQGQHGSTSGQSSTFDQEGSSTGQS 1333

7. Filaggrin SVDRSGHSGSHHSHTTSQGR------------SDASRGQSGSRSASR**K**TYD**K**EQ------ 2198

**Figure E3 continued**

1. Trichohyalin like protein 1 ------------------------------------------------------------ 904

2. Trichohyalin SQYRP------------------------------------------------------- 1943

3. Cornulin ------------------------------------------------------------ 495

4. Repetin ------------------------------------------------------------ 784

5. Filaggrin 2 SSYGQHGSGSSQSSGYGQHGSNSGQTSGFGQHRPGSGQSSGFGQYGSGSGQSSGFGQHGS 1133

6. Hornerin SSYGHRGSGSSQSSGYGRHGAGSGQSPSRGRHGSGSGHSSSYGQHGSGSGWSSSSGRHGS 1393

7. Filaggrin ---------------------------------SGDGSRHSGSHHHEASSWADSSRHSLV 2225

1. Trichohyalin like protein 1 ------------------------------------------------------------ 904

2. Trichohyalin ------------------------------------------------------------ 1943

3. Cornulin ------------------------------------------------------------ 495

4. Repetin ------------------------------------------------------------ 784

5. Filaggrin 2 GTG**K**SSGFAQHEY--------------------RSGQSSYGQHGTGSSQSSGCG-----Q 1168

6. Hornerin GSGQSSGFGHHES--------------------------------SSWQSSGCT-----Q 1416

7. Filaggrin GQGQSSGPRTSRPRGSSVSQDSDSEGHSEDSERRSGSASRNHHGSAQEQSRDGSRHPRSH 2285

1. Trichohyalin like protein 1 ------------------------------------------------------------ 904

2. Trichohyalin ------------------------------------------------------------ 1943

3. Cornulin ------------------------------------------------------------ 495

4. Repetin ------------------------------------------------------------ 784

5. Filaggrin 2 H--ESGSGPTTSF-GQHVSG---------------SDNFSSSGQ---------------- 1194

6. Hornerin H--GSGSGHSSSY-EQHGSR---------------SGQSSRGERHGSSSGSSSSYGQHGS 1458

7. Filaggrin HEDRAGHGHSAESSRQSGTHHAENSSGGQAASSHEQARSSAGERHGSHHQQSAD-----S 2340

1. Trichohyalin like protein 1 ------------------------------------------------------------ 904

2. Trichohyalin ------------------------------------------------------------ 1943

3. Cornulin ------------------------------------------------------------ 495

4. Repetin ------------------------------------------------------------ 784

5. Filaggrin 2 ------------------------------------------------------------ 1194

6. Hornerin GSRQSLGHGQHG--------------------------------------SGSGQSPSPS 1480

7. Filaggrin SRHSGIGHGQASSAVRdsghrgsSGSQASDSEGHSEDSDTQSVSAHGQAGPHQQSHQEST 2400

**Figure E3 continued**

1. Trichohyalin like protein 1 ------------------------------------------------------------ 904

2. Trichohyalin ------------------------------------------------------------ 1943

3. Cornulin ------------------------------------------------------------ 495

4. Repetin ------------------------------------------------------------ 784

5. Filaggrin 2 ---HISdsgqstGFGQYGSGSGQSTGLGQGESQQVESGSTVHGRQETTHGQTINTTRHSQ 1251

6. Hornerin RGRHGSGSGQSSSYSPYGSGSGWSSSRGPYESGSSHSS--GLGHRESRSGQSSGYGQHGS 1538

7. Filaggrin RGRSAGRSGRSGSFLYQVST------HEQSESAHGRTGTSTGGRQGSHH**K**QARDSSRHST 2454

1. Trichohyalin like protein 1 ------------------------------------------------------------ 904

2. Trichohyalin ------------------------------------------------------------ 1943

3. Cornulin ------------------------------------------------------------ 495

4. Repetin ------------------------------------------------------------ 784

5. Filaggrin 2 SGQGQSTQTGSRVTRRRRSSQSENSDSEVHS**K**VSHRHSEHIH-----TQAGSHYP**K**SGST 1306

6. Hornerin S-SGHSSTHGQHGS-----TSGQSSSCGQHGASSGQSSSHGQH-GSGSSQSSGYGRQGSG 1591

7. Filaggrin SQEGQDTIHGHpgsssGGR---QGSHYEQLVDRSGHSGSHHSHTTSQGRSDASHGHSGSR 2511

1. Trichohyalin like protein 1 ------------------------------------------------------------ 904

2. Trichohyalin ------------------------------------------------------------ 1943

3. Cornulin ------------------------------------------------------------ 495

4. Repetin ------------------------------------------------------------ 784

5. Filaggrin 2 VRRRQGTTHGQRGDTTRHGH------SGHGQSTQTGSR---TSGRQRFSHSDATDSEVH- 1356

6. Hornerin S--GQSPGHGQRGSGSRQSPSYGRHGSGSGRSSSSGQHGSGLGESSGFGHHESSSGQSSS 1649

7. Filaggrin SASRQTRNDEQSGDGSRHSGSRHHEA--SSRADSSGHSQVGQGQSEGPRT---SRNWGSS 2566

1. Trichohyalin like protein 1 ------------------------------------------------------------ 904

2. Trichohyalin ------------------------------------------------------------ 1943

3. Cornulin ------------------------------------------------------------ 495

4. Repetin ------------------------------------------------------------ 784

5. Filaggrin 2 -----SGVSHRP-------------HSQEQTHSQAGSQHGESESTVHERHETTYGQTGEA 1398

6. Hornerin YSQHGSGSGHSS---------GYGQHGSRSGQSSRGERHGS-----SSRSSSRYGQHGSG 1695

7. Filaggrin FSQDSDSQGHSEDSERWSGSASRNHHGSAQEQLRDGSRHPRSH----------------- 2609

**Figure E3 continued**

1. Trichohyalin like protein 1 ------------------------------------------------------------ 904

2. Trichohyalin ------------------------------------------------------------ 1943

3. Cornulin ------------------------------------------------------------ 495

4. Repetin ------------------------------------------------------------ 784

5. Filaggrin 2 ----TGHGHSG--HGQSTQRGS-----------------RTTGRRGSGHSESSDSEVHSG 1435

6. Hornerin SRQSSGHGRQGSGSGQSPSR----------------------GRHGSGLGHSSSHGQHGS 1733

7. Filaggrin QEDRAGHGHSADSSRQSGTRHTQTSSGGQAASSHEQARSSAGERHGSHHQQSADSSRHSG 2669

1. Trichohyalin like protein 1 ------------------------------------------------------------ 904

2. Trichohyalin ------------------------------------------------------------ 1943

3. Cornulin ------------------------------------------------------------ 495

4. Repetin ------------------------------------------------------------ 784

5. Filaggrin 2 -GSHRPQSQEQ---------THGQ---------------AGSQHGES-------GSTVHG 1463

6. Hornerin -GSGRSSSRGPYESRSGHSSVFGQHE-SGSGHS-----SAYSQHGSGS-----GHFCSQG 1781

7. Filaggrin IGHGQASSAV---RDSGHRGYSGSQASDNEGHSEDSDTQSVSAHGQAGSHQQSHQESARG 2726

1. Trichohyalin like protein 1 ------------------------------------------------------------ 904

2. Trichohyalin ------------------------------------------------------------ 1943

3. Cornulin ------------------------------------------------------------ 495

4. Repetin ------------------------------------------------------------ 784

5. Filaggrin 2 RHGTTHGQTGDTTR----------H----------AHYHHG**K**STQRGSSTTGRRGSGHSE 1503

6. Hornerin QHGSTSGQSSTFDQEGSSTGQSSSHGQHGSGSSQSSSYGQQGSGSGQSPSRGRHGSGSGH 1841

7. Filaggrin RSGETSGHSGSFLYQVS------------------------------------------- 2743

1. Trichohyalin like protein 1 ------------------------------------------------------------ 904

2. Trichohyalin ------------------------------------------------------------ 1943

3. Cornulin ------------------------------------------------------------ 495

4. Repetin ------------------------------------------------------------ 784

5. Filaggrin 2 SSDSEVHSGGSHTHSGHTHGQSGSQHGESESIIH--------------------DRHRIT 1543

6. Hornerin SSSYGQHGSGSGW--SSSSGRHGSGSGQSSGFGHH--------------ESSSWQSSGYT 1885

7. Filaggrin --THEQSESSHGWTGPSTRGRQGSRHEQAQDSSRHSASQDGQDTIRGHPGSSRGGRQGYH 2801

**Figure E3 continued**

1. Trichohyalin like protein 1 ------------------------------------------------------------ 904

2. Trichohyalin ------------------------------------------------------------ 1943

3. Cornulin ------------------------------------------------------------ 495

4. Repetin ------------------------------------------------------------ 784

5. Filaggrin 2 H------------------------------------------GQTGDTTRHSYSGHEQ- 1560

6. Hornerin Q------------------------------------------HGSGSGHSSSYEQHGS- 1902

7. Filaggrin HEHSVDSSGHSGSHHSHTTSQGRSDASRGQSGSRSASRTTRNEEQSGDGSRHSGSRHHEA 2861

1. Trichohyalin like protein 1 ------------------------------------------------------------ 904

2. Trichohyalin ------------------------------------------------------------ 1943

3. Cornulin ------------------------------------------------------------ 495

4. Repetin ------------------------------------------------------------ 784

5. Filaggrin 2 -----TTQTGSRTTG---------RQRTSHSESTDSEVHSGGSHRPHSREHTYGQAGSQH 1606

6. Hornerin -------RSGQSSRG---------EQHGSS----------------SGSSSSYGQHGSGS 1930

7. Filaggrin STHADISRHSQAVQGQSEGSRRSRRQGSSVSQDSDSEGHSEDSERWSGSA---------- 2911

1. Trichohyalin like protein 1 ------------------------------------------------------------ 904

2. Trichohyalin ------------------------------------------------------------ 1943

3. Cornulin ------------------------------------------------------------ 495

4. Repetin ------------------------------------------------------------ 784

5. Filaggrin 2 EEPEFTVHERHGTTHGQIGDTTGHSHSGHGQSTQRGSRTTGRQRSSHSESSDSEVHSGVS 1666

6. Hornerin R----------------------------------------------------------- 1931

7. Filaggrin ------SRNHHGSAQEQLRDGSRHP------------RSHQEDRAGHGHSADSSRQSGTR 2953

1. Trichohyalin like protein 1 ------------------------------------------------------------ 904

2. Trichohyalin ------------------------------------------------------------ 1943

3. Cornulin ------------------------------------------------------------ 495

4. Repetin ------------------------------------------------------------ 784

5. Filaggrin 2 HTHTGHTHGQAGSQHGQSESIVPERHGTTHGQTGDTTRHAHYHHG--LTTQ--------- 1715

6. Hornerin ---QSLGHGQHGSGSGQSPSPSRGRHGSGSGQSS---SYGPYGSGSGWSSS--------- 1976

7. Filaggrin HTQT-SSGGQAASSHEQARSSAGERHGSHHQQSADSSRHSGIGHGQASSAVRDSGHRGYS 3012

**Figure E3 continued**

1. Trichohyalin like protein 1 ------------------------------------------------------------ 904

2. Trichohyalin ------------------------------------------------------------ 1943

3. Cornulin ------------------------------------------------------------ 495

4. Repetin ------------------------------------------------------------ 784

5. Filaggrin 2 ------------------------------------------------------------ 1715

6. Hornerin ------------------------------------------------------------ 1976

7. Filaggrin GSQASDNEGHSEDSDTQSVSAHGQAGSHQQSHQESARGRSGETSGHSGSFLYQVSTHEQS 3072

1. Trichohyalin like protein 1 ------------------------------------------------------------ 904

2. Trichohyalin ------------------------------------------------------------ 1943

3. Cornulin ------------------------------------------------------------ 495

4. Repetin ------------------------------------------------------------ 784

5. Filaggrin 2 ------TGSRTTGRRGSGHSEYSDSEGYSG--------VSHTH--SGHTHGQARSQHGE- 1758

6. Hornerin ------RGPY---ESGSGHS--------SG--------LGHRESRSGQSSGY--GQHGSS 2009

7. Filaggrin ESSHGWTGPSTRGRQGSRHEQAQDSSRHSASQYGQDTIRGHPGSSRGGRQGYHHEHSVDS 3132

1. Trichohyalin like protein 1 ------------------------------------------------------------ 904

2. Trichohyalin ------------------------------------------------------------ 1943

3. Cornulin ------------------------------------------------------------ 495

4. Repetin ------------------------------------------------------------ 784

5. Filaggrin 2 -SESIVHERHGTIHGQTGD-T----------TRHAHS-----------------GHGQST 1789

6. Hornerin SGHSSTHGQHGSASGQSSS-CGQHGASSGQSSSHGQHGSGSSQSSGYGR--QGSGSGQSP 2066

7. Filaggrin SGHSGSHHSHTTSQGRSDASRGQSGSRSASRTTRNEEQSGDSSRHSVSRHHEASTHADIS 3192

1. Trichohyalin like protein 1 ------------------------------------------------------------ 904

2. Trichohyalin ------------------------------------------------------------ 1943

3. Cornulin ------------------------------------------------------------ 495

4. Repetin ------------------------------------------------------------ 784

5. Filaggrin 2 QTGS----RTTGRRS--------SGHSEYSD-------SEGHSG--------F------- 1815

6. Hornerin GHGQ----RGSGSRQSPSYGRHGSGSGRSSSSGQHGPGLGESSG--------F------- 2107

7. Filaggrin RHSQAVQGQSEGSRRSR---RQGSSVSQDSDSEGHSEDSERWSGSASRNHRGSVQEQSRH 3249

**Figure E3 continued**

1. Trichohyalin like protein 1 ------------------------------------------------------------ 904

2. Trichohyalin ------------------------------------------------------------ 1943

3. Cornulin ------------------------------------------------------------ 495

4. Repetin ------------------------------------------------------------ 784

5. Filaggrin 2 ------S----------------QRPHSRGHTHG--QAGSQHGESESIVDERHGTTH--- 1848

6. Hornerin ------GHHESSSGQSSSY---SQHGSGSGHSSGYGQHGSRSG--QSSRGERHGSSSGSS 2156

7. Filaggrin GSRHPRSHHEDRAGHGHSADRSRQSGTRHaetssGGQAASSHEQARSSPGERHGSRHQQS 3309

1. Trichohyalin like protein 1 ------------------------------------------------------------ 904

2. Trichohyalin ------------------------------------------------------------ 1943

3. Cornulin ------------------------------------------------------------ 495

4. Repetin ------------------------------------------------------------ 784

5. Filaggrin 2 ------------------------GQTG----DTSGHSQS-------GHGQSTQSGSSTT 1873

6. Hornerin SRYGQHG--------SGSRQSSGHGRQG----SGSGHSPSR-GRHGSGSGHSSSHGQHGS 2203

7. Filaggrin ADSSRHSGIPRGQASSAVRDSRHWGSSGSQASDSEGHSEESDTQSVSGHGQAGPHQQSHQ 3369

1. Trichohyalin like protein 1 ------------------------------------------------------------ 904

2. Trichohyalin ------------------------------------------------------------ 1943

3. Cornulin ------------------------------------------------------------ 495

4. Repetin ------------------------------------------------------------ 784

5. Filaggrin 2 G----------RRRSGHSESSDSEVHSGGSHTHSGHTHSQARSQHGESESTVH--**K**RHQT 1921

6. Hornerin GSGRSSSRGPYESRSGHSSVFG--------QHESGSGHSSAYSQHGSGSGHFCSQGQHGS 2255

7. Filaggrin ES----ARDRSGGRSGRSGSFLYQVST---HEQSESAHGRTRTSTGRRQGSHHEQARDSS 3422

1. Trichohyalin like protein 1 ------------------------------------------------------------ 904

2. Trichohyalin ------------------------------------------------------------ 1943

3. Cornulin ------------------------------------------------------------ 495

4. Repetin ------------------------------------------------------------ 784

5. Filaggrin 2 THGQ----------TGDTTEHGHPSHGQTIQ--TG--------SRT-TGRRGS--GHSE- 1957

6. Hornerin TSGQSSTFDQEGSSTGQSSSHGQHGSGSSQSSSYGQQGSGSGQSPS-RGRHGSGSGHSSS 2314

7. Filaggrin --------RHSASQEGQDTIRGHPGSSR-----RGRQGSHYEQSVDRSGHSGSHHSHTTS 3469

**Figure E3 continued**

1. Trichohyalin like protein 1 ------------------------------------------------------------ 904

2. Trichohyalin ------------------------------------------------------------ 1943

3. Cornulin ------------------------------------------------------------ 495

4. Repetin ------------------------------------------------------------ 784

5. Filaggrin 2 Y---------------------------------SDSEG---------PSGVSHT-HSGH 1974

6. Hornerin YG------------------------QHGSGSGWSSSSG---------RHGSGSGQSSGF 2341

7. Filaggrin QGRSDASRGQSGSRSASRQTRNDEQSGDGSRHSWSHHHEASTQADSSRHSQSGQGQSAGP 3529

1. Trichohyalin like protein 1 ------------------------------------------------------------ 904

2. Trichohyalin ------------------------------------------------------------ 1943

3. Cornulin ------------------------------------------------------------ 495

4. Repetin ------------------------------------------------------------ 784

5. Filaggrin 2 -----------------TH-GQ----AGSHYPESG-----SSVHERHGTTHGQTADTTRH 2007

6. Hornerin -----------------GH-HESSSWQSSGYTQHGSGSGHSSSYEQHGSRSGQSSRGERH 2383

7. Filaggrin RTSRNQGSSVSQDSDSQGHSEDSERWSGSASRNH------------RGSAQEQSRDGSRH 3577

1. Trichohyalin like protein 1 ------------------------------------------------------------ 904

2. Trichohyalin ------------------------------------------------------------ 1943

3. Cornulin ------------------------------------------------------------ 495

4. Repetin ------------------------------------------------------------ 784

5. Filaggrin 2 GHSGHGQSTQRGSRTTGRRASGHSEYSDSEGHSGVSHTHSGHAHGQAGSQHGESGSSVHE 2067

6. Hornerin GSSSGS----------------SSSYGQ----HGSG-SRQSLGHGQHGSGSGQSPSPSRG 2422

7. Filaggrin PTSHHEDRAGH----------GHS--AESSRQSGTH-HAENSSGGQAASSHEQARSSAGE 3624

1. Trichohyalin like protein 1 ------------------------------------------------------------ 904

2. Trichohyalin ------------------------------------------------------------ 1943

3. Cornulin ------------------------------------------------------------ 495

4. Repetin ------------------------------------------------------------ 784

5. Filaggrin 2 RHGTTHGQTGDTTRHAHSGHGQSTQRGSRTAGRRGSGHSESSDSEVHSGVSHTH--S--- 2122

6. Hornerin RHGSGSGQSSSYSP-YGSGSGWSSSRGPYE---SGSGHS--------SGLGHRESRS--- 2467

7. Filaggrin RHGSHHQQSADSSRHSGIGHGQASSAV-RdsghrgsSGSQASDSEGHSEDSDTQSVSAHG 3683

**Figure E3 continued**

1. Trichohyalin like protein 1 ------------------------------------------------------------ 904

2. Trichohyalin ------------------------------------------------------------ 1943

3. Cornulin ------------------------------------------------------------ 495

4. Repetin ------------------------------------------------------------ 784

5. Filaggrin 2 --------G--HTYG--------------------QARSQHGESGSAIHGRQGTIHGQTG 2152

6. Hornerin --------GQSSGYG--------------------QHGSSSG--HSSTHGQHGSTSGQSS 2497

7. Filaggrin QAGPHQQSHQESTRGRSAGRSGRSGSFLYQVSTHEQSESAHGRAGPSTGGRQGSRHEQAR 3743

1. Trichohyalin like protein 1 ------------------------------------------------------------ 904

2. Trichohyalin ------------------------------------------------------------ 1943

3. Cornulin ------------------------------------------------------------ 495

4. Repetin ------------------------------------------------------------ 784

5. Filaggrin 2 ----------DTTRHGQSGHGQSTQ---------------TGSRTTGRQRS--------S 2179

6. Hornerin SCGQHGASSGQSSSHGQHGSGSSQSSGYGRQGSGSGQSPGHGQRGSGSRQSPSYGRHGSG 2557

7. Filaggrin D----------SSRHSASQEGQDTIRG--H----------PGSRRG--------GRQGSY 3773

1. Trichohyalin like protein 1 ------------------------------------------------------------ 904

2. Trichohyalin ------------------------------------------------------------ 1943

3. Cornulin ------------------------------------------------------------ 495

4. Repetin ------------------------------------------------------------ 784

5. Filaggrin 2 HSESSDSEVHSEASPTHSG--HTHSQAG-----SRH--GQSGSSGHGRQGTTHGQTGDTT 2230

6. Hornerin SGRSSSSGQHGSGLGESSGFGHHESSSGQSSSYSQHGSGSGHSSGYGQHGSRSGQSSRGE 2617

7. Filaggrin HEQSVDR-------SGHSGSHHSHTTSQ-GRSDASH--GQSGSRSASRETRNEEQSGDGS 3823

1. Trichohyalin like protein 1 ------------------------------------------------------------ 904

2. Trichohyalin ------------------------------------------------------------ 1943

3. Cornulin ------------------------------------------------------------ 495

4. Repetin ------------------------------------------------------------ 784

5. Filaggrin 2 -----------------RHAHYGYGQSTQRGSRTTGRRGSGHSESSDSEVHSWGSH---T 2270

6. Hornerin RHGSS----------SGSSSHYGQHGSGSRQSSGHGRQGSGSGQSPSRGRHGSGLG---H 2664

7. Filaggrin RHSGSRHHEASTQADSSRHSQSGQG--ESAGSRRSRRQGSSVSQDSDSEAYPEDSERRSE 3881

**Figure E3 continued**

1. Trichohyalin like protein 1 ------------------------------------------------------------ 904

2. Trichohyalin ------------------------------------------------------------ 1943

3. Cornulin ------------------------------------------------------------ 495

4. Repetin ------------------------------------------------------------ 784

5. Filaggrin 2 HSG---HIQG--QAGSQQRQP------GSTVHGRLETTHGQTGDTTRHGH--SGYGQSTQ 2317

6. Hornerin SSSHGQHGSG--SGRSSSRGPYESRLGHSSVFGQHESGSGHSSAYSQHGSGSGHFCSQGQ 2722

7. Filaggrin SASRNHHGSSREQSRDGSRHPGSSHRDTA-SHVQSSPVQSDSSTA**K**EH----GHFSSLSQ 3936

1. Trichohyalin like protein 1 ------------------------------------------------------------ 904

2. Trichohyalin ------------------------------------------------------------ 1943

3. Cornulin ------------------------------------------------------------ 495

4. Repetin ------------------------------------------------------------ 784

5. Filaggrin 2 TGSRSSRASHFQSHSS---------------------ERQRHGSSQVW**K**HGS-------- 2348

6. Hornerin HGSTSGQSSTFDQEGSSTGQSSSYGHRGSGSSQSSGYGRHGAGSGQSLSHGRHGSGSGQS 2782

7. Filaggrin DSA---YHSGIQSRGSp-hsssSY------HYQSEGTERQ**K**GQSGLVWRHGS-------- 3978

1. Trichohyalin like protein 1 ------------------------------------------------------------ 904

2. Trichohyalin ------------------------------------------------------------ 1943

3. Cornulin ------------------------------------------------------------ 495

4. Repetin ------------------------------------------------------------ 784

5. Filaggrin 2 ----------------------YGPAEYDYGHTGYGPSGGSR**K**SISNSHLSWSTDS---- 2382

6. Hornerin SSYGQHGSGSGQSSGYSQHGSGSGQDGYSYC**K**GGSNHDGGSSGS---YFLSFpsst---- 2835

7. Filaggrin ----------------------YGSADYDYGESGFRHSQHGSVSYNSNPVVF**K**ERSDIC**K** 4016

1. Trichohyalin like protein 1 --------------------------------------------- 904

2. Trichohyalin --------------------------------------------- 1943

3. Cornulin --------------------------------------------- 495

4. Repetin --------------------------------------------- 784

5. Filaggrin 2 ------------------------------TAN**K**QLSRH------ 2391

6. Hornerin ------------------------------sPYEYVQEQRCYFYQ 2850

7. Filaggrin ASAFG**K**DHPRYYATYIN**K**DPGLCGHSSDIS**K**QLGFSQSQRYYYYE 4061

**Figure E3 continued**

A. S100A1 ------------------------M---GSELETAMETLINVFHAHSG**K**EG-D**K**Y**K**LS**KK** 32

B. S100A2 isoform 1 -----------------------MM---CSSLEQALAVLVTTFH**K**YSCQEG-D**K**F**K**LS**K**G 33

C. S100A2 isoform 2 -----------------------MM---CSSLEQALAVLVTTFH**K**YSCQEG-D**K**F**K**LS**K**G 33

D. S100A2 isoform 3 ------------------------------------------------------------ 0

E. S100A3 ------------------------M---ARPLEQAVAAIVCTFQEYAGRCG-D**K**Y**K**LCQA 32

F. S100A4 ------------------------M---ACPLE**K**ALDVMVSTFH**K**YSG**K**EG-D**K**F**K**LN**K**S 32

G. S100A5 ------------------------M---ETPLE**K**ALTTMVTTFH**K**YSGREG-S**K**LTLSR**K** 32

H. S100A6 ------------------------M---ACPLDQAIGLLVAIFH**K**YSGREG-D**K**HTLS**KK** 32

I. S100A7 -----------------------MS---NTQAERSIIGMIDMFH**K**YTRRDD----**K**IE**K**P 30

J. S100A7A -----------------------MS---NTQAERSIIGMIDMFH**K**YTGRDG----**K**IE**K**P 30

K. S100A8 MSLVSCLSEDL**K**VLFFRWG**K**SVGIM---LTELE**K**ALNSIIDVYH**K**YSLI**K**G-NFHAVYRD 56

L. S100A9 ---------------------MTC**K**---MSQLERNIETIINTFHQYSV**K**LG-HPDTLNQG 35

M. S100A10 ------------------------M---PSQMEHAMETMMFTFH**K**FAGD**K**G----YLT**K**E 29

N. S100A11 -------------------MA**K**ISS---PTETERCIESLIAVFQ**K**YAG**K**DG-YNYTLS**K**T 37

O. S100A12 ----------------------------MT**K**LEEHLEGIVNIFHQYSVR**K**G-HFDTLS**K**G 31

P. S100A13 -----------------------MAAEPLTELEESIETVVTTFFTFARQEG-R**K**DSLSVN 36

Q. S100A14 --------------MGQCRSANAEDAQEFSDVERAIETLI**K**NFHQYSV-EG-G**K**ETLTPS 44

R. S100A16 ---------------------MSDC---YTELE**K**AVIVLVENFY**K**YVS**K**YSLV**K**N**K**IS**K**S 36

I. S100B ----------------------------MSELE**K**AMVALIDVFHQYSGREG-D**K**H**K**L**KK**S 31

II. S100G -----------------------------MST**KK**SPEEL**K**RIFE**K**YAA**K**EG-DPDQLS**K**D 30

III. S100P ----------------------------MTELETAMGMIIDVFSRYSGSEG-STQTLT**K**G 31

IV. S100Z ------------------------M---PTQLEMAMDTMIRIFHRYSG**K**ER-**K**RF**K**LS**K**G 32

1. Trichohyalin like protein 1 ----------------------------MPQLLRNVLCVIETFH**K**YASEDS-NGATLTGR 31

2. Trichohyalin ----------------------------MSPLLRSICDITEIFNQYVSHDC-DGAALT**KK** 31

3. Cornulin ----------------------------MPQLLQNINGIIEAFRRYARTEG-NCTALTRG 31

4. Repetin ----------------------------MAQLLNSILSVIDVFH**K**YA**K**GNG-DCALLC**K**E 31

5. Filaggrin 2 ----------------------------MTDLLRSVVTVIDVFY**K**YT**K**QDG-ECGTLS**K**G 31

6. Hornerin ----------------------------MP**K**LLQGVITVIDVFYQYATQHG-EYDTLN**K**A 31

7. Filaggrin ----------------------------MSTLLENIFAIINLF**K**QYS**KK**D**K**-NTDTLS**KK** 31

**Figure E4: Amino acid sequence alignment of S100 proteins and N terminal (aa 1 to 100) of S100 fused-type proteins.** Lysine (**K**), serine (S), threonine (T) and cysteine (C) residues are considered as conserved if shared by at least two S100 domain (marked as green).

A. S100A1 EL**K**ELLQTELSGFLDAQ--**K**DVDAVD---**K**VM**K**ELDENGDGEVDFQEYVVLVAALTVACN 87

B. S100A2 isoform 1 EM**K**ELLH**K**ELPSFVGE**K**--VDEEGL**K**---**K**LMGSLDENSDQQVDFQEYAVFLALITVMCN 88

C. S100A2 isoform 2 EM**K**ELLH**K**ELPSFVGHS--REPCAVR---AFRVHL---------------FNPVIGDLRN 73

D. S100A2 isoform 3 -M**K**ELLH**K**ELPSFVGE**K**--VDEEGL**K**---**K**LMGSLDENSDQQVDFQEYAVFLALITVMCN 54

E. S100A3 EL**K**ELLQ**K**ELATWTPTE--FRECDYN---**K**FMSVLDTN**K**DCEVDFVEYVRSLACLCLYCH 87

F. S100A4 EL**K**ELLTRELPSFLG**K**R--TDEAAFQ---**K**LMSNLDSNRDNEVDFQEYCVFLSCIAMMCN 87

G. S100A5 EL**K**ELI**KK**ELCL--G-E--M**K**ESSID---DLM**K**SLD**K**NSDQEIDF**K**EYSVFLTMLCMAYN 84

H. S100A6 EL**K**ELIQ**K**ELTI--GS**K**--LQDAEIA---RLMEDLDRN**K**DQEVNFQEYVTFLGALALIYN 85

I. S100A7 SLLTMM**K**ENFPNFLSACD**KK**GTNYLA---DVFE**KK**D**K**NED**KK**IDFSEFLSLLGDIATDYH 87

J. S100A7A SLLTMM**K**ENFPNFLSACD**KK**GIHYLA---TVFE**KK**D**K**NED**KK**IDFSEFLSLLGDIAADYH 87

K. S100A8 DL**KK**LLETECPQYIR**KK**------GAD---VWF**K**ELDINTDGAVNFQEFLILVI**K**MGVAAH 107

L. S100A9 EF**K**ELVR**K**DLQNFL**KK**EN-**K**NE**K**VIE---HIMEDLDTNAD**K**QLSFEEFIMLMARLTWASH 91

M. S100A10 DLRVLME**K**EFPGFLENQ--**K**DPLAVD---**K**IM**K**DLDQCRDG**K**VGFQSFFSLIAGLTIACN 84

N. S100A11 EFLSFMNTELAAFT**K**NQ--**K**DPGVLD---RMM**KK**LDTNSDGQLDFSEFLNLIGGLAMACH 92

O. S100A12 EL**K**QLLT**K**ELANTI**K**NI--**K**D**K**AVID---EIFQGLDANQDEQVDFQEFISLVAIAL**K**AAH 86

P. S100A13 EF**K**ELVTQQLPHLL**K**-----DVGSLD---E**K**M**K**SLDVNQDSEL**K**FNEYWRLIGELA**K**EIR 88

Q. S100A14 ELRDLVTQQLPHLMP-----SNCGLE---E**K**IANLGSCNDS**K**LEFRSFWELIGEAA**K**SV**K** 96

R. S100A16 SFREMLQ**K**ELNHMLSDT--GNR**K**AAD---**K**LIQNLDANHDGRISFDEYWTLIGGITGPIA 91

I. S100B EL**K**ELINNELSHFLEEI--**K**EQEVVD---**K**VMETLDNDGDGECDFQEFMAFVAMVTTACH 86

II. S100G EL**K**LLIQAEFPSLL**K**GP-----NTLD---DLFQELD**K**NGDGEVSFEEFQVLV**KK**ISQ--- 79

III. S100P EL**K**VLME**K**ELPGFLQSG--**K**D**K**DAVD---**K**LL**K**DLDANGDAQVDFSEFIVFVAAITSACH 86

IV. S100Z EL**K**LLLQRELTEFLSCQ--**K**ETQLVD---**K**IVQDLDAN**K**DNEVDFNEFVVMVAALTVACN 87

1. Trichohyalin like protein 1 EL**K**QLIQGEFGDFFQ------PCVLHAVE**K**NSNLLNIDSNGIISFDEFVLAIFNLLNLCY 85

2. Trichohyalin DL**K**NLLEREFGAVLRRP--HDP**K**TVD---LILELLDLDSNGRVDFNEFLLFIF**K**VAQACY 86

3. Cornulin EL**K**RLLEQEFADVIV**K**P--HDPATVD---EVLRLLDEDHTGTVEF**K**EFLVLVF**K**VAQACF 86

4. Repetin EL**K**QLLLAEFGDILQRP--NDPETVE---TILNLLDQDRDGHIDFHEYLLLVFQLVQACY 86

5. Filaggrin 2 EL**K**ELLE**K**ELHPVL**K**NP--DDPDTVD---VIMHMLDRDHDRRLDFTEFLLMIF**K**LTMACN 86

6. Hornerin EL**K**ELLENEFHQIL**K**NP--NDPDTVD---IILQSLDRDHN**KK**VDFTEYLLMIF**K**LVQARN 86

7. Filaggrin EL**K**ELLE**K**EFRQIL**K**NP--DDPDMVD---VFMDHLDIDHN**KK**IDFTEFLLMVF**K**LAQAYY 86

**Figure E4 continued**

A. S100A1 NFFWENS----------------------------------------------------- 94

B. S100A2 isoform 1 DFFQGCPDRP-------------------------------------------------- 98

C. S100A2 isoform 2 QSPEG**K**SDCP**K**ITQHWR**K**WMRRG------------------------------------- 96

D. S100A2 isoform 3 DFFQGCPDRP-------------------------------------------------- 64

E. S100A3 EYF**K**DCPSEPPCSQ---------------------------------------------- 101

F. S100A4 EFFEGFPD**K**QPR**KK**---------------------------------------------- 101

G. S100A5 DFFLEDN**K**---------------------------------------------------- 92

H. S100A6 EAL**K**G------------------------------------------------------- 90

I. S100A7 **K**QSHGAAPCSGGSQ---------------------------------------------- 101

J. S100A7A **K**QSHGAAPCSGGSQ---------------------------------------------- 101

K. S100A8 **KK**SHEESH**K**E-------------------------------------------------- 117

L. S100A9 E**K**MHEGDEGPGHHH**K**PGLGEGTP------------------------------------- 114

M. S100A10 DYFVVHM**K**Q**K**G**KK**----------------------------------------------- 97

N. S100A11 DSFL**K**AVPSQ**K**RT----------------------------------------------- 105

O. S100A12 YHTH**K**E------------------------------------------------------ 92

P. S100A13 **KKK**DL**K**IR**KK**-------------------------------------------------- 98

Q. S100A14 LERPVRGH---------------------------------------------------- 104

R. S100A16 **K**LIHEQEQQSSS------------------------------------------------ 103

I. S100B EFFEHE------------------------------------------------------ 92

II. S100G ------------------------------------------------------------ 79

III. S100P **K**YFE**K**AGL**K**--------------------------------------------------- 95

IV. S100Z DYFVEQL**KKK**G**K**------------------------------------------------ 99

1. Trichohyalin like protein 1 LDI**K**SLLSSELRQVT--------------------------------------------- 100

2. Trichohyalin YALGQATGLDEE**K**R---------------------------------------------- 100

3. Cornulin **K**TLSESAEGACGSQ---------------------------------------------- 100

4. Repetin H**K**LDN**K**SHGGRTSQ---------------------------------------------- 100

5. Filaggrin 2 **K**VLS**K**EYC**K**ASGS**K**---------------------------------------------- 100

6. Hornerin **K**IIG**K**DYCQVSGS**K**---------------------------------------------- 100

7. Filaggrin ESTR**K**ENLPISGH**K**---------------------------------------------- 100

**Figure E4 continued**

**
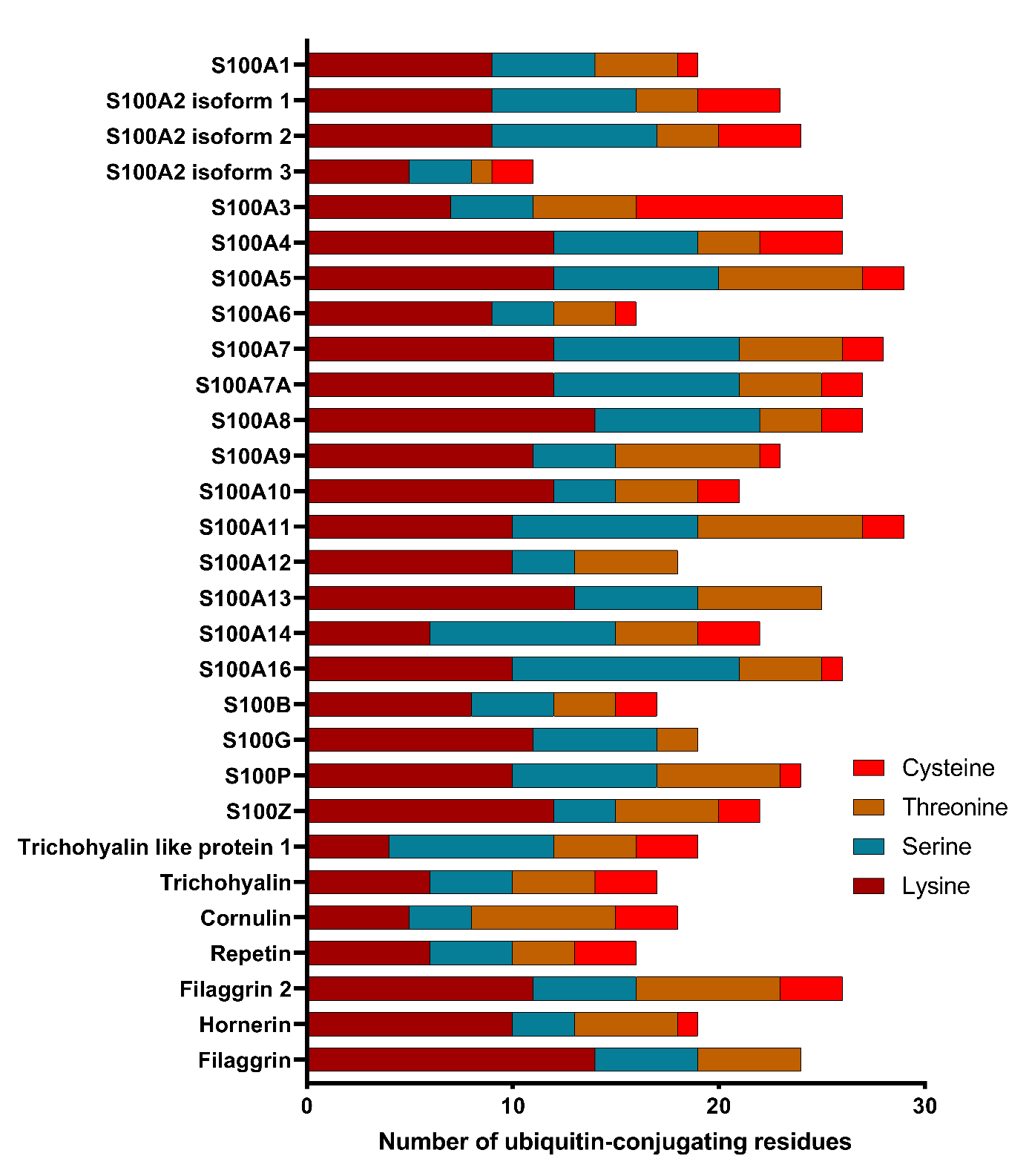
**

**Figure E5: Comparison of the number of ubiquitin-conjugating residues in S100 calcium-binding proteins and S100 domains of the S100 fused-type proteins.**


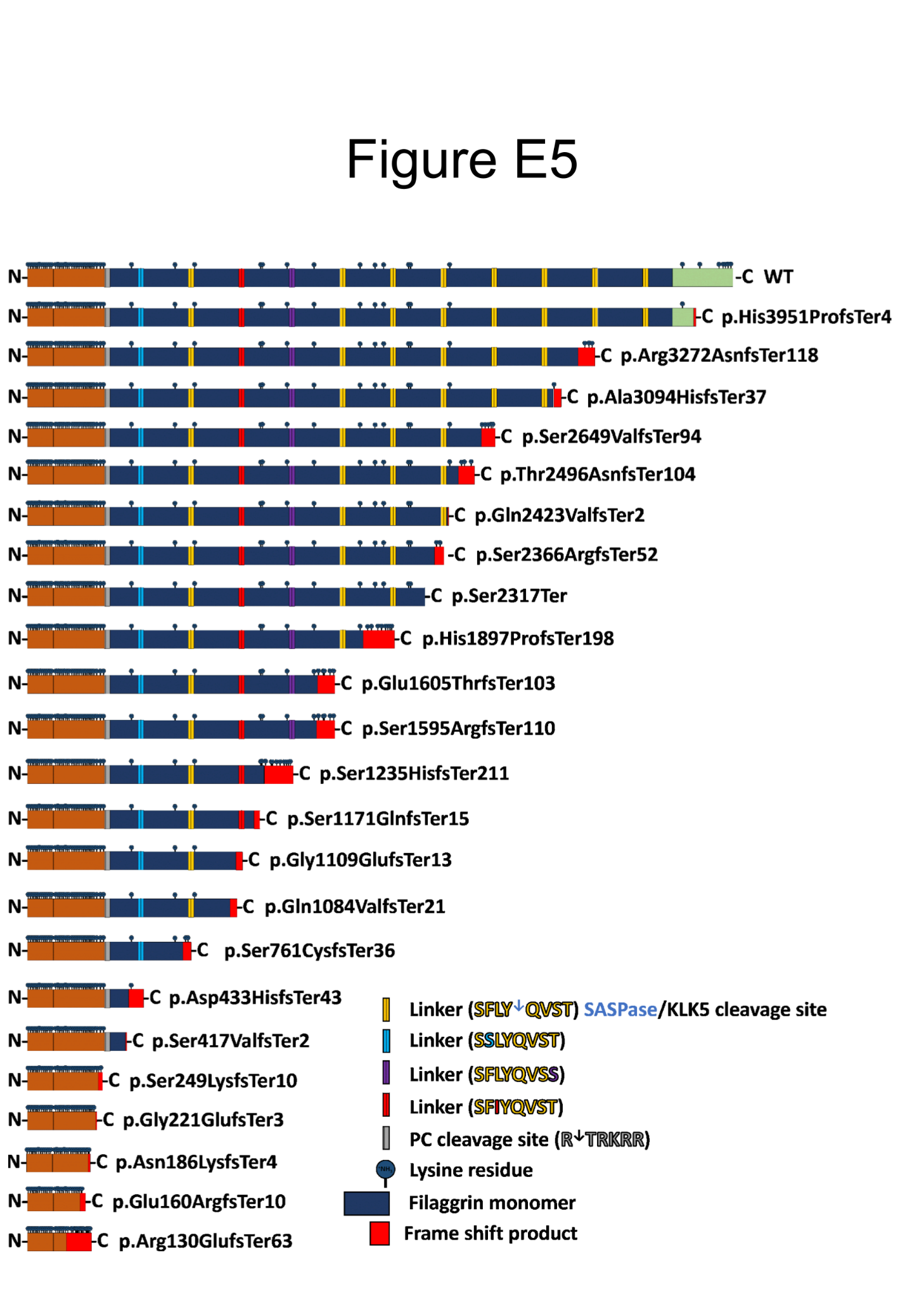


**Figure E6: Schematic illustration of 23 recurrent pathogenic frameshift mutation products of FLG showing alternation of Lys residues as compared to the wild-type.**


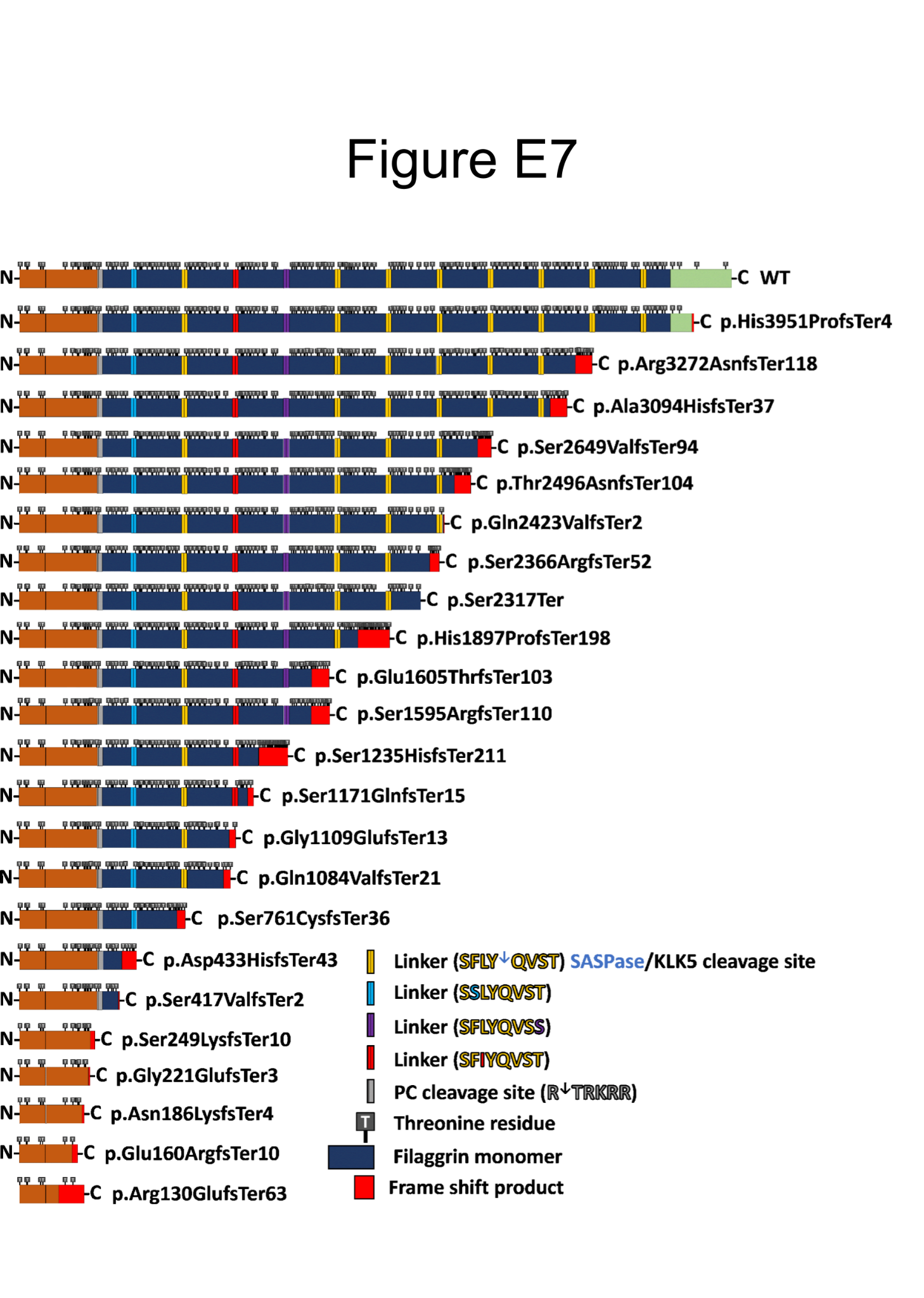
**Figure E7: Schematic illustration of 23 recurrent pathogenic frameshift mutation products of FLG showing alternation of Thr residues as compared to the wild-type.**


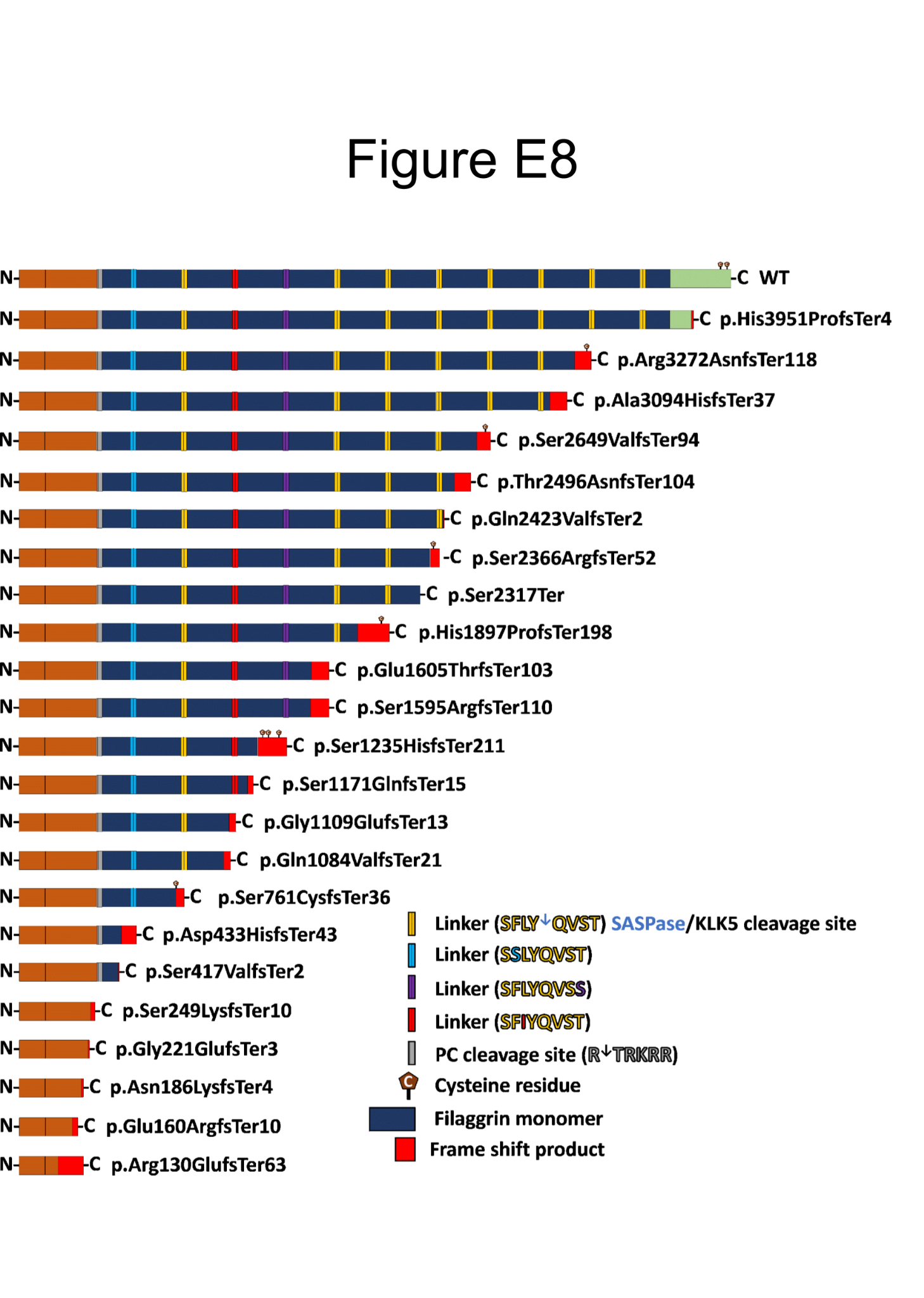
**Figure E8: Schematic illustration of 23 recurrent pathogenic frameshift mutation products of FLG showing alternation of Cys residues as compared to the wild-type.**


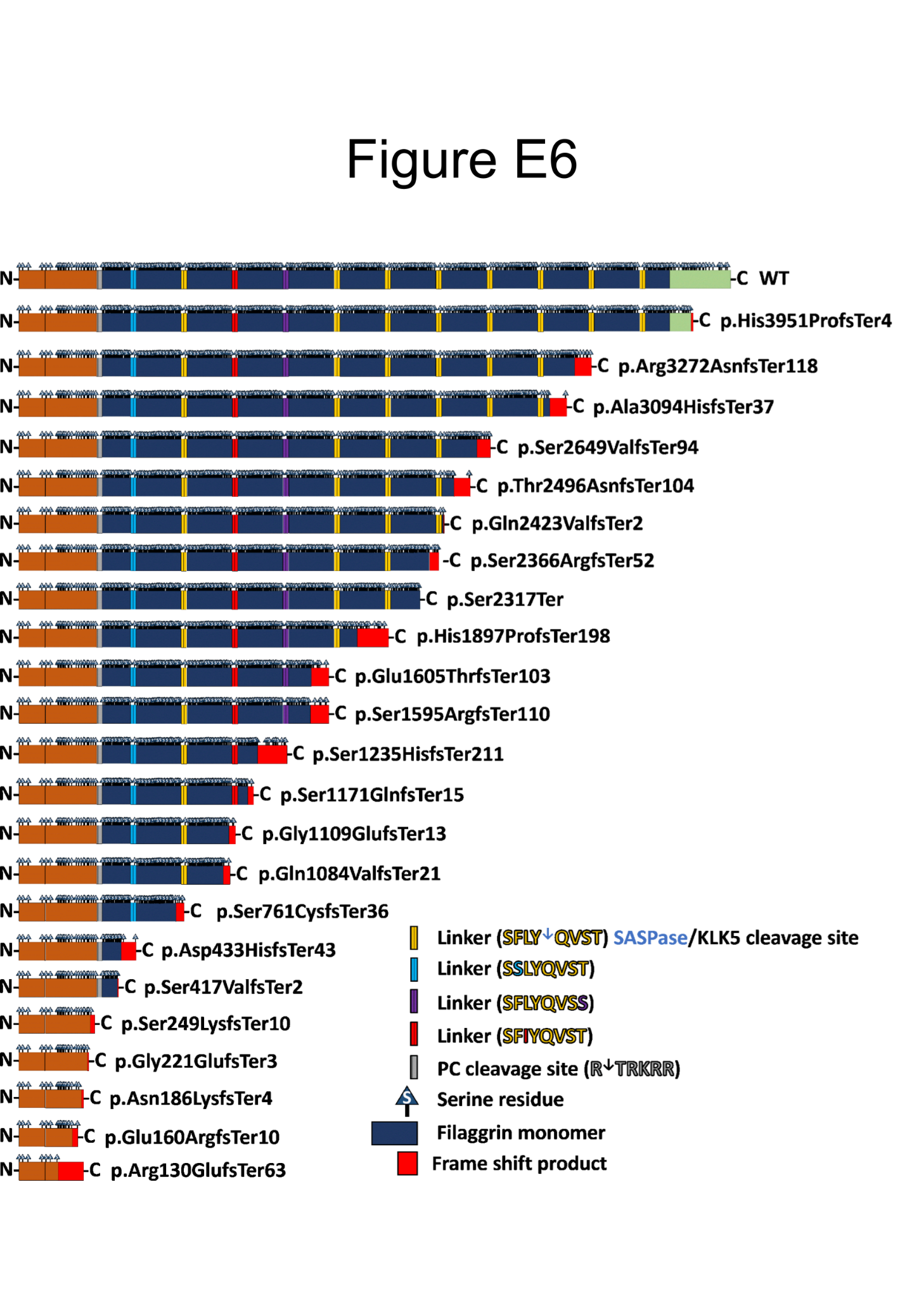
**Figure E9: Schematic pathogenic frameshift mutation products of FLG showing alternation of Ser residues as compared to the wild type**.
